# Supplementary material for: Computational Investigation of Conformational Properties of Short Azapeptides: Insights from DFT Study and NBO Analysis
Source: Molecules. 2023 Jul 17;28(14):5454. doi: 10.3390/molecules28145454 (PMC10386235; doi:10.3390/molecules28145454)
Supplement: Supplementary file 1 [file molecules-28-05454-s001.zip › molecules-2470977-supplementary.pdf]

Supporting Information:

Computational Investigation of Conformational Properties of Short Azapeptides: Insights from DFT Study and NBO Analysis

Table S1. Dihedral angle values (in degrees) of all the optimized structures of Ac-azaAsn-Pro-NHMe (**1 ANP**) at the B3LYP/6-311++G(d,p) level of theory in the gas phase.

	ω_0	φ_1	ψ_1	ω_1	φ_2	ψ_2	ω_2	χ_1	χ_2	χ_3	χ_4	χ_1'	χ_2'	Conformer
tttANP-1	-171.3	-62.5	-29.4	177.8	-103.2	17.2	174.4	33.8	-35.6	23.7	-2.3	64.9	167.7	$\alpha_R\delta_R(g^+)$
tctANP-2	-171.5	-126.1	27.7	23.3	-102.5	-13.7	178.6	31.3	-38.5	30.6	-11.3	69.8	158.7	$\delta_R\delta_R(g^+)$
tttANP-3	-170.9	-77.2	-33.7	-176.2	-90.0	74.2	-173.3	28.8	-35.8	28.5	-10.7	65.5	171.5	$\delta_R\gamma'(g^+)$
tttANP-4	-173.3	-63.4	-26.9	177.7	-102.3	16.0	174.5	34.2	-35.7	23.5	-1.8	122.0	78.8	$\alpha_R\delta_R(s^+)$
ttANP-5	173.2	118.5	-29.2	-173.5	-90.2	78.1	-177.0	28.8	-35.7	28.5	-10.7	115.8	-19.3	$\delta_L\gamma'(s^+)$
cttANP-6	11.8	-124.3	28.5	-175.4	-84.0	73.2	-177.9	34.3	-39.2	28.3	-7.0	71.7	161.7	$\delta_R\gamma'(g^+)$
tctANP-7	-166.1	102.1	-169.5	30.1	-119.1	-0.6	177.5	37.2	-38.3	24.7	-1.2	85.7	4.5	$\varepsilon\delta_R(g^+)$
tttANP-8	-171.0	-61.6	-32.6	179.5	-101.6	18.4	174.1	33.9	-36.1	24.4	-3.0	72.3	26.4	$\alpha_R\delta_R(g^+)$
tttANP-9	168.9	-140.4	176.8	178.1	-108.1	24.8	173.9	35.0	-34.1	20.2	2.0	70.2	-131.9	$\beta_S\delta_R(g^+)$
cttANP-10	9.5	-128.0	38.9	-173.2	-82.1	69.9	-178.9	35.9	-39.5	27.2	-4.7	63.7	50.6	$\delta_R\gamma'(g^+)$
cttANP-11	-10.5	119.9	-16.0	-173.2	-88.1	74.5	-177.3	31.8	-37.8	28.6	-8.9	114.9	74.2	$\delta_L\gamma'(s^+)$
tttANP-12	-179.0	131.0	-43.7	146.7	-68.1	162.2	-178.5	25.3	-35.8	32.2	-17.2	116.2	-21.4	$\delta_L\beta_P(s^+)$
tctANP-13	175.3	124.9	-82.5	-7.8	-68.8	136.4	179.3	31.0	-36.5	27.3	-8.0	74.9	20.8	$?\beta_P(g^+)$
tctANP-14	169.6	133.0	-77.4	-10.8	-60.8	141.1	-179.6	20.1	-36.2	28.8	-11.0	168.1	-10.8	$?\beta_P(t)$
tttANP-15	173.5	-81.0	160.4	170.6	-82.3	156.1	179.9	25.6	-35.7	31.7	-16.3	67.1	102.1	$\beta_P\beta_P(g^+)$
cctANP-16	4.7	-88.8	-7.2	-20.0	-52.0	166.2	179.5	30.9	-37.5	29.6	-10.6	74.2	17.4	$\delta_R\varepsilon''(g^+)$
cctANP-17	15.7	-121.9	10.4	3.4	-88.4	15.0	177.4	36.1	-38.7	26.1	-3.4	76.7	174.0	$\delta_R\delta_R(g^+)$
ctANP-18	7.0	-96.9	-9.8	176.7	-115.1	6.0	177.5	35.2	-36.7	24.3	-2.1	67.0	179.4	$\delta_R\delta_R(g^+)$
tcANP-19	-175.0	-72.3	-37.2	-24.8	-39.5	-60.9	177.3	21.1	-32.6	31.3	-19.2	73.2	16.2	$\alpha_R?(g^+)$
ctANP-20	-16.8	-106.7	170.6	-179.1	107.6	17.5	175.0	36.1	-35.3	20.9	2.1	71.7	-90.7	$\varepsilon''\delta_L(g^+)$
ccANP-21	11.6	-79.8	-25.9	-33.7	-72.8	78.7	-177.7	36.0	-26.6	6.6	17.0	68.2	-170.7	$\delta_R\beta(g^+)$
ccANP-22	21.1	-137.3	41.6	32.1	-107.6	-13.6	174.8	33.0	-39.3	30.3	-9.9	60.5	49.5	$?\delta_R(g^+)$
cttANP-23	4.7	-86.7	-18.9	-178.1	-110.3	5.8	177.5	34.4	-37.2	25.8	-4.2	111.3	79.7	$\delta_R\delta_R(s^+)$
tctANP-24	-169.7	-73.8	-37.1	-26.8	-51.8	144.7	-178.3	31.4	-36.5	27.4	-7.9	65.6	165.6	$\alpha_R\beta_P(g^+)$
tctANP-25	173.3	145.4	-64.4	-6.3	-85.6	34.5	174.9	37.7	-36.9	21.4	2.7	164.4	158.9	$?\delta_R(t)$
cttANP-26	3.9	-79.2	-26.7	-175.9	-108.6	7.3	176.1	34.5	-37.6	26.2	-4.6	71.6	27.6	$\delta_R\delta_R(g^+)$
tttANP-27	173.0	136.2	-52.3	-172.3	-89.4	75.9	-176.9	27.8	-35.2	28.5	-11.4	158.7	154.4	$?\gamma'(t)$
ttcANP-28	-175.5	-73.7	-28.9	179.1	-118.8	76.5	-7.2	35.7	-30.7	14.0	8.8	122.9	78.2	$\delta_R\zeta(s^+)$
tccANP-29	-168.5	-123.2	26.1	22.0	-97.5	-20.1	7.6	31.1	-37.8	29.7	-10.5	74.1	95.5	$\delta_R\delta_R(g^+)$
tttANP-30	-178.4	148.2	-52.3	150.4	-66.5	161.7	-178.7	27.0	-36.0	30.8	-14.5	164.1	154.0	$?\beta_P(t)$
cctANP-31	-19.2	-112.1	163.8	-21.9	-73.8	-5.5	-178.1	34.1	-36.5	24.3	-2.7	69.9	-75.0	$\varepsilon''\delta_R(g^+)$
cccANP-32	-1.6	138.0	-38.2	-22.7	-53.0	168.8	-7.1	32.2	-36.9	27.2	-7.1	134.2	-0.4	$\delta_L\varepsilon''(s^+)$
cccANP-33	-21.6	142.0	-45.7	-10.1	-71.2	-19.1	11.4	32.3	-36.9	26.8	-6.6	152.4	143.1	$\delta_L\delta_R(t)$

Table S2. Dihedral angle values (in degrees) of all the optimized structures of Ac-azaAsp-Pro-NHMe **(2, ADP**)at the B3LYP/6-311++G(d,p) level of theory in the gas phase.

	ω_0	φ_1	ψ_1	ω_1	φ_2	ψ_2	ω_2	χ_1	χ_2	χ_3	χ_4	χ_1'	χ_2'	Conformer
tttADP-1	-173.3	-61.6	-30.6	178.2	-103.4	18.3	174.2	33.9	-35.7	23.7	-2.3	63.8	177.1	$\alpha_R\delta_R(g^+)(\beta I)$
tttADP-2	-172.3	-75.9	-35.0	-176.4	-90.0	75.0	-173.3	28.9	-35.7	28.3	-10.4	65.1	177.6	$\alpha_R\gamma'(g^+)$
tctADP-3	-172.5	-125.1	25.7	24.6	-102.8	-14.1	-180.0	31.0	-38.4	30.8	-11.7	66.2	173.3	$?\delta_R(g^+)$
tctADP-4	-167.0	115.6	-165.1	24.4	-104.7	150.9	-178.2	34.6	-37.0	24.8	-2.9	93.7	-2.8	$\varepsilon\beta_S(g^+)$
cttADP-5	10.1	-124.9	30.8	-175.0	-83.2	73.5	-178.2	34.8	-39.5	28.3	-6.6	69.2	170.8	$?\gamma'(g^+)$
tttADP-6	-173.4	-63.1	-27.4	178.3	-102.1	16.4	174.6	34.6	-35.4	22.4	-0.4	131.2	66.5	$\delta_R\delta_R(s^+)$
tttADP-7	175.1	121.3	-29.5	-174.4	-90.1	79.0	-177.1	28.4	-35.8	29.1	-11.6	116.2	-28.6	$?\gamma'(s^+)$
tttADP-8	168.7	-132.1	178.3	-179.9	-108.6	26.1	173.4	34.8	-34.3	20.7	1.4	79.8	-157.8	$\varepsilon''\delta_R(g^+)$
tttADP-9	-178.0	136.7	-44.9	145.8	-67.8	162.6	-178.6	26.4	-36.1	31.5	-15.7	117.2	-34.4	$?\beta_P(s^+)$
cttADP-10	4.6	-91.8	-13.6	178.2	-114.6	6.2	177.3	35.2	-36.8	24.4	-2.3	66.8	-176.5	$\delta_R\delta_R(g^+)$
cttADP-11	9.0	-126.7	38.4	-172.7	-82.7	71.3	-179.2	35.9	-39.4	27.0	-4.5	71.9	58.9	$?\gamma'(g^+)$
cctADP-12	15.3	-121.9	10.2	5.3	-88.4	11.2	179.0	35.1	-38.8	27.2	-5.2	74.8	179.6	$\delta_R\delta_R(g^+)$
tctADP-13	-166.6	112.0	-170.7	30.4	-120.1	1.6	177.0	37.9	-38.3	24.0	-0.1	92.7	-6.4	$\varepsilon\delta_R(s^+)$
tttADP-14	173.9	136.0	-50.1	-172.8	-89.5	76.6	-176.9	27.6	-35.2	28.7	-11.7	149.4	174.0	$?\gamma'(s^+)$
cctADP-15	10.3	-75.5	-31.2	-28.0	-73.7	73.2	-178.6	36.5	-30.6	12.3	11.4	73.2	-171.1	$\alpha_R\gamma'(g^+)$
tctADP-16	173.6	145.4	-61.0	-6.6	-83.3	27.1	173.8	37.1	-37.0	22.0	1.7	157.6	171.5	$?\delta_R(t)$
tctADP-17	-170.8	-73.8	-37.8	-26.7	-52.5	174.6	-179.8	31.7	-36.4	26.8	-7.0	63.5	175.0	$\alpha_R\beta_P(g^+)$
cctADP-18	22.0	-137.0	41.3	29.7	-104.8	-15.7	175.8	32.8	-39.3	30.2	-9.9	68.0	59.4	$?\delta_R(g^+)$
cctADP-19	3.1	-81.6	-17.1	-24.9	-45.8	167.4	177.7	29.8	-37.7	31.1	-12.9	74.6	26.4	$\delta_R\varepsilon''(g^+)$
tctADP-20	-176.0	-73.8	-37.3	-24.4	-39.3	-69.5	176.9	21.6	-32.7	31.0	-18.5	78.2	11.3	$\alpha_R\alpha_R(g^+)$
tttADP-21	-178.0	147.1	-52.2	149.9	-66.1	161.2	-178.5	26.8	-35.9	30.8	-14.6	156.9	172.9	$?\beta_P(t)$
tctADP-22	170.1	133.1	-72.2	-16.6	-52.2	153.3	-178.4	29.0	-36.7	29.8	-12.1	166.1	-10.1	$\gamma\beta_P(t)$
cttADP-23	3.6	-85.1	-19.5	-176.3	-108.6	6.0	176.9	34.8	-38.0	26.5	-4.6	121.2	66.8	$\delta_R\delta_R(s^+)$
ttcADP-24	-176.3	-71.1	-30.0	179.0	-117.3	79.3	-7.9	35.4	-29.9	12.9	9.8	132.9	66.0	$\alpha_R\zeta(s^+)$
tccADP-25	-169.7	-118.6	22.9	21.1	-95.2	-19.1	4.0	31.1	-37.5	29.0	-9.7	85.6	63.2	$\delta_R\delta_R(g^+)$
cttADP-26	-19.9	-114.1	176.5	179.2	-111.6	15.2	175.2	36.3	-35.1	20.4	2.6	68.9	-65.1	$\varepsilon''\delta_R(g^+)$
cttADP-27	-5.7	-69.3	-33.8	-174.2	-108.8	6.2	176.6	35.0	-37.4	25.3	-3.2	78.5	25.6	$\alpha_R\delta_R(g^+)(\beta I)$
cccADP-28	-0.6	139.0	-38.0	-24.23	-48.6	170.1	-8.2	31.0	-37.2	28.9	-9.7	126.4	8.5	$\delta_L\varepsilon''(s^+)$
cctADP-29	-24.5	-113.4	166.9	-28.9	-65.7	-9.4	-177.9	34.2	-36.3	23.8	-2.1	57.3	-47.3	$\varepsilon''\delta_R(g^+)$
cccADP-30	-21.8	143.7	-42.6	-11.8	-68.5	-20.8	4.7	31.1	-36.0	26.5	-7.1	143.5	70.9	$\delta_L\delta_R(s^+)$

Table S3. Dihedral angle values (in degrees) of all the optimized structures of Ac-azaAla-Pro-NHMe (**3, AAP)** at the B3LYP/6-311++G(d,p) level of theory in the gas phase.

	ω_0	φ_1	ψ_1	ω_1	φ_2	ψ_2	ω_2	χ_1	χ_2	χ_3	χ_4	Conformer
tttAAP-1	-175.5	-57.8	-35.1	-179.9	-102.9	19.4	173.9	33.8	-36.0	24.4	-3.2	$\alpha_R\delta_R$(βI)
tttAAP-2	-174.9	-69.3	-40.6	-175.4	-90.2	74.3	-173.3	28.5	-35.5	28.4	-10.8	$\alpha_R\gamma'$
tttAAP-3	170.4	129.7	-37.9	-172.8	-90.4	76.6	-176.7	27.8	-35.3	28.8	-11.6	$\delta_L\gamma'$
tttAAP-4	170.2	-125.2	169.6	-179.1	-88.0	77.2	-176.3	24.1	-35.9	33.3	-19.0	$\varepsilon''\gamma'$
tctAAP-5	169.6	136.8	-65.9	-17.4	-69.2	113.0	-178.2	32.8	-33.6	20.9	-0.1	$\delta_L\varepsilon''$
cttAAP-6	-10.9	131.9	-35.3	-173.4	-88.6	75.9	-176.9	29.2	-36.7	29.6	-11.6	$\delta_L\gamma'$
cttAAP-7	11.4	-131.9	40.4	-174.3	-81.1	70.7	-178.6	35.6	-39.5	27.5	-5.2	?γ'
tctAAP-8	-171.6	-123.6	24.1	27.3	-104.8	-15.0	-178.3	30.8	-38.6	31.5	-12.6	$\delta_R\delta_R$
tttAAP-9	170.3	-128.4	173.1	-176.5	-116.6	13.3	174.6	35.4	-36.1	23.2	-0.9	$\beta_S\delta_R$
tctAAP-10	-176.2	-73.3	-35.7	-14.8	-46.3	-47.9	176.2	19.9	-32.9	33.0	-21.7	$\alpha_R\alpha_R$
cttAAP-11	2.6	-77.9	-29.8	-175.5	-111.6	8.2	176.7	34.8	-37.2	25.4	-3.5	$\delta_R\delta_R$
tctAAP-12	-170.5	129.7	-173.2	32.3	-119.9	-2.4	179.3	36.3	-38.2	25.6	-2.8	$\varepsilon\delta_R$
cctAAP-13	24.3	-142.0	43.1	31.3	-108.0	-14.9	175.2	32.1	-38.9	30.7	-10.8	?δ_R
tttAAP-14	179.0	141.6	-44.3	148.6	-67.3	159.5	-178.6	25.3	-35.8	32.2	-17.2	?β_P
cctAAP-15	-2.7	-109.0	17.6	-2.5	-80.7	144.2	178.5	33.6	-36.4	24.9	-3.7	$\delta_R\beta_P$
cttAAP-16	-14.9	-103.1	170.0	-174.7	-110.6	13.2	173.9	34.9	-36.8	24.7	-2.7	$\varepsilon''\delta_R$
cctAAP-17	-15.2	-112.2	163.1	-20.7	-74.9	-3.7	-178.9	35.0	-36.2	22.9	-0.7	$\beta_S\delta_R$
ttcAAP-18	-178.6	-67.2	-35.2	179.7	-117.7	79.8	-7.8	34.8	-30.1	13.9	8.3	$\alpha_R\zeta$
cctAAP-19	15.3	-69.9	-43.4	-13.0	-75.0	53.0	-179.1	36.0	-36.0	21.6	1.2	$\alpha_R\gamma'$
cccAAP-20	-13.6	123.9	-15.0	1.7	-76.0	163.5	-11.7	34.2	-37.4	26.0	-4.5	$\delta_L\beta_P$
tccAAP-21	-170.7	-124.2	25.0	23.7	-99.5	-18.1	1.5	30.8	-37.7	29.9	-10.9	$\delta_R\delta_R$
cccAAP-22	-21.5	144.4	-49.6	-13.2	-69.9	-19.9	0.4	31.2	-35.2	25.3	-5.8	$\delta_L\delta_R$

Table S4. Dihedral angle values (in degrees) of all the optimized structures of Ac-azaAsn-Pro-NHMe at the SMD/B3LYP/6-311++G(d,p) level of theory in water.

	ω_0	φ_1	ψ_1	ω_1	φ_2	ψ_2	ω_2	χ_1	χ_2	χ_3	χ_4	χ_1'	χ_2'	Conformer
tttANP-1	-178.5	-61.7	-33.1	-179.6	-92.5	2.9	175.9	31.8	-36.6	27.1	-7.2	66.4	-165.3	$\alpha_R\delta_R(g^+)(\beta I)$
tctANP-2	-169.5	-129.9	31.9	20.5	-101.1	-11.2	-179.9	33.4	-39.0	29.3	-8.5	75.7	167.1	$?\delta_R(g^+)$
tttANP-3	-177.5	-70.7	-34.0	-171.5	-92.3	74.6	-175.0	29.1	-36.5	29.3	-11.5	64.1	-158.1	$\delta_R\gamma'(g^+)$
tttANP-4	-178.4	-67.3	-30.9	-178.6	-98.7	4.4	177.6	34.2	-35.6	23.1	-1.4	123.9	63.1	$\alpha_R\delta_R(s^+)(\beta I)$
ttANP-5	175.8	113.2	-14.7	-174.1	-89.5	71.4	-177.3	30.5	-37.5	29.5	-10.8	124.0	-32.7	$\delta_L\gamma'(s^+)$
cttANP-6	6.7	-122.7	29.9	-173.2	-84.5	59.4	-179.2	36.0	-40.9	29.2	-6.9	76.9	165.3	$?\gamma'(g^+)$
tctANP-7	-172.9	96.1	-168.6	26.8	-111.0	-13.3	-179.3	34.9	-38.1	26.4	-4.5	82.0	3.4	$\varepsilon\delta_R(g^+)$
tttANP-8	-178.8	-61.2	-36.2	-179.3	-96.4	7.0	177.4	33.5	-35.7	23.8	-3.0	76.3	16.6	$\alpha_R\delta_R(g^+)(\beta I)$
tttANP-9	-177.1	-123.9	173.5	179.1	-96.0	12.4	175.2	32.4	-37.2	27.4	-7.2	74.9	-148.1	$\beta_S\delta_R(g^+)$
cttANP-10	7.1	-126.1	38.4	-171.7	-82.2	56.4	178.5	36.5	-41.5	29.6	-6.9	64.1	52.2	$\beta\gamma'(g^+)$
cttANP-11	-7.4	112.6	-9.8	-173.5	-89.0	69.6	-177.0	32.0	-38.2	29.0	-9.3	112.7	51.0	$\delta_L\gamma'(s^+)$
tttANP-12	-179.8	79.0	14.2	-177.4	-62.8	149.1	175.6	33.8	-36.9	25.5	-4.2	102.4	-19.9	$\delta_L\beta_P(s^+)$
tctANP-13	173.5	124.6	-20.5	-27.7	-63.2	175.2	177.4	37.5	-34.5	18.1	5.9	126.9	-33.6	$\delta_L\varepsilon''(s^+)$
tctANP-14	172.3	134.2	-38.8	-25.5	-59.5	-179.9	-179.4	37.4	-36.3	20.9	3.0	137.9	-10.3	$?\beta_P(g^+)$
tttANP-15	175.3	-92.2	161.5	170.3	-71.6	148.6	175.7	24.3	-35.3	32.1	-17.7	61.9	82.6	$\varepsilon''\beta_P(g^+)$
cctANP-16	7.9	-96.1	-9.4	-21.1	-58.0	174.9	178.9	34.8	-36.6	24.1	-2.1	91.2	6.6	$\delta_R\varepsilon''(s^+)$
cctANP-17	13.5	-124.4	21.4	8.9	-86.9	-12.9	177.4	31.9	-38.0	29.1	-9.3	78.2	170.1	$\delta_R\delta_R(g^+)$
ctANP-18	6.7	-107.6	9.3	179.9	-84.8	-8.1	179.7	31.4	-37.3	28.4	-8.9	80.9	175.5	$\delta_R\delta_R(g^+)$
tcANP-19	-177.7	-63.3	-44.8	-16.8	-62.7	-22.8	180.0	30.5	-35.4	26.2	-7.1	77.7	17.4	$\alpha_R\delta_R(g^+)$
ctANP-20	-8.1	-95.3	171.2	179.2	-95.3	12.5	175.8	32.5	-37.5	27.8	-7.5	74.9	-146.3	$\varepsilon''\delta_R(g^+)$
ccANP-21	8.4	-85.6	-27.0	-40.8	-69.9	86.1	-176.2	30.7	-14.4	-7.8	28.6	63.7	-163.1	$\delta_R\gamma'(g^+)$
ccANP-22	13.7	-130.4	30.3	17.0	-95.0	-14.0	-178.9	32.6	-38.0	28.4	-8.2	66.9	55.8	$?\delta_R(g^+)$
cttANP-23	0.5	-84.4	-18.7	-177.9	-97.3	0.6	178.6	33.2	-36.4	25.4	-4.5	111.2	54.4	$\delta_R\delta_R(s^+)$
tctANP-24	-177.3	-64.9	-40.3	-22.1	-59.8	151.4	175.6	32.4	-34.9	23.6	-3.1	61.2	-153.2	$\alpha_R\beta_P(g^+)$
tctANP-25	173.2	133.9	-40.0	-10.3	-71.7	-11.7	-179.8	33.1	-36.0	24.5	-3.7	152.6	151.9	$?\delta_R(t)$
cttANP-26	0.2	-81.6	-20.9	179.1	-96.5	-1.4	179.3	32.3	-35.7	25.1	-4.8	94.9	5.0	$\delta_R\delta_R(s^+)$
tttANP-27	174.9	129.1	-36.1	-174.2	-92.0	80.3	-175.6	28.2	-35.6	28.8	-11.5	148.2	153.8	$\delta_L\gamma'(s^+)$
ttcANP-28	-179.1	-76.3	-27.9	-179.4	-119.1	80.0	-2.8	35.0	-29.0	11.8	10.6	120.5	66.4	$\delta_R\zeta(s^+)$
tccANP-29	-168.0	-128.8	32.6	19.0	-95.3	-17.5	5.8	32.9	-38.3	28.6	-8.1	70.2	54.0	$\beta\delta_R(g^+)$
tttANP-30	173.3	131.6	-42.8	-175.7	-79.5	145.7	178.1	23.8	-34.9	32.0	-17.8	155.5	151.9	$\gamma\beta_P(t)$
cctANP-31	0.7	-119.0	165.6	-26.2	-68.5	-13.7	178.6	34.6	-34.9	21.2	0.9	61.4	-157.2	$\beta_S\delta_R(g^+)$
cccANP-32	-6.8	140.9	-43.9	-23.7	-58.6	168.2	-8.3	34.0	-35.7	23.3	-1.7	146.8	-13.8	$\gamma\varepsilon''(s^+)$
cccANP-33	-8.3	134.1	-35.0	-7.8	-71.6	-18.7	-2.0	32.1	-34.7	23.6	-3.3	149.1	152.6	$\delta_L\delta_R(s^+)$

Table S5. Dihedral angle values (in degrees) of all the optimized structures of Ac-azaAsp-Pro-NHMe at the SMD/B3LYP/6-311++G(d,p) level of theory in water.

	ω_0	φ_1	ψ_1	ω_1	φ_2	ψ_2	ω_2	χ_1	χ_2	χ_3	χ_4	χ_1'	χ_2'	Conformer
tttADP-1	-178.7	-61.7	-33.6	-179.5	-91.8	2.8	176.3	31.8	-36.9	27.5	-7.6	69.9	-170.1	$\alpha_R\delta_R(g^+)$
tttADP-2	-176.8	-70.1	-35.2	-171.6	-92.0	69.5	-176.4	29.9	-36.9	29.1	-10.6	65.9	-169.7	$\alpha_R\gamma'(g^+)$
tctADP-3	-169.2	-131.0	33.3	21.2	-101.5	-12.9	-179.8	33.2	-38.8	-29.2	-8.6	72.8	175.9	$?\delta_R(g^+)$
tctADP-4	-173.4	116.3	-162.0	23.1	-105.0	154.0	-179.7	34.6	-37.4	25.3	-3.5	89.2	-5.1	$\varepsilon\beta_S(g^+)$
cttADP-5	5.5	-121.8	29.9	-172.8	-84.3	59.4	-179.5	35.9	-41.0	29.4	-7.1	77.7	170.6	$?\gamma'(g^+)$
tttADP-6	-177.0	-69.0	-28.2	-178.4	-94.7	3.1	178.0	34.1	-35.8	23.4	-1.9	134.7	59.9	$\delta_R\delta_R(s^+)$
tttADP-7	-179.1	80.1	25.4	-171.2	-81.4	49.0	177.5	37.4	-41.9	29.4	-6.1	104.6	-31.6	$\delta_L\beta(s^+)$
tttADP-8	-177.3	-121.9	173.7	179.2	-97.6	2.6	178.1	32.6	-36.3	25.8	-5.3	73.3	-172.5	$\varepsilon''\delta_R(g^+)$
tttADP-9	-178.9	82.2	21.7	-178.5	-61.2	148.9	175.6	33.6	-37.1	25.8	-4.6	106.1	-32.5	$\delta_L\beta_P(s^+)$
cttADP-10	6.4	-99.2	-2.2	-179.0	-87.4	-4.6	178.5	30.7	-36.8	28.4	-9.3	91.0	179.7	$\delta_R\delta_R(s^+)$
cttADP-11	6.3	-125.8	40.3	-170.5	-82.9	56.4	178.3	36.3	-41.4	29.6	-7.1	74.5	55.1	$\beta\gamma'(g^+)$
cctADP-12	13.2	-123.9	21.3	9.1	-87.4	-13.3	178.3	32.0	-38.1	29.0	-9.2	77.2	175.8	$?\delta_R(g^+)$
tctADP-13	-173.4	114.5	-166.0	27.2	-112.0	-11.3	-179.1	35.7	-38.0	25.5	-3.0	91.3	-7.6	$\varepsilon\delta_R(s^+)$
tttADP-14	175.5	128.0	-33.9	-174.6	-92.0	80.0	-175.3	28.3	-35.6	28.6	-11.3	141.8	167.0	$?\gamma'(s^+)$
cctADP-15	7.3	-86.9	-25.2	-39.8	-69.4	97.4	-175.0	30.3	-15.0	-6.3	26.8	65.5	-173.1	$\delta_R\varepsilon''(g^+)$
tctADP-16	171.1	132.3	-39.8	-12.2	-73.2	-6.6	178.1	34.5	-35.5	22.4	-0.5	147.8	164.1	$\delta_L\delta_R(s^+)$
tctADP-17	-174.8	-67.4	-39.6	-23.4	-59.8	153.8	175.5	33.9	-34.3	21.2	0.4	68.0	-170.9	$\alpha_R\beta_P(g^+)$
cctADP-18	12.9	-128.1	30.2	13.6	-91.1	-14.0	178.1	33.6	-38.1	27.4	-6.4	80.5	57.2	$?\delta_R(g^+)$
cctADP-19	7.0	-93.0	-14.4	-21.2	-55.7	-179.8	-179.2	34.9	-38.0	26.3	-4.4	90.1	10.0	$\delta_R\varepsilon'(g^+)$
tctADP-20	-177.5	-67.0	-47.6	-21.5	-45.8	-67.9	179.1	24.4	-32.5	27.5	-12.8	74.6	17.6	$\alpha_R\alpha_R(g^+)$
tttADP-21	174.0	129.4	-39.2	-176.6	-80.1	146.0	178.0	24.9	-34.9	31.0	-16.0	147.7	162.9	$\delta_L\beta_P(s^+)$
tctADP-22	172.4	133.7	-40.6	-25.6	-57.4	-174.2	-178.4	37.2	-37.1	22.5	1.2	137.7	-9.3	$\delta_L\varepsilon'(s^+)$
cttADP-23	7.1	-127.6	37.8	-174.6	-69.1	-23.2	-178.6	31.4	-37.5	28.4	-8.9	74.4	58.4	$?\delta_R(g^+)$
ttcADP-24	-175.6	127.6	41.6	-178.0	-63.2	152.7	-2.7	32.1	-37.6	27.9	-7.9	74.7	54.8	$\delta_L\beta_P(g^+)$
tccADP-25	-168.5	-129.1	34.5	16.2	-92.9	-17.9	4.9	33.1	-37.7	27.3	-6.5	78.7	56.4	$\beta\delta_R(g^+)$
cttADP-26	-8.3	-93.0	167.1	-178.6	-96.7	1.7	177.9	32.3	-36.4	26.3	-6.1	68.7	-169.4	$\varepsilon''\delta_R(g^+)$
cttADP-27	0.9	-76.2	-28.7	-178.4	-97.4	1.3	178.6	32.9	-36.1	25.0	-4.3	79.9	18.7	$\delta_R\delta_R(g^+)(\beta I)$
cccADP-28	-7.8	140.0	-49.1	-25.2	-55.0	176.5	-3.7	34.5	-36.3	23.7	-1.8	150.1	-11.2	$?\varepsilon''(s^+)$
cctADP-29	-1.5	-114.7	165.0	-26.8	-67.3	-14.6	178.0	33.9	-34.4	21.0	0.7	66.0	-172.6	$\beta_S\delta_R(g^+)$
cccADP-30	-10.4	98.8	21.7	23.7	-95.6	-22.4	4.7	31.9	-36.8	27.1	-7.2	102.2	51.1	$\delta_L\delta_R(s^+)$

Table S6. Dihedral angle values (in degrees) of all the optimized structures of Ac-azaAla-Pro-NHMe at the SMD/B3LYP/6-311++G(d,p) level of theory in water.

	ω_0	φ_1	ψ_1	ω_1	φ_2	ψ_2	ω_2	χ_1	χ_2	χ_3	χ_4	Conformer
tttAAP-1	-178.0	-60.3	-37.7	-178.3	-94.8	4.9	178.0	33.5	-36.5	25.1	-4.0	$\alpha_R\delta_R(\beta I)$
tttAAP-2	-177.9	-66.4	-37.6	-171.9	-91.7	71.3	-178.4	30.4	-35.8	26.9	-8.1	$\alpha_R\gamma'$
tttAAP-3	173.6	130.4	-41.5	174.2	-91.8	76.1	-175.4	28.4	-35.6	28.6	-11.3	$?\gamma'$
tttAAP-4	177.2	-90.8	163.7	178.6	-78.2	143.3	176.2	24.2	-35.4	32.4	-18.0	$\beta_P\beta_P$
tctAAP-5	170.8	136.5	-46.7	-24.6	-68.9	125.5	-179.9	32.2	-27.5	11.9	9.1	$\delta_L\varepsilon''$
cttAAP-6	-5.9	126.1	-29.7	-171.4	-91.2	69.1	-176.8	30.5	-37.0	28.7	-9.9	$\delta_L\gamma'$
cttAAP-7	7.6	-130.1	38.9	-173.5	-81.5	48.6	176.7	37.6	-41.5	28.7	-5.2	$?\gamma'$
tctAAP-8	-168.3	-132.7	33.9	20.1	-101.1	-13.3	-179.0	32.6	-38.3	29.1	-8.8	$\beta\delta_R$
tttAAP-9	-178.0	-112.5	166.9	176.9	-91.5	-2.1	179.3	30.9	-36.9	28.2	-9.0	$\varepsilon''\delta_R$
tctAAP-10	-175.5	-67.7	-40.8	-13.2	-62.6	-20.6	-179.5	30.6	-36.0	27.1	-8.1	$\alpha_R\delta_R$
cttAAP-11	2.9	-77.6	-29.6	-177.5	-94.4	-1.3	177.8	31.6	-36.0	26.3	-6.5	$\delta_R\delta_R$
tctAAP-12	177.0	122.9	-170.9	24.0	-111.2	13.3	-180.0	35.3	-37.9	25.7	-3.4	$\varepsilon\delta_R$
cctAAP-13	15.5	-136.7	36.0	20.3	-99.4	-14.4	-179.8	32.3	-38.5	29.5	-9.5	$?\delta_R$
tttAAP-14	171.6	134.5	-44.4	-175.9	-83.7	146.1	175.8	24.8	-35.0	31.3	-16.4	$\delta_L\beta_P$
cctAAP-15	16.0	-136.6	32.7	12.2	-92.8	169.7	175.9	35.2	-39.2	27.9	-5.9	$?\varepsilon''$
cttAAP-16	-6.3	-93.8	165.6	178.3	-89.9	-4.7	178.7	30.0	-36.7	28.8	-10.3	$\varepsilon''\delta_R$
cctAAP-17	5.1	-129.2	160.5	-15.4	-69.1	-18.8	178.7	32.4	-35.7	24.6	-4.2	$\beta_S\delta_R$
ttcAAP-18	-178.5	-64.7	-43.2	-177.3	-86.4	158.8	-1.7	30.0	-35.5	27.0	-8.2	$\alpha_R\beta_P$
cctAAP-19	7.5	-73.1	-32.8	-15.6	-74.2	-8.4	-178.3	34.7	-34.2	20.1	2.1	$\delta_R\delta_R$
cccAAP-20	-8.3	139.1	-41.6	-24.9	-64.1	166.9	-3.4	35.5	-34.2	19.4	3.4	$\delta_L\varepsilon''$
tccAAP-21	-167.2	-133.0	35.8	15.4	-94.5	-17.8	6.3	32.4	-37.8	28.2	-8.0	$?\delta_R$
cccAAP-22	-10.4	139.8	-48.1	-12.0	-70.3	-18.5	0.6	31.7	-35.1	24.6	-4.7	$\delta_L\delta_R$

Table S7. Dihedral angle values (in degrees) of all the optimized structures of Ac-azaAsn-Pro-NHMe at the B3LYP-D3/6-311++G(d,p) level of theory in the gas phase.

	ω_0	φ_1	ψ_1	ω_1	φ_2	ψ_2	ω_2	χ_1	χ_2	χ_3	χ_4	χ_1'	χ_2'	Conformer
tttANP-1	-173.6	-60.3	-31.6	178.1	-103.2	17.6	174.8	35.6	-36.4	23.2	-0.7	64.3	171.2	$\alpha_R\delta_R(g^+)$
tctANP-2	-173.0	-127.0	30.4	22.8	-100.4	-13.5	176.2	31.6	-39.5	31.9	-12.5	71.6	157.6	$?\delta_R(g^+)$
tttANP-3	-175.0	-63.6	-41.0	173.7	-87.8	74.5	-165.8	30.1	-36.6	28.5	-9.9	64.1	173.5	$\alpha_R\gamma'(g^+)$
tttANP-4	-177.1	-60.2	-28.7	177.8	-103.0	17.2	174.8	35.9	-36.2	22.6	0.2	123.7	77.8	$\delta_R\delta_R(s^+)$
ttANP-5	175.2	112.8	-25.5	-173.6	-90.5	77.5	-175.5	30.2	-37.1	29.3	-10.6	118.7	-22.0	$\delta_L\gamma'(s^+)$
cttANP-6	8.6	-122.6	30.1	-176.1	-83.3	73.2	-176.7	35.2	-40.2	29.1	-7.1	73.5	159.8	$?\gamma'(g^+)$
tctANP-7	-167.7	106.3	-163.6	39.2	-116.4	-5.2	171.6	36.3	-40.6	29.1	-6.4	101.8	-15.2	$\varepsilon\delta_R(s^+)$
tttANP-8	-172.9	-59.9	-33.3	179.0	-102.1	18.3	174.5	35.8	-36.6	23.3	-0.6	68.4	27.2	$\delta_R\delta_R(g^+)$
tttANP-9	170.2	-146.2	176.0	177.1	-107.1	24.1	174.1	36.2	-35.6	21.3	1.7	66.3	-122.3	$\beta_S\delta_R(g^+)$
cttANP-10	7.0	-126.9	40.1	-174.1	-81.7	70.6	-177.7	36.6	-40.6	28.3	-5.4	61.9	48.8	$\beta\gamma'(g^+)$
cttANP-11	5.2	77.0	22.5	-171.1	-82.2	68.5	-178.2	37.9	-40.7	27.1	-3.4	-83.9	70.9	$\delta_L\gamma'(g^-)$
tttANP-12	-177.1	128.8	-44.8	141.7	-59.0	155.4	-179.2	24.2	-37.1	35.4	-21.4	177.3	-21.9	$?\beta_P(t)$
tctANP-13	-179.1	177.7	-102.5	-1.2	-62.3	144.6	179.4	28.4	-38.2	32.6	-15.5	68.1	23.4	$\varepsilon\beta_P(g^+)$
tctANP-14	169.8	135.2	-73.4	-15.8	-57.3	136.2	-177.0	30.6	-37.2	28.8	-9.9	167.0	-10.4	$\gamma\beta_P(t)$
tttANP-15	171.0	-74.6	158.4	170.8	-80.0	153.6	-179.9	26.2	-36.7	32.7	-17.1	67.2	101.3	$\beta_P\beta_P(g^+)$
cctANP-16	-4.6	-85.6	4.3	-16.4	-53.1	154.3	-177.5	30.7	-38.6	31.5	-12.7	67.4	15.5	$\delta_R\varepsilon''(g^+)$
cctANP-17	14.4	-120.5	13.9	2.9	-84.8	9.9	175.9	36.0	-39.8	27.9	-5.4	76.8	170.7	$\delta_R\delta_R(g^+)$
ctANP-18	-23.4	-66.6	-11.4	169.6	-123.7	1.7	-178.3	37.7	-35.6	20.2	3.7	69.1	-178.3	$\delta_R\delta_R(g^+)$
tcANP-19	-177.8	-66.5	-37.1	-27.8	-35.1	-55.1	175.2	20.8	-33.2	32.7	-20.8	71.1	18.6	$\alpha_R\alpha_R(g^+)$
ctANP-20	-16.2	-106.7	168.5	179.7	-107.1	17.3	175.0	37.1	-36.7	22.2	1.5	72.4	-84.3	$\varepsilon''\delta_R(g^+)$
ccANP-21	7.7	-71.0	-28.4	-31.2	-70.0	74.8	-177.6	37.2	-32.3	14.5	9.5	68.2	-170.7	$\delta_R\gamma'(g^+)$
ccANP-22	15.1	-134.4	45.0	33.2	-106.0	-12.9	170.7	33.3	-40.6	32.1	-11.5	56.8	47.3	$?\delta_R(g^+)$
cttANP-23	-26.7	-54.3	-27.7	175.5	-116.8	-0.9	-178.3	37.1	-36.2	21.6	2.0	121.2	74.8	$\delta_R\delta_R(s^+)$
tctANP-24	-171.7	-74.3	-37.2	-27.8	-42.5	139.1	-174.4	29.1	-38.0	32.2	-14.5	66.1	168.0	$\alpha_R\beta_P(g^+)$
tctANP-25	-178.7	122.4	-16.5	-2.3	-66.2	-11.3	175.3	31.1	-39.1	31.7	-12.6	100.3	-162.8	$\delta_L\delta_R(s^+)$
cttANP-26	-20.3	-57.3	-30.8	179.8	-112.4	3.5	177.7	36.6	-37.0	23.3	-0.2	70.5	27.5	$\delta_R\delta_R(g^+)$
tttANP-27	174.4	133.3	-52.9	-171.9	-90.5	75.1	-175.5	29.3	-36.3	28.7	-10.6	158.2	155.7	$\gamma\gamma'(t)$
ttcANP-28	-179.6	-70.1	-28.4	179.2	-118.2	74.9	-6.5	36.6	-32.0	15.2	8.1	123.1	76.3	$\delta_R\zeta(s^+)$
tccANP-29	-169.1	-123.5	27.7	18.9	-92.7	-18.0	12.2	31.5	-39.2	31.4	-12.1	72.2	98.7	$\delta_R\delta_R(g^+)$
tttANP-30	-176.3	144.8	-53.2	146.4	-61.0	158.7	-179.2	27.1	-37.4	33.0	-16.8	165.3	153.6	$\gamma\beta_P(t)$
cctANP-31	-18.8	-110.3	163.4	-27.6	-62.9	-11.8	-178.7	33.5	-37.8	27.0	-6.0	71.0	-75.3	$\beta_S\delta_R(g^+)$
cccANP-32	4.5	130.8	-30.2	-23.3	-48.7	166.4	-7.4	31.7	-38.5	30.4	-10.8	128.6	1.0	$\delta_L\varepsilon''(s^+)$
cccANP-33	-20.3	141.5	-42.9	-10.4	-66.2	-17.8	9.9	32.4	-38.3	29.0	-8.9	147.8	123.0	$\delta_L\delta_R(s^+)$

Table S8. Dihedral angle values (in degrees) of all the optimized structures of Ac-azaAsp-Pro-NHMe at the B3LYP-D3/6-311++G(d,p) level of theory in the gas phase.

	ω_0	φ_1	ψ_1	ω_1	φ_2	ψ_2	ω_2	χ_1	χ_2	χ_3	χ_4	χ_1'	χ_2'	Conformer
tttADP-1	-175.7	-59.2	-32.4	178.3	-103.4	18.5	174.5	35.7	-36.4	23.1	-0.5	63.2	178.0	$\alpha_R\delta_R(g^+)$
tttADP-2	-176.8	-62.3	-41.5	173.7	-87.9	75.1	-166.1	30.2	-36.4	28.1	-9.4	63.8	177.5	$\alpha_R\gamma'(g^+)$
tctADP-3	-173.7	-127.3	29.9	24.2	-101.5	-13.9	177.3	31.6	-39.6	32.1	-12.6	67.8	171.2	$?\delta_R(g^+)$
tctADP-4	-166.9	110.9	-163.6	24.9	-104.1	151.8	-177.5	35.1	-38.3	26.4	-4.2	92.8	0.0	$\varepsilon\beta_S(s^+)$
cttADP-5	6.4	-123.3	32.4	-175.9	-82.6	73.4	-177.0	35.6	-40.2	28.7	-6.6	70.1	170.2	$\beta\gamma'(g^+)$
tttADP-6	-177.0	-60.1	-29.1	178.5	-102.0	17.0	174.9	36.2	-36.2	22.2	0.8	132.8	66.3	$\delta_R\delta_R(s^+)$
tttADP-7	177.2	113.1	-23.5	-174.4	-90.4	78.6	-175.6	30.0	-37.1	29.5	-11.0	117.5	-29.4	$\delta_L\gamma'(s^+)$
tttADP-8	169.1	-134.2	178.9	179.3	-108.6	26.7	173.4	36.1	-35.3	21.0	2.0	80.5	-160.8	$\varepsilon''\delta_R(g^+)$
tttADP-9	-176.3	133.7	-45.8	142.0	-61.1	157.8	-179.4	25.7	-37.3	34.3	-19.3	118.2	-34.6	$\delta_L\beta_P(s^+)$
cttADP-10	-25.3	-62.3	-16.1	170.7	-122.9	1.1	-178.4	37.6	-35.6	20.3	3.6	68.7	-175.8	$\delta_R\delta_R(g^+)$
cttADP-11	6.2	-124.4	38.2	-173.8	-82.3	72.1	-178.0	36.5	-40.4	28.1	-5.3	71.7	59.0	$?\gamma'(g^+)$
cctADP-12	12.9	-119.3	12.8	3.2	-84.3	9.1	177.6	35.5	-39.8	28.4	-6.2	74.3	178.9	$\delta_R\delta_R(g^+)$
tctADP-13	-168.3	118.8	-167.8	36.8	-120.1	1.5	169.9	38.4	-40.1	26.3	-2.1	105.0	-21.0	$\varepsilon\delta_R(s^+)$
tttADP-14	175.4	131.5	-48.7	-172.0	-90.9	75.7	-175.5	29.2	-36.2	28.7	-10.7	147.5	174.6	$\delta_L\gamma'(s^+)$
cctADP-15	5.1	-67.6	-31.4	-25.6	-71.0	69.1	-178.6	37.3	-35.0	18.7	5.2	71.9	-171.4	$\alpha_R\gamma'(g^+)$
tctADP-16	-179.3	120.8	-15.0	-3.9	-65.8	-12.2	174.9	31.1	-39.0	31.4	-12.3	104.9	-169.5	$\delta_L\delta_R(s^+)$
tctADP-17	-172.8	-75.2	-37.0	-27.2	-42.1	140.5	-176.2	28.8	-38.1	32.6	-15.1	64.5	176.2	$\alpha_R\beta_P(g^+)$
cctADP-18	15.2	-133.9	45.4	31.4	-103.8	-14.1	171.2	33.3	-40.6	31.9	-11.3	63.9	58.6	$?\delta_R(g^+)$
cctADP-19	-9.9	-65.5	-20.7	-28.6	-38.7	153.0	177.9	27.8	-38.4	34.3	-17.6	68.9	33.5	$\delta_R\varepsilon''(g^+)$
tctADP-20	-178.9	-66.5	-38.0	-27.7	-33.8	-60.0	175.0	20.3	-33.0	32.8	-21.2	76.4	13.0	$\alpha_R\alpha_R(g^+)$
tttADP-21	-175.9	143.9	-53.2	145.9	-60.2	157.3	-179.0	26.9	-37.4	33.1	-17.1	158.7	173.3	$\delta_L\beta_P(t)$
tctADP-22	170.5	134.4	-68.4	-20.8	-49.2	150.7	-176.3	30.8	-38.0	30.2	-11.2	165.4	-9.8	$?\beta_P(t)$
cttADP-23	-21.9	-59.1	-26.1	178.6	-113.7	-0.1	179.4	36.9	-37.1	23.1	0.3	128.3	66.3	$\delta_R\delta_R(s^+)$
ttcADP-24	179.5	-67.5	-29.7	179.3	-116.8	77.0	-7.0	36.4	-31.3	14.2	9.1	133.2	65.8	$\delta_R\zeta(s^+)$
tccADP-25	-170.6	-121.0	26.8	18.4	-91.6	-17.2	7.3	31.8	-39.0	30.7	-11.1	80.1	64.2	$\delta_R\delta_R(g^+)$
cttADP-26	-19.1	-114.4	174.6	177.8	-110.3	15.8	175.1	37.2	-36.5	21.7	1.9	69.2	-66.1	$\varepsilon''\delta_R(g^+)$
cttADP-27	-33.0	-47.0	-36.8	177.2	-112.9	-2.0	-179.3	37.1	-36.2	21.5	2.0	76.4	26.2	$\alpha_R\delta_R(g^+)$
cccADP-28	6.9	130.9	-29.7	-25.2	-44.9	167.4	-8.0	30.6	-38.6	31.7	-12.9	121.8	8.1	$\delta_L\varepsilon''(s^+)$
cctADP-29	-29.9	-114.0	164.1	-32.6	-48.4	-25.4	177.8	30.9	-37.6	29.2	-10.2	57.3	-45.1	$\varepsilon''\delta_R(g^+)$
cccADP-30	-16.6	135.8	-27.8	-10.7	-65.2	-19.0	7.4	32.1	-38.0	28.8	-9.0	127.9	67.6	$\delta_L\delta_R(s^+)$

Table S9. Dihedral angle values (in degrees) of all the optimized structures of Ac-azaAla-Pro-NHMe at the B3LYP-D3/6-311++G(d,p) level of theory in the gas phase.

	ω_0	φ_1	ψ_1	ω_1	φ_2	ψ_2	ω_2	χ_1	χ_2	χ_3	χ_4	Conformer
tttAAP-1	-179.6	-55.4	-35.6	179.7	-103.8	20.2	174.1	35.6	-36.4	23.2	-0.7	$\alpha_R\delta_R$
tttAAP-2	177.5	-56.3	-43.6	175.0	-88.8	75.2	-166.0	30.5	-35.8	26.9	-7.9	$\alpha_R\gamma'$
tttAAP-3	172.1	126.0	-37.7	-172.2	-91.4	75.8	-175.2	29.6	-36.2	28.3	-10.0	$\delta_L\gamma'$
tttAAP-4	171.0	-123.9	167.8	179.1	-88.1	77.0	-174.8	25.1	-37.2	34.4	-19.4	$\beta_s\gamma'$
tctAAP-5	170.3	137.4	-63.5	-20.4	-66.7	109.2	-176.5	33.5	-33.9	20.7	0.7	$\gamma\varepsilon''$
cttAAP-6	-7.3	127.4	-32.9	-173.2	-89.0	75.6	-175.7	30.9	-37.9	29.8	-10.7	$\delta_L\gamma'$
cttAAP-7	7.2	-128.6	39.3	-175.2	-81.1	71.8	-177.3	36.4	-40.4	28.2	-5.4	$?\gamma'$
tctAAP-8	-173.2	-124.5	26.4	28.9	-104.5	-14.8	179.0	31.2	-39.8	32.9	-13.7	$?\delta_R$
tttAAP-9	166.6	-77.0	156.7	175.1	-124.3	12.7	174.8	37.2	-36.8	22.8	0.6	$\beta_P\beta$
tctAAP-10	179.7	-64.3	-35.9	-16.1	-44.0	-46.5	174.3	20.9	-34.1	33.9	-21.9	$\alpha_R\alpha_R$
cttAAP-11	-28.4	-50.7	-33.8	177.5	-115.5	0.5	179.5	36.9	-36.2	21.8	1.6	$\alpha_R\delta_R$
tctAAP-12	-171.2	128.6	-172.0	32.5	-118.6	-4.7	178.2	36.7	-39.8	27.7	-4.7	$\varepsilon\delta_R$
cctAAP-13	16.6	-137.2	45.6	33.8	-107.0	-13.9	170.8	32.5	-40.4	32.7	-12.6	$\beta\delta_R$
tttAAP-14	-178.6	139.3	-45.9	142.8	-59.6	155.5	-179.0	25.9	-37.4	34.2	-18.9	$\delta_L\beta_P$
cctAAP-15	5.4	-126.7	30.4	7.5	-83.5	161.8	-172.7	34.5	-39.2	28.6	-6.9	$?\beta_P$
cttAAP-16	-14.5	-98.3	166.9	-176.4	-107.5	10.8	173.9	34.9	-38.6	27.4	-5.6	$\varepsilon''\delta_R$
cctAAP-17	-15.9	-110.3	163.9	-29.8	-62.0	-12.9	179.7	35.1	-37.2	24.5	-2.2	$\varepsilon''\delta_R$
ttcAAP-18	176.6	-63.2	-34.6	-180.0	-117.4	78.3	-7.6	35.8	-31.3	14.9	8.0	$\alpha_R\zeta$
cctAAP-19	5.4	-59.1	-42.2	-13.4	-72.8	55.7	-177.8	36.5	-37.8	23.9	-0.8	$\alpha_R\gamma'$
cccAAP-20	-10.7	125.9	-18.0	-4.7	-67.9	161.7	-7.0	34.4	-38.3	27.2	-5.6	$\delta_L\beta_P$
tccAAP-21	-171.8	-124.0	26.6	23.5	-97.5	-16.0	3.1	31.5	-38.9	31.2	-11.8	$?\delta_R$
cccAAP-22	-20.5	143.2	-50.1	-12.4	-66.6	-16.8	2.9	31.9	-37.5	28.2	-8.3	$\delta_L\delta_R$

Table S10. Dihedral angle values (in degrees) of all the optimized structures of Ac-azaAsn-Pro-NHMe at the SMD/B3LYP-D3/6-311++G(d,p) level of theory in water.

	ω_0	φ_1	ψ_1	ω_1	φ_2	ψ_2	ω_2	χ_1	χ_2	χ_3	χ_4	χ_1'	χ_2'	Conformer
tttANP-1	-178.8	-57.9	-36.7	-180.0	-95.3	6.7	177.0	34.7	-36.8	24.5	-2.6	60.4	-159.6	$\alpha_R\delta_R(g^+)$
tctANP-2	-169.7	-130.4	34.2	20.1	-98.0	-12.8	175.3	33.1	-39.3	30.1	-9.6	72.3	169.9	$?\delta_R(g^+)$
tttANP-3	-179.1	-63.4	-38.2	-174.2	-91.9	70.1	-175.1	31.6	-36.4	26.7	-7.1	59.4	-153.5	$\alpha_R\gamma'(g^+)$
tttANP-4	179.3	-61.4	-31.5	179.9	-94.7	5.5	177.4	34.9	-36.1	23.3	-1.1	125.3	57.8	$\delta_R\delta_R(s^+)(\beta I)$
ttANP-5	179.1	104.2	-9.6	-173.9	-89.5	70.6	-176.5	31.7	-39.0	30.6	-11.1	120.7	-30.4	$\delta_L\gamma'(s^+)$
cttANP-6	5.7	-122.5	30.9	-174.8	-83.5	59.2	-179.9	37.0	-41.6	29.5	-6.5	75.7	167.8	$?\gamma'(g^+)$
tctANP-7	-173.0	91.6	-165.4	26.6	-108.3	-15.7	179.1	34.8	-39.2	28.3	-6.5	84.4	1.3	$\varepsilon\delta_R(g^+)$
tttANP-8	178.8	-58.6	-36.4	-179.9	-96.6	7.9	176.9	34.9	-36.3	23.5	-1.4	73.8	20.7	$\alpha_R\delta_R(g^+)$
tttANP-9	-179.2	-120.1	173.4	178.0	-96.6	13.6	175.1	34.4	-37.5	26.0	-4.4	74.6	-150.5	$\varepsilon''\delta_R(g^+)$
cttANP-10	4.5	-120.0	32.0	-172.8	-83.4	57.1	179.6	37.4	-42.0	29.6	-6.3	67.7	49.1	$\delta_R\gamma'(g^+)$
cttANP-11	-0.4	80.5	22.8	-169.1	-82.8	54.1	178.5	37.8	-42.8	30.6	-7.1	86.2	62.4	$\delta_L\beta(g^+)$
tttANP-12	-176.0	65.4	23.5	-177.5	-61.5	148.5	176.5	33.3	-38.4	28.3	-7.5	94.6	-14.6	$\delta_L\beta_P(s^+)$
tctANP-13	174.7	128.0	-67.5	-20.2	-58.0	148.0	-177.6	31.7	-36.1	26.2	-6.4	85.4	14.9	$\gamma\beta_P(s^+)$
tctANP-14	178.7	132.9	-30.0	-22.9	-51.2	161.4	-176.3	33.1	-37.8	27.8	-7.2	132.6	-13.9	$\delta_L\beta_P(s^+)$
tttANP-15	173.2	-81.8	159.4	170.1	-71.3	149.9	175.8	25.8	-36.9	33.2	-17.8	62.4	85.0	$\beta_P\beta_P(g^+)$
cctANP-16	7.5	-96.2	-3.8	-15.5	-58.4	174.3	-179.4	34.8	-38.6	27.3	-5.5	92.6	-0.8	$\delta_R\varepsilon''(s^+)$
cctANP-17	12.4	-129.9	27.8	12.3	-88.3	-12.9	173.7	32.9	-38.9	29.5	-9.2	73.7	171.5	$?\delta_R(g^+)$
ctANP-18	-1.5	-89.9	-11.8	175.3	-99.7	-0.1	178.8	34.7	-36.8	24.6	-2.7	74.5	-169.8	$\delta_R\delta_R(g^+)$
tcANP-19	179.5	-64.0	-38.9	-14.6	-54.2	-32.0	-179.7	28.3	-36.1	29.6	-12.3	73.2	20.9	$\alpha_R\alpha_R(g^+)$
ctANP-20	-10.6	-90.5	169.2	177.4	-95.1	13.2	175.0	33.2	-38.2	28.1	-7.4	74.1	-144.4	$\varepsilon''\delta_R(g^+)$
ccANP-21	5.4	-80.2	-26.4	-40.8	-68.1	84.9	-174.2	31.8	-15.9	-6.3	27.8	62.9	-163.8	$\delta_R\gamma'(g^+)$
ccANP-22	11.7	-132.1	34.1	21.7	-96.0	-14.9	173.8	32.9	-39.5	30.5	-10.2	63.8	52.8	$\beta\delta_R(g^+)$
cttANP-23	-3.2	-79.5	-20.9	-178.5	-94.9	-1.3	179.0	33.9	-37.1	25.8	-4.5	113.1	55.6	$\delta_R\delta_R(s^+)$
tctANP-24	-178.2	-64.9	-38.4	-22.8	-50.1	147.5	-179.3	30.2	-36.7	28.9	-10.3	62.6	-159.1	$\alpha_R\beta_P(g^+)$
tctANP-25	176.6	126.3	-25.5	-11.0	-63.0	-15.2	173.3	32.8	-37.3	27.1	-6.6	134.3	159.1	$\delta_L\delta_R(s^+)$
cttANP-26	-3.2	-76.4	-25.1	177.1	-98.0	1.3	178.2	34.2	-36.3	24.2	-2.6	85.1	12.8	$\delta_R\delta_R(g^+)$
tttANP-27	176.7	123.9	-32.9	-172.1	-92.5	72.4	-176.0	30.3	-37.1	29.0	-10.4	144.9	153.8	$\delta_L\gamma'(s^+)$
ttcANP-28	178.3	-74.4	-24.1	-179.0	-119.1	68.8	0.3	37.5	-33.4	16.5	7.3	117.9	53.9	$\delta_R\zeta(s^+)$
tccANP-29	-167.8	-127.6	33.0	16.5	-92.0	-18.4	7.3	32.5	-38.5	29.3	-9.1	66.3	44.2	$\beta\delta_R(g^+)$
tttANP-30	176.6	126.5	-39.3	-175.8	-89.7	147.5	175.5	30.4	-35.5	26.5	-7.5	149.6	153.0	$\delta_L\beta_S(s^+)$
cctANP-31	3.0	-117.0	163.5	-27.8	-62.4	-18.7	-178.9	34.3	-35.7	22.8	-1.0	60.9	-160.7	$\beta_S\delta_R(g^+)$
cccANP-32	-4.6	138.1	-41.0	-23.8	-55.6	168.1	-9.3	34.7	-37.2	25.2	-3.2	143.2	-10.8	$\delta_L\varepsilon''(s^+)$
cccANP-33	-8.4	133.4	-37.3	-12.5	-67.6	-18.0	0.1	32.4	-36.4	25.9	-5.6	147.1	151.4	$\delta_L\delta_R(s^+)$

Table S11. Dihedral angle values (in degrees) of all the optimized structures of Ac-azaAsp-Pro-NHMe (**ADP**) at the SMD/B3LYP-D3/6-311++G(d,p) level of theory in water.

	ω_0	φ_1	ψ_1	ω_1	φ_2	ψ_2	ω_2	χ_1	χ_2	χ_3	χ_4	χ_1'	χ_2'	Conformer
tttADP-1	-179.3	-57.8	-36.7	180.0	-94.4	5.9	176.9	34.6	-36.7	24.4	-2.5	64.9	-172.0	$\alpha_R\delta_R(g^+)$
tttADP-2	-179.1	-64.0	-37.5	-172.9	-92.3	66.7	-176.6	32.1	-36.9	26.9	-7.0	65.9	-172.1	$\alpha_R\gamma'(g^+)$
tctADP-3	-170.1	-130.4	34.8	19.8	-97.9	-13.0	175.5	33.2	-39.3	29.8	-9.2	71.4	174.2	$?\delta_R(g^+)$
tctADP-4	-172.5	106.6	-161.8	26.0	-104.1	151.9	-178.0	33.8	-38.9	28.7	-7.6	87.5	-0.8	$\varepsilon\beta_S(g^+)$
cttADP-5	4.7	-122.7	31.9	-174.6	-83.3	58.8	179.9	37.1	-41.8	29.6	-6.6	74.0	174.3	$?\gamma'(g^+)$
tttADP-6	-179.9	-62.2	-30.0	179.5	-92.2	3.7	177.7	34.9	-36.1	23.1	-1.0	136.4	59.6	$\delta_R\delta_R(s^+)$
tttADP-7	-176.7	77.2	24.2	-172.5	-82.3	58.1	179.4	37.1	-42.6	30.8	-7.8	104.8	-31.1	$\delta_L\alpha_R(s^+)$
tttADP-8	172.0	-88.4	177.8	-177.7	-94.2	5.0	176.7	35.0	-36.3	23.5	-1.4	88.4	-179.9	$\varepsilon''\delta_R(g^+)$
tttADP-9	-174.8	72.0	23.3	-177.6	-61.7	148.6	176.5	33.2	-38.3	28.2	-7.6	102.8	-28.9	$\delta_L\beta_P(s^+)$
cttADP-10	-2.6	-82.9	-19.0	177.5	-100.8	0.5	178.7	34.8	-36.6	24.1	-2.1	72.6	-174.4	$\delta_R\delta_R(g^+)$
cttADP-11	4.6	-126.4	42.6	-170.9	-83.1	56.2	178.9	37.5	-42.1	29.7	-6.3	71.9	56.4	$?\gamma'(g^+)$
cctADP-12	12.0	-129.5	28.1	11.8	-88.0	-12.6	174.0	33.0	-38.9	29.3	-8.8	72.9	175.8	$?\delta_R(g^+)$
tctADP-13	-173.1	109.0	-164.6	28.9	-108.9	-13.1	177.1	35.2	-39.5	28.4	-6.4	93.2	-9.0	$\varepsilon\delta_R(s^+)$
tttADP-14	177.5	121.8	-29.0	-172.5	-92.4	72.1	-176.2	30.4	-37.3	29.2	-10.5	137.4	165.9	$\delta_L\gamma'(s^+)$
cctADP-15	4.8	-79.1	-27.5	-39.3	-68.5	87.0	-173.8	32.0	-16.9	-4.9	26.4	67.5	-172.6	$\delta_R\gamma'(g^+)$
tctADP-16	176.2	123.8	-20.0	-13.5	-62.0	-16.4	173.6	32.7	-37.3	27.1	-6.7	120.0	-179.4	$\delta_L\delta_R(s^+)$
tctADP-17	-178.9	-64.4	-39.0	-22.9	-49.7	147.6	-179.0	30.0	-36.8	29.1	-10.7	67.3	-172.3	$\alpha_R\beta_P(g^+)$
cctADP-18	10.5	-134.5	40.7	19.2	-94.5	-15.9	174.3	33.3	-39.1	29.3	-8.5	70.5	58.4	$?\delta_R(g^+)$
cctADP-19	4.2	-86.9	-16.5	-22.9	-53.0	177.4	-179.2	34.4	-38.8	28.1	-6.7	79.7	18.6	$\delta_R\varepsilon''(g^+)$
tctADP-20	178.4	-61.5	-46.6	-22.3	-40.6	-57.7	176.0	23.8	-33.4	29.6	-15.4	70.3	19.2	$\alpha_R\alpha_R(g^+)$
tttADP-21	177.4	126.1	-39.0	-175.5	-89.4	147.6	175.5	30.4	-35.5	26.5	-7.5	146.8	164.4	$\delta_L\beta_P(s^+)$
tctADP-22	176.3	129.4	-28.9	-26.2	-51.0	175.3	-176.1	35.2	-38.5	26.8	-4.8	125.3	-7.1	$\delta_L\beta_P(s^+)$
cttADP-23	4.2	-126.1	38.9	-175.0	-67.9	-23.7	-178.8	32.3	-38.9	29.7	-9.6	72.9	57.2	$?\delta_R(g^+)$
ttcADP-24	178.4	-72.9	-24.7	-178.3	-118.1	68.9	0.7	37.7	-33.3	16.0	8.0	130.8	58.9	$\delta_R\zeta(s^+)$
tccADP-25	-168.6	-126.9	35.8	15.0	-90.8	-18.4	7.4	32.9	-38.7	29.0	-8.6	76.9	56.4	$?\delta_R(g^+)$
cttADP-26	-10.8	-89.1	168.5	177.2	-97.8	3.6	177.9	33.7	-37.2	26.2	-5.0	74.1	-179.5	$\varepsilon''\delta_R(g^+)$
cttADP-27	-3.2	-72.6	-30.3	179.4	-96.4	1.2	178.2	34.1	-36.4	24.3	-2.8	80.1	18.9	$\delta_R\delta_R(g^+)(\beta I)$
cccADP-28	-3.5	138.6	-41.7	-24.6	-51.1	169.0	-9.6	33.5	-37.9	27.4	-6.4	142.7	-10.6	$\delta_L\varepsilon''(s^+)$
cctADP-29	2.7	-117.8	162.2	-27.1	-63.4	-17.8	-179.0	34.3	-35.8	22.9	-1.2	64.6	-174.9	$\beta_S\delta_R(g^+)$
cccADP-30	-8.3	90.7	26.5	20.3	-92.2	-23.9	3.7	32.4	-37.1	27.0	-6.7	94.3	53.7	$\delta_L\delta_R(s^+)$

Table S12. Dihedral angle values (in degrees) of all the optimized structures of Ac-azaAla-Pro-NHMe at the SMD/B3LYP-D3/6-311++G(d,p) level of theory in water.

	ω_0	φ_1	ψ_1	ω_1	φ_2	ψ_2	ω_2	χ_1	χ_2	χ_3	χ_4	Conformer
tttAAP-1	179.2	-56.1	-37.4	-178.8	-94.5	6.2	176.9	34.5	-36.8	24.6	-2.8	$\alpha_R\delta_R(\beta I)$
tttAAP-2	179.3	-62.0	-38.4	-172.5	-91.4	69.3	-175.6	31.5	-36.6	27.1	-7.5	$\alpha_R\gamma'$
tttAAP-3	175.6	128.3	-39.2	-172.7	-93.5	70.8	-175.9	30.8	-36.7	28.0	-8.9	$\delta_L\gamma'$
tttAAP-4	173.6	-79.3	163.7	-176.6	-88.2	73.0	-175.8	28.9	-38.1	32.0	-14.5	$\beta_P\gamma'$
tctAAP-5	171.7	135.7	-48.0	-27.6	-65.3	115.4	-176.2	32.7	-26.8	10.1	11.3	$?\beta_S$
cttAAP-6	-5.3	128.4	-33.4	-172.7	-91.8	69.9	-176.4	31.2	-37.9	29.4	-10.2	$\delta_L\gamma'$
cttAAP-7	4.9	-125.5	35.2	-174.3	-82.8	57.6	179.7	37.2	-41.9	29.8	-6.6	$\beta\gamma'$
tctAAP-8	-170.3	-131.5	36.1	20.0	-98.7	-13.2	175.8	33.2	-39.2	30.0	-9.4	$?\delta_R$
tttAAP-9	173.4	-80.4	164.5	176.6	-85.8	-6.5	179.6	29.8	-37.9	31.0	-12.7	$\beta_P\delta_R$
tctAAP-10	-178.9	-64.6	-38.1	-10.6	-58.7	-26.7	-178.7	29.6	-36.7	29.2	-11.0	$\alpha_R\delta_R$
cttAAP-11	-2.4	-70.5	-31.1	-178.6	-96.5	1.4	178.4	34.0	-36.6	25.0	-3.5	$\delta_R\delta_R$
tctAAP-12	178.1	127.1	-174.4	21.5	-106.6	-7.5	177.4	36.0	-39.4	27.4	-4.8	$\varepsilon\delta_R$
cctAAP-13	14.5	-136.8	37.8	21.8	-97.4	-15.2	174.2	32.4	-39.3	30.7	-10.7	$?\delta_R$
tttAAP-14	176.3	128.6	-40.8	-175.3	-88.7	145.0	176.0	29.6	-35.5	27.4	-9.0	$\delta_L\beta_S$
cctAAP-15	15.5	-139.6	39.0	22.8	-96.9	153.5	-178.9	31.8	-39.3	31.5	-11.9	$?\beta_P$
cttAAP-16	-6.8	-92.4	162.6	177.7	-91.7	-0.5	179.1	31.8	-38.2	29.6	-9.9	$\varepsilon''\delta_R$
cctAAP-17	5.7	-126.3	159.8	-19.3	-66.0	-19.3	-178.3	32.9	-36.3	25.1	-4.3	$\beta_S\delta_R$
ttcAAP-18	178.0	-67.1	-32.7	-177.7	-118.6	72.9	0.0	36.9	-31.8	14.6	8.8	$\alpha_R\zeta$
cctAAP-19	2.4	-68.6	-33.7	-13.2	-57.4	-28.3	-179.1	28.7	-36.6	29.9	-12.3	$\alpha_R\delta_R$
cccAAP-20	-8.8	130.5	-23.0	-12.8	-63.6	163.9	-4.4	34.7	-37.1	25.0	-3.1	$\delta_L\beta_P$
tccAAP-21	-168.2	-130.6	36.2	14.8	-92.5	-18.4	7.0	32.5	-38.5	29.3	-9.1	$?\delta_R$
cccAAP-22	-9.7	136.3	-43.2	-11.4	-67.8	-16.7	0.4	32.5	-36.7	26.4	-6.0	$\delta_L\delta_R$

Table S13: NBO orbital interactions, E(2) value, corresponding to hydrogen bond for the model **ANP** at the B3LYP/ method.

	Donor ⟶ Acceptor	cycle	E(2) (Kcal/mol)	ΣE_{HB} (Kcal/mol)
**tttANP-1**	$\pi_{CO} \rightarrow \sigma^*_{NH}$<br>$n_O \rightarrow \sigma^*_{NH}$<br>$n'_O \rightarrow \sigma^*_{NH}$	C_{10}	1.43<br>1.98<br>0.81	4.22
	$n_O \rightarrow \sigma^*_{NH}$<br>$n'_O \rightarrow \sigma^*_{NH}$	C_6	0.53<br>1.60	2.13
**tctANP-2**	$n_O \rightarrow \sigma^*_{NH}$<br>$n'_O \rightarrow \sigma^*_{NH}$	C_6	0.53<br>1.57	2.10
	$n_N \rightarrow \sigma^*_{NH}$	C_5	1.15	1.15
**tttANP-3**	$\pi_{CO} \rightarrow \sigma^*_{NH}$<br>$n_O \rightarrow \sigma^*_{NH}$<br>$n'_O \rightarrow \sigma^*_{NH}$	C_7	1.02<br>1.11<br>1.29	3.42
	$n'_O \rightarrow \sigma^*_{NH}$	C_6	1.00	1.00
**tttANP-4**	$\pi_{CO} \rightarrow \sigma^*_{NH}$<br>$n_O \rightarrow \sigma^*_{NH}$<br>$n'_O \rightarrow \sigma^*_{NH}$	C_{10}	1.27<br>1.64<br>0.64	3.55
	$\pi_{CO} \rightarrow \sigma^*_{NH}$<br>$n_O \rightarrow \sigma^*_{NH}$<br>$n'_O \rightarrow \sigma^*_{NH}$	C_7	1.09<br>1.32<br>1.39	3.80
	$n_N \rightarrow \sigma^*_{NH}$	C_5	0.51	0.51
**tttANP-5**	$n_O \rightarrow \sigma^*_{NH}$<br>$n'_O \rightarrow \sigma^*_{NH}$	C_7	1.41<br>1.77	3.18
	$n_O \rightarrow \sigma^*_{NH}$<br>$n'_O \rightarrow \sigma^*_{NH}$	C_8	2.07<br>4.40	6.47
**cttANP-6**	$\pi_{CO} \rightarrow \sigma^*_{NH}$<br>$n_O \rightarrow \sigma^*_{NH}$<br>$n'_O \rightarrow \sigma^*_{NH}$	C_7	0.73<br>3.01<br>3.99	7.73
	$n'_O \rightarrow \sigma^*_{NH}$	C_6	1.01	1.01
**tctANP-7**	$\pi_{CO} \rightarrow \sigma^*_{NH}$<br>$n_O \rightarrow \sigma^*_{NH}$<br>$n'_O \rightarrow \sigma^*_{NH}$	C_8	0.90<br>2.56<br>5.88	9.34
	$n_N \rightarrow \sigma^*_{NH}$	C_5	1.08	1.08
**tttANP-8**	$\pi_{CO} \rightarrow \sigma^*_{NH}$<br>$n_O \rightarrow \sigma^*_{NH}$<br>$n'_O \rightarrow \sigma^*_{NH}$	C_{10}	1.27<br>1.75<br>0.67	3.69
**tttANP-9**	$n_O \rightarrow \sigma^*_{NH}$<br>$n'_O \rightarrow \sigma^*_{NH}$	C_{10}	2.74<br>3.02	5.76
	$n_O \rightarrow \sigma^*_{NH}$<br>$n'_O \rightarrow \sigma^*_{NH}$	C_5	0.69<br>2.65	3.34
**cttANP-10**	$\pi_{CO} \rightarrow \sigma^*_{NH}$<br>$n_O \rightarrow \sigma^*_{NH}$<br>$n'_O \rightarrow \sigma^*_{NH}$	C_7	0.89<br>3.33<br>4.30	8.52
	$\pi_{CO} \rightarrow \sigma^*_{NH}$<br>$n_O \rightarrow \sigma^*_{NH}$<br>$n'_O \rightarrow \sigma^*_{NH}$	C_7	1.22<br>1.33<br>1.88	4.43
**cttANP-11**	$\pi_{CO} \rightarrow \sigma^*_{NH}$<br>$n_O \rightarrow \sigma^*_{NH}$<br>$n'_O \rightarrow \sigma^*_{NH}$	C_7	0.96<br>2.01<br>2.08	5.05
	$\pi_{CO} \rightarrow \sigma^*_{NH}$<br>$n_O \rightarrow \sigma^*_{NH}$<br>$n'_O \rightarrow \sigma^*_{NH}$	C_7	0.94<br>1.92<br>1.91	4.77
**tttANP-12**	$n_O \rightarrow \sigma^*_{NH}$<br>$n'_O \rightarrow \sigma^*_{NH}$	C_8	1.75<br>1.88	3.63
	$n_O \rightarrow \sigma^*_{NH}$<br>$n'_O \rightarrow \sigma^*_{NH}$	C_8	2.67<br>5.90	8.57
**tctANP-13**	$\pi_{CO} \rightarrow \sigma^*_{NH}$	C_8	0.81	3.50

	$n_O \rightarrow \sigma^*_{NH}$<br>$n'_O \rightarrow \sigma^*_{NH}$		0.63<br>2.06	
	$n_O \rightarrow \sigma^*_{NH}$<br>$n'_O \rightarrow \sigma^*_{NH}$	C_{10}	1.94<br>1.14	3.08
**tctANP-14**	$\pi_{CO} \rightarrow \sigma^*_{NH}$<br>$n_O \rightarrow \sigma^*_{NH}$	C_{10}	3.73<br>0.88	4.61
	$n_O \rightarrow \sigma^*_{NH}$<br>$n'_O \rightarrow \sigma^*_{NH}$	C_{10}	3.49<br>1.85	5.34
**tttANP-15**	$\pi_{CO} \rightarrow \sigma^*_{NH}$<br>$n_O \rightarrow \sigma^*_{NH}$<br>$n'_O \rightarrow \sigma^*_{NH}$	C_{10}	1.37<br>3.08<br>1.01	5.46
**cctANP-16**	$\pi_{CO} \rightarrow \sigma^*_{NH}$<br>$n_O \rightarrow \sigma^*_{NH}$	C_{10}	1.95<br>2.27	4.22
**cctANP-17**	$n_O \rightarrow \sigma^*_{NH}$<br>$n'_O \rightarrow \sigma^*_{NH}$	C_{10}	1.00<br>0.94	1.94
**cttANP-18**	$n_N \rightarrow \sigma^*_{NH}$	C_5	1.56	1.56
**tctANP-19**	$\pi_{CO} \rightarrow \sigma^*_{NH}$<br>$n_O \rightarrow \sigma^*_{NH}$	C_{10}	2.02<br>0.60	2.62
	$n'_O \rightarrow \sigma^*_{NH}$	C_{10}	1.01	1.01
**cttANP-20**	$n_O \rightarrow \sigma^*_{NH}$<br>$n'_O \rightarrow \sigma^*_{NH}$	C_{10}	2.56<br>2.94	5.50
**cctANP-21**	$n_O \rightarrow \sigma^*_{NH}$<br>$n'_O \rightarrow \sigma^*_{NH}$	C_{10}	2.25<br>3.43	5.68
**cctANP-22**	$\pi_{CO} \rightarrow \sigma^*_{NH}$<br>$n_O \rightarrow \sigma^*_{NH}$<br>$n'_O \rightarrow \sigma^*_{NH}$	C_7	1.41<br>1.28<br>2.27	4.96
**cttANP-23**	$\pi_{CO} \rightarrow \sigma^*_{NH}$<br>$n_O \rightarrow \sigma^*_{NH}$<br>$n'_O \rightarrow \sigma^*_{NH}$	C_7	0.75<br>1.00<br>0.94	2.69
	$n_N \rightarrow \sigma^*_{NH}$	C_5	1.30	1.30
**tctANP-24**	$n_O \rightarrow \sigma^*_{NH}$<br>$n'_O \rightarrow \sigma^*_{NH}$	C_6	0.58<br>1.83	2.41
**tctANP-25**	$n_O \rightarrow \sigma^*_{NH}$<br>$n'_O \rightarrow \sigma^*_{NH}$	C_{10}	2.78<br>4.16	6.94
**cttANP-26**	$n_N \rightarrow \sigma^*_{NH}$	C_5	1.17	1.17
**tttANP-27**	$\pi_{CO} \rightarrow \sigma^*_{NH}$<br>$n_O \rightarrow \sigma^*_{NH}$<br>$n'_O \rightarrow \sigma^*_{NH}$	C_7	0.84<br>1.33<br>1.86	4.03
**ttcANP-28**	$\pi_{CO} \rightarrow \sigma^*_{NH}$<br>$n_O \rightarrow \sigma^*_{NH}$<br>$n'_O \rightarrow \sigma^*_{NH}$	C_7	1.17<br>1.21<br>1.23	3.61
**tccANP-29**	$n_O \rightarrow \sigma^*_{NH}$<br>$n'_O \rightarrow \sigma^*_{NH}$	C_7	0.75<br>1.39	2.14
**tttANP-30**	$n_O \rightarrow \sigma^*_{NH}$<br>$n'_O \rightarrow \sigma^*_{NH}$	C_8	1.48<br>1.69	3.17
**cctANP-31**	$n'_O \rightarrow \sigma^*_{NH}$	C_5	0.95	0.95
**cccANP-32**	$\pi_{CO} \rightarrow \sigma^*_{NH}$<br>$n_O \rightarrow \sigma^*_{NH}$	C_{10}	1.37<br>1.81	3.18
**cccANP-33**	No interaction			

Table S14: NBO orbital interactions, E(2) values, corresponding to hydrogen bond for ADP with B3LYP method.

	Donor $\rightarrow$ Acceptor	Cycle	E(2) (Kcal/mol)	ΣE_{HB} (Kcal/mol)
**tttADP-1**	$\pi_{CO} \rightarrow \sigma^*_{NH}$<br>$n_O \rightarrow \sigma^*_{NH}$<br>$n'_O \rightarrow \sigma^*_{NH}$	C_{10}	1.37<br>1.70<br>0.64	3.71
	$n'_O \rightarrow \sigma^*_{NH}$	C_6	0.59	0.59
**tttADP-2**	$\pi_{CO} \rightarrow \sigma^*_{NH}$<br>$n_O \rightarrow \sigma^*_{NH}$<br>$n'_O \rightarrow \sigma^*_{NH}$	C_7	0.93<br>1.04<br>1.24	3.21
**tctADP-3**	$n_N \rightarrow \sigma^*_{NH}$	C_5	1.20	1.20
	$n'_O \rightarrow \sigma^*_{NH}$	C_6	0.67	0.67
**tctADP-4**	$\pi_{CO} \rightarrow \sigma^*_{OH}$<br>$n_O \rightarrow \sigma^*_{OH}$<br>$n'_O \rightarrow \sigma^*_{OH}$	C_8	0.84<br>5.24<br>16.51	22.59
**cttADP-5**	$\pi_{CO} \rightarrow \sigma^*_{NH}$<br>$n_O \rightarrow \sigma^*_{NH}$<br>$n'_O \rightarrow \sigma^*_{NH}$	C_7	0.60<br>3.04<br>4.11	7.75
**tttADP-6**	$\pi_{CO} \rightarrow \sigma^*_{NH}$<br>$n_O \rightarrow \sigma^*_{NH}$<br>$n'_O \rightarrow \sigma^*_{NH}$	C_{10}	1.21<br>1.60<br>0.59	3.40
	$\pi_{CO} \rightarrow \sigma^*_{OH}$<br>$n_O \rightarrow \sigma^*_{OH}$<br>$n'_O \rightarrow \sigma^*_{OH}$	C_7	3.44<br>5.05<br>7.18	15.67
**tttADP-7**	$n_O \rightarrow \sigma^*_{OH}$<br>$n'_O \rightarrow \sigma^*_{OH}$	C_8	3.99<br>10.07	14.06
	$n_O \rightarrow \sigma^*_{NH}$<br>$n'_O \rightarrow \sigma^*_{NH}$	C_7	1.24<br>1.58	2.82
**tttADP-8**	$\pi_{CO} \rightarrow \sigma^*_{NH}$<br>$n_O \rightarrow \sigma^*_{NH}$<br>$n'_O \rightarrow \sigma^*_{NH}$	C_{10}	0.68<br>1.59<br>1.07	3.34
	$n_O \rightarrow \sigma^*_{NH}$<br>$n'_O \rightarrow \sigma^*_{NH}$	C_5	0.56<br>2.20	2.76
**tttADP-9**	$n_O \rightarrow \sigma^*_{NH}$<br>$n'_O \rightarrow \sigma^*_{NH}$	C_8	2.46<br>2.69	5.15
	$n_O \rightarrow \sigma^*_{OH}$<br>$n'_O \rightarrow \sigma^*_{OH}$	C_8	5.29<br>14.42	19.71
**cttADP-10**	$n_N \rightarrow \sigma^*_{NH}$	C_5	1.49	1.49
**cttADP-11**	$n_O \rightarrow \sigma^*_{NH}$<br>$n'_O \rightarrow \sigma^*_{NH}$	C_7	3.59<br>2.80	6.39
	$n_O \rightarrow \sigma^*_{OH}$<br>$n'_O \rightarrow \sigma^*_{OH}$	C_7	5.02<br>9.88	14.90
**cctADP-12**	No interaction			
**tctADP-13**	$\pi_{CO} \rightarrow \sigma^*_{OH}$<br>$n_O \rightarrow \sigma^*_{OH}$<br>$n'_O \rightarrow \sigma^*_{OH}$	C_8	0.94<br>4.99<br>14.53	20.46
**tttADP-14**	$\pi_{CO} \rightarrow \sigma^*_{NH}$<br>$n_O \rightarrow \sigma^*_{NH}$<br>$n'_O \rightarrow \sigma^*_{NH}$	C_7	0.72<br>1.27<br>1.82	3.81
**cctADP-15**	$n_O \rightarrow \sigma^*_{NH}$<br>$n'_O \rightarrow \sigma^*_{NH}$	C_{10}	1.18<br>2.40	3.58
**tctADP-16**	$n_O \rightarrow \sigma^*_{NH}$<br>$n'_O \rightarrow \sigma^*_{NH}$	C_{10}	1.97<br>3.11	5.08
**tctADP-17**	$n'_O \rightarrow \sigma^*_{NH}$	C_6	0.80	0.80
**cctADP-18**	$\pi_{CO} \rightarrow \sigma^*_{OH}$<br>$n_O \rightarrow \sigma^*_{OH}$<br>$n'_O \rightarrow \sigma^*_{OH}$	C_7	2.15<br>4.65<br>11.13	17.93
**cctADP-19**	$\pi_{CO} \rightarrow \sigma^*_{OH}$<br>$n_O \rightarrow \sigma^*_{OH}$	C_{10}	3.32<br>4.05	7.37
**tctADP-20**	$\pi_{CO} \rightarrow \sigma^*_{OH}$	C_{10}	2.49	3.27

	$n_O \rightarrow \sigma^*_{OH}$		0.78	
**tttADP-21**	$n_O \rightarrow \sigma^*_{NH}$<br>$n'_O \rightarrow \sigma^*_{NH}$	C_8	1.59<br>1.78	3.37
**tctADP-22**	$\pi_{CO} \rightarrow \sigma^*_{OH}$<br>$n_O \rightarrow \sigma^*_{OH}$	C_{10}	5.98<br>4.22	10.20
	$n_O \rightarrow \sigma^*_{NH}$	C_{10}	1.04	1.04
**cttADP-23**	$\pi_{CO} \rightarrow \sigma^*_{OH}$<br>$n_O \rightarrow \sigma^*_{OH}$<br>$n'_O \rightarrow \sigma^*_{OH}$	C_7	3.66<br>4.26<br>5.24	13.16
	$n_N \rightarrow \sigma^*_{NH}$	C_5	1.11	1.11
**ttcADP-24**	$\pi_{CO} \rightarrow \sigma^*_{OH}$<br>$n_O \rightarrow \sigma^*_{OH}$<br>$n'_O \rightarrow \sigma^*_{OH}$	C_7	4.02<br>4.77<br>6.47	15.26
**tccADP-25**	$\sigma_{CO} \rightarrow \sigma^*_{OH}$<br>$\pi_{CO} \rightarrow \sigma^*_{OH}$<br>$n_O \rightarrow \sigma^*_{OH}$<br>$n'_O \rightarrow \sigma^*_{OH}$	C_7	0.71<br>1.23<br>4.74<br>11.44	18.12
**cttADP-26**	$n'_O \rightarrow \sigma^*_{NH}$	C_5	1.84	1.84
	$n_N \rightarrow \sigma^*_{OH}$	C_6	4.49	4.49
**cttADP-27**	$n_N \rightarrow \sigma^*_{OH}$	C_5	2.49	2.49
	$n_N \rightarrow \sigma^*_{NH}$	C_5	1.04	1.04
**cccADP-28**	$\pi_{CO} \rightarrow \sigma^*_{OH}$<br>$n_O \rightarrow \sigma^*_{OH}$<br>$n'_O \rightarrow \sigma^*_{OH}$	C_{10}	1.82<br>2.72<br>0.51	5.05
**cctADP-29**	$n_N \rightarrow \sigma^*_{OH}$	C_6	9.75	9.75
**cccADP-30**	$\pi_{CO} \rightarrow \sigma^*_{OH}$<br>$n_O \rightarrow \sigma^*_{OH}$<br>$n'_O \rightarrow \sigma^*_{OH}$	C_7	4.25<br>5.59<br>6.92	16.76

Table S15. NBO orbital interactions, E(2) values, corresponding to hydrogen bond for AAP at the B3LYP/6-311++G(d,p) level of theory.

**Conf.**	**Donor ⟶ Acceptor**	**Cycle**	**E(2) (kcal/mol)**	**ΣE$_{HB}$ (Kcal/mol)**
**tttAAP-1**	$\pi_{CO} \longrightarrow \sigma^*_{NH}$<br>$n_O \longrightarrow \sigma^*_{NH}$<br>$n'_O \longrightarrow \sigma^*_{NH}$	C_{10}	1.41<br>1.68<br>0.53	3.62
**tttAAP-2**	$\pi_{CO} \longrightarrow \sigma^*_{NH}$<br>$n_O \longrightarrow \sigma^*_{NH}$<br>$n'_O \longrightarrow \sigma^*_{NH}$	C_7	1.05<br>1.02<br>1.29	3.36
**tttAAP-3**	$\pi_{CO} \longrightarrow \sigma^*_{NH}$<br>$n_O \longrightarrow \sigma^*_{NH}$<br>$n'_O \longrightarrow \sigma^*_{NH}$	C_7	0.71<br>1.35<br>1.75	3.81
**tttAAP-4**	$\pi_{CO} \longrightarrow \sigma^*_{NH}$<br>$n_O \longrightarrow \sigma^*_{NH}$<br>$n'_O \longrightarrow \sigma^*_{NH}$	C_7	0.88<br>1.09<br>1.29	3.26
	$n'_O \longrightarrow \sigma^*_{NH}$	C_5	1.31	1.31
**tctAAP-5**	$\pi_{CO} \longrightarrow \sigma^*_{NH}$<br>$n_O \longrightarrow \sigma^*_{NH}$<br>$n'_O \longrightarrow \sigma^*_{NH}$	C_{10}	0.64<br>3.70<br>4.34	8.68
**cttAAP-6**	$\pi_{CO} \longrightarrow \sigma^*_{NH}$<br>$n_O \longrightarrow \sigma^*_{NH}$<br>$n'_O \longrightarrow \sigma^*_{NH}$	C_7	0.65<br>1.62<br>1.92	4.19
**cttAAP-7**	$\pi_{CO} \longrightarrow \sigma^*_{NH}$<br>$n_O \longrightarrow \sigma^*_{NH}$<br>$n'_O \longrightarrow \sigma^*_{NH}$	C_7	0.83<br>3.47<br>5.05	9.35
**tctAAP-8**	$n_N \longrightarrow \sigma^*_{NH}$	C_5	1.34	1.34
**tttAAP-9**	$n_N \longrightarrow \sigma^*_{NH}$	C_5	1.34	1.34
	$n'_O \longrightarrow \sigma^*_{NH}$	C_5	1.58	1.58
**tctAAP-10**	No interaction			
**cttAAP-11**	$n_N \longrightarrow \sigma^*_{NH}$	C_5	1.35	1.35
**tctAAP-12**	$n_N \longrightarrow \sigma^*_{NH}$	C_5	1.47	1.47
**cctAAP-13**	No interaction			
**tttAAP-14**	$n_O \longrightarrow \sigma^*_{NH}$<br>$n'_O \longrightarrow \sigma^*_{NH}$	C_8	1.10<br>1.28	2.38
**cctAAP-15**	$\pi_{CO} \longrightarrow \sigma^*_{NH}$<br>$n_O \longrightarrow \sigma^*_{NH}$<br>$n'_O \longrightarrow \sigma^*_{NH}$	C_{10}	1.07<br>0.61<br>0.79	2.47
**cttAAP-16**	$n_N \longrightarrow \sigma^*_{NH}$	C_5	1.05	1.05
**cctAAP-17**	No interaction			
**ttcAAP-18**	No interaction			
**cctAAP-19**	$n_N \longrightarrow \sigma^*_{NH}$	C_7	3.56	3.56
**cccAAP-20**	No interaction			
**tccAAP-21**	No interaction			
**cccAAP-22**	No interaction			

Table S16: NBO orbital interactions, E(2) values, corresponding to hydrogen bond for ANP with B3LYP-D_3 method.

	Donor → Acceptor	Cycle	E(2) (Kcal/mol)	ΣE_{HB} (Kcal/mol)
**tttANP-1**	$\pi_{CO} \rightarrow \sigma^*_{NH}$	C_{10}	1.95	5.39
	$n_O \rightarrow \sigma^*_{NH}$		2.60	
	$n'_O \rightarrow \sigma^*_{NH}$		0.84	
	$n_N \rightarrow \sigma^*_{NH}$	C_5	0.55	0.55
	$n'_O \rightarrow \sigma^*_{NH}$	C_6	1.26	1.26
**tctANP-2**	$n_N \rightarrow \sigma^*_{NH}$	C_5	1.19	1.19
	$n_O \rightarrow \sigma^*_{NH}$	C_6	0.51	1.97
	$n'_O \rightarrow \sigma^*_{NH}$		1.46	
**tttANP-3**	$\pi_{CO} \rightarrow \sigma^*_{NH}$	C_7	1.40	2.73
	$n_O \rightarrow \sigma^*_{NH}$		0.68	
	$n'_O \rightarrow \sigma^*_{NH}$		0.65	
	$n'_O \rightarrow \sigma^*_{NH}$	C_6	0.96	0.96
**tttANP-4**	$\pi_{CO} \rightarrow \sigma^*_{NH}$	C_{10}	1.77	4.68
	$n_O \rightarrow \sigma^*_{NH}$		2.27	
	$n'_O \rightarrow \sigma^*_{NH}$		0.64	
	$\pi_{CO} \rightarrow \sigma^*_{NH}$	C_7	1.18	4.03
	$n_O \rightarrow \sigma^*_{NH}$		1.40	
	$n'_O \rightarrow \sigma^*_{NH}$		1.45	
**tttANP-5**	$n_O \rightarrow \sigma^*_{NH}$	C_8	2.18	9.79
	$n'_O \rightarrow \sigma^*_{NH}$		4.61	
	$n_O \rightarrow \sigma^*_{NH}$	C_7	1.41	3.19
	$n'_O \rightarrow \sigma^*_{NH}$		1.78	
**cttANP-6**	$\pi_{CO} \rightarrow \sigma^*_{NH}$	C_7	0.90	8.14
	$n_O \rightarrow \sigma^*_{NH}$		3.13	
	$n'_O \rightarrow \sigma^*_{NH}$		4.11	
	$n'_O \rightarrow \sigma^*_{NH}$	C_6	0.89	0.89
**tctANP-7**	$\pi_{CO} \rightarrow \sigma^*_{NH}$	C_8	0.71	9.98
	$n_O \rightarrow \sigma^*_{NH}$		2.98	
	$n'_O \rightarrow \sigma^*_{NH}$		6.29	
**tttANP-8**	$\pi_{CO} \rightarrow \sigma^*_{NH}$	C_{10}	1.75	4.94
	$n_O \rightarrow \sigma^*_{NH}$		2.44	
	$n'_O \rightarrow \sigma^*_{NH}$		0.75	
**tttANP-9**	$n_O \rightarrow \sigma^*_{NH}$	C_{10}	3.28	7.59
	$n'_O \rightarrow \sigma^*_{NH}$		4.31	
**cttANP-10**	$\pi_{CO} \rightarrow \sigma^*_{NH}$	C_7	1.28	4.25
	$n_O \rightarrow \sigma^*_{NH}$		1.17	
	$n'_O \rightarrow \sigma^*_{NH}$		1.80	
	$\pi_{CO} \rightarrow \sigma^*_{NH}$	C_7	0.95	8.62
	$n_O \rightarrow \sigma^*_{NH}$		3.34	
	$n'_O \rightarrow \sigma^*_{NH}$		4.33	
**cttANP-11**	$\pi_{CO} \rightarrow \sigma^*_{NH}$	C_7	0.83	9.11
	$n_O \rightarrow \sigma^*_{NH}$		3.69	
	$n'_O \rightarrow \sigma^*_{NH}$		4.59	
	$n_O \rightarrow \sigma^*_{NH}$	C_7	2.48	5.58
	$n'_O \rightarrow \sigma^*_{NH}$		3.10	
**tttANP-12**	$n_O \rightarrow \sigma^*_{NH}$	C_8	2.72	8.86
	$n'_O \rightarrow \sigma^*_{NH}$		6.14	
	$n_O \rightarrow \sigma^*_{NH}$	C_8	3.20	6.25
	$n'_O \rightarrow \sigma^*_{NH}$		3.05	
**tctANP-13**	$\pi_{CO} \rightarrow \sigma^*_{NH}$	C_8	1.06	3.83
	$n_O \rightarrow \sigma^*_{NH}$		0.70	
	$n'_O \rightarrow \sigma^*_{NH}$		2.07	
**tctANP-14**	$\pi_{CO} \rightarrow \sigma^*_{NH}$	C_{10}	0.62	8.65
	$n_O \rightarrow \sigma^*_{NH}$		4.78	
	$n'_O \rightarrow \sigma^*_{NH}$		3.25	

	$\pi_{CO} \rightarrow \sigma^*_{NH}$<br>$n_O \rightarrow \sigma^*_{NH}$	C_{10}	4.11<br>0.81	4.92
**tttANP-15**	$\pi_{CO} \rightarrow \sigma^*_{NH}$<br>$n_O \rightarrow \sigma^*_{NH}$<br>$n'_O \rightarrow \sigma^*_{NH}$	C_{10}	1.93<br>3.61<br>0.86	6.40
	$\sigma_{CO} \rightarrow \sigma^*_{NH}$	C_6	1.09	1.09
**cctANP-16**	$\pi_{CO} \rightarrow \sigma^*_{NH}$<br>$n_O \rightarrow \sigma^*_{NH}$	C_{10}	1.80<br>1.25	3.05
**cctANP-17**	$n_O \rightarrow \sigma^*_{NH}$<br>$n'_O \rightarrow \sigma^*_{NH}$	C_{10}	1.00<br>0.88	1.88
**cttANP-18**	$n_N \rightarrow \sigma^*_{NH}$	C_5	1.22	1.22
**tctANP-19**	$\pi_{CO} \rightarrow \sigma^*_{NH}$<br>$n_O \rightarrow \sigma^*_{NH}$<br>$n'_O \rightarrow \sigma^*_{NH}$	C_{10}	2.34<br>0.69<br>0.54	3.57
**cttANP-20**	$\pi_{CO} \rightarrow \sigma^*_{NH}$<br>$n_O \rightarrow \sigma^*_{NH}$<br>$n'_O \rightarrow \sigma^*_{NH}$	C_{10}	0.78<br>2.73<br>3.26	6.77
	$n'_O \rightarrow \sigma^*_{NH}$	C_5	0.57	0.57
**cctANP-21**	$n_O \rightarrow \sigma^*_{NH}$<br>$n'_O \rightarrow \sigma^*_{NH}$	C_{10}	2.08<br>3.83	5.91
**cctANP-22**	$\pi_{CO} \rightarrow \sigma^*_{NH}$<br>$n_O \rightarrow \sigma^*_{NH}$<br>$n'_O \rightarrow \sigma^*_{NH}$	C_7	1.41<br>1.12<br>2.23	4.76
**cttANP-23**	$\pi_{CO} \rightarrow \sigma^*_{NH}$<br>$n_O \rightarrow \sigma^*_{NH}$<br>$n'_O \rightarrow \sigma^*_{NH}$	C_7	1.31<br>1.03<br>0.96	3.30
	$\sigma_N \rightarrow \sigma^*_{NH}$	C_5	0.92	0.92
**tctANP-24**	$n_O \rightarrow \sigma^*_{NH}$<br>$n'_O \rightarrow \sigma^*_{NH}$	C_6	0.51<br>1.53	3.04
**tctANP-25**	$n_O \rightarrow \sigma^*_{NH}$<br>$n'_O \rightarrow \sigma^*_{NH}$	C_{10}	2.15<br>2.10	4.25
**cttANP-26**	$n_N \rightarrow \sigma^*_{NH}$	C_5	1.28	1.28
**tttANP-27**	$\pi_{CO} \rightarrow \sigma^*_{NH}$<br>$n_O \rightarrow \sigma^*_{NH}$<br>$n'_O \rightarrow \sigma^*_{NH}$	C_7	0.85<br>1.22<br>1.78	3.85
**ttcANP-28**	$\pi_{CO} \rightarrow \sigma^*_{NH}$<br>$n_O \rightarrow \sigma^*_{NH}$<br>$n'_O \rightarrow \sigma^*_{NH}$	C_7	1.30<br>1.32<br>1.35	3.97
**tccANP-29**	$n_O \rightarrow \sigma^*_{NH}$<br>$n'_O \rightarrow \sigma^*_{NH}$	C_7	0.61<br>0.69	1.78
**tttANP-30**	$n_O \rightarrow \sigma^*_{NH}$<br>$n'_O \rightarrow \sigma^*_{NH}$	C_8	2.76<br>2.81	5.57
**cctANP-31**	No interaction			
**cccANP-32**	$\pi_{CO} \rightarrow \sigma^*_{NH}$<br>$n_O \rightarrow \sigma^*_{NH}$	C_{10}	1.25<br>2.27	3.52
**cccANP-33**	No interaction			

Table S17: NBO orbital interactions, E(2) values, corresponding to hydrogen bond for ADP with B3LYP-D_3 method.

	Donor ⟶ Acceptor	Cycle	E(2) (Kcal/mol)	ΣE_{HB} (Kcal/mol)
**tttADP-1**	$\pi_{CO} \rightarrow \sigma^*_{NH}$<br>$n_O \rightarrow \sigma^*_{NH}$<br>$n'_O \rightarrow \sigma^*_{NH}$	C_{10}	1.91<br>2.37<br>0.69	4.97
	$n'_O \rightarrow \sigma^*_{NH}$	C_6	0.54	0.54
	$n_N \rightarrow \sigma^*_{NH}$	C_5	0.51	0.51
**tttADP-2**	$\pi_{CO} \rightarrow \sigma^*_{NH}$<br>$n_O \rightarrow \sigma^*_{NH}$<br>$n'_O \rightarrow \sigma^*_{NH}$	C_7	1.29<br>0.65<br>0.65	2.59
**tctADP-3**	$n_N \rightarrow \sigma^*_{NH}$	C_5	1.22	1.22
**tctADP-4**	$\pi_{CO} \rightarrow \sigma^*_{OH}$<br>$n_O \rightarrow \sigma^*_{OH}$<br>$n'_O \rightarrow \sigma^*_{OH}$	C_8	0.85<br>5.14<br>15.93	21.92
**cttADP-5**	$\pi_{CO} \rightarrow \sigma^*_{NH}$<br>$n_O \rightarrow \sigma^*_{NH}$<br>$n'_O \rightarrow \sigma^*_{NH}$	C_7	0.74<br>3.12<br>4.19	8.05
**tttADP-6**	$\pi_{CO} \rightarrow \sigma^*_{NH}$<br>$n_O \rightarrow \sigma^*_{NH}$<br>$n'_O \rightarrow \sigma^*_{NH}$	C_{10}	1.66<br>2.20<br>0.60	4.46
	$\pi_{CO} \rightarrow \sigma^*_{OH}$<br>$n_O \rightarrow \sigma^*_{OH}$<br>$n'_O \rightarrow \sigma^*_{OH}$	C_7	3.47<br>5.02<br>7.25	15.74
**tttADP-7**	$n_O \rightarrow \sigma^*_{NH}$<br>$n'_O \rightarrow \sigma^*_{NH}$	C_7	1.27<br>1.63	2.90
	$n_O \rightarrow \sigma^*_{OH}$<br>$n'_O \rightarrow \sigma^*_{OH}$	C_8	3.99<br>10.15	14.14
**tttADP-8**	$\pi_{CO} \rightarrow \sigma^*_{NH}$<br>$n_O \rightarrow \sigma^*_{NH}$<br>$n'_O \rightarrow \sigma^*_{NH}$	C_{10}	0.99<br>2.09<br>1.18	4.26
	$n_O \rightarrow \sigma^*_{NH}$<br>$n'_O \rightarrow \sigma^*_{NH}$	C_5	0.52<br>2.04	2.56
**tttADP-9**	$n_O \rightarrow \sigma^*_{NH}$<br>$n'_O \rightarrow \sigma^*_{NH}$	C_8	3.89<br>3.97	7.86
	$n_O \rightarrow \sigma^*_{OH}$<br>$n'_O \rightarrow \sigma^*_{OH}$	C_8	5.12<br>14.29	19.41
**cttADP-10**	$n_N \rightarrow \sigma^*_{NH}$	C_5	1.13	1.13
**cttADP-11**	$n_O \rightarrow \sigma^*_{NH}$<br>$n'_O \rightarrow \sigma^*_{NH}$	C_7	3.58<br>2.83	6.41
	$n_O \rightarrow \sigma^*_{OH}$<br>$n'_O \rightarrow \sigma^*_{OH}$	C_7	4.70<br>9.39	14.09
**cctADP-12**	No interaction			
**tctADP-13**	$\pi_{CO} \rightarrow \sigma^*_{OH}$<br>$n_O \rightarrow \sigma^*_{OH}$<br>$n'_O \rightarrow \sigma^*_{OH}$	C_8	0.58<br>5.70<br>15.98	22.26
	$n'_O \rightarrow \sigma^*_{NH}$	C_5	1.00	1.00
**tttADP-14**	$\pi_{CO} \rightarrow \sigma^*_{NH}$<br>$n_O \rightarrow \sigma^*_{NH}$<br>$n'_O \rightarrow \sigma^*_{NH}$	C_7	0.72<br>1.17<br>1.74	3.63
**cctADP-15**	$n_O \rightarrow \sigma^*_{NH}$<br>$n'_O \rightarrow \sigma^*_{NH}$	C_{10}	1.05<br>2.41	3.46
**tctADP-16**	$n_O \rightarrow \sigma^*_{NH}$<br>$n'_O \rightarrow \sigma^*_{NH}$	C_{10}	1.08<br>1.39	2.47
**tctADP-17**	$n'_O \rightarrow \sigma^*_{NH}$	C_6	0.68	0.68
**cctADP-18**	$\pi_{CO} \rightarrow \sigma^*_{OH}$<br>$n_O \rightarrow \sigma^*_{OH}$<br>$n'_O \rightarrow \sigma^*_{OH}$	C_7	2.01<br>4.31<br>11.11	17.43

**cctADP-19**	$\pi_{CO} \longrightarrow \sigma^*_{OH}$<br>$n_O \longrightarrow \sigma^*_{OH}$	C_{10}	4.44<br>2.95	7.39
**tctADP-20**	$\pi_{CO} \longrightarrow \sigma^*_{NH}$<br>$n_O \longrightarrow \sigma^*_{NH}$<br>$n'_O \longrightarrow \sigma^*_{NH}$	C_8	0.52<br>0.60<br>1.36	2.48
	$\pi_{CO} \longrightarrow \sigma^*_{OH}$<br>$n_O \longrightarrow \sigma^*_{OH}$<br>$n'_O \longrightarrow \sigma^*_{OH}$	C_{10}	2.59<br>0.64<br>0.50	3.73
	$n_N \longrightarrow \sigma^*_{OH}$	C_5	1.53	1.53
**tttADP-21**	$n_O \longrightarrow \sigma^*_{NH}$<br>$n'_O \longrightarrow \sigma^*_{NH}$	C_8	2.91<br>2.89	5.80
**tctADP-22**	$\pi_{CO} \longrightarrow \sigma^*_{NH}$<br>$n_O \longrightarrow \sigma^*_{NH}$	C_{10}	0.59<br>1.67	2.26
	$\pi_{CO} \longrightarrow \sigma^*_{OH}$<br>$n_O \longrightarrow \sigma^*_{OH}$	C_{10}	6.65<br>4.43	11.08
**cttADP-23**	$\pi_{CO} \longrightarrow \sigma^*_{OH}$<br>$n_O \longrightarrow \sigma^*_{OH}$<br>$n'_O \longrightarrow \sigma^*_{OH}$	C_7	3.74<br>3.90<br>5.00	12.64
	$n_N \longrightarrow \sigma^*_{NH}$	C_5	1.07	1.07
**ttcADP-24**	$\pi_{CO} \longrightarrow \sigma^*_{OH}$<br>$n_O \longrightarrow \sigma^*_{OH}$<br>$n'_O \longrightarrow \sigma^*_{OH}$	C_7	3.80<br>4.73<br>6.76	15.29
**tccADP-25**	$\pi_{CO} \longrightarrow \sigma^*_{OH}$<br>$n_O \longrightarrow \sigma^*_{OH}$<br>$n'_O \longrightarrow \sigma^*_{OH}$	C_7	0.57<br>4.26<br>10.55	15.83
**cttADP-26**	$n_N \longrightarrow \sigma^*_{NH}$	C_5	0.59	0.59
	$n_N \longrightarrow \sigma^*_{OH}$	C_6	4.34	4.34
**cttADP-27**	$n_N \longrightarrow \sigma^*_{NH}$	C_5	0.82	0.82
	$n_N \longrightarrow \sigma^*_{OH}$	C_5	3.00	3.00
**cccADP-28**	$\pi_{CO} \longrightarrow \sigma^*_{OH}$<br>$n_O \longrightarrow \sigma^*_{OH}$<br>$n'_O \longrightarrow \sigma^*_{OH}$	C_{10}	1.75<br>3.28<br>0.64	5.67
**cctADP-29**	$n_N \longrightarrow \sigma^*_{OH}$	C_6	10.90	10.90
**cccADP-30**	$n_O \longrightarrow \sigma^*_{OH}$<br>$n'_O \longrightarrow \sigma^*_{OH}$	C_7	5.23<br>7.29	12.52

Table S18: NBO orbital interactions, E(2) values, corresponding to hydrogen bond for AAP with B3LYP-D_3 method.

	Donor ⟶ Acceptor	Cycle	E(2) (Kcal/mol)	ΣE_{HB} (Kcal/mol)
**tttAAP-1**	$\pi_{CO} \rightarrow \sigma^*_{NH}$<br>$n_O \rightarrow \sigma^*_{NH}$	C_{10}	1.94<br>2.21	4.15
**tttAAP-2**	$\pi_{CO} \rightarrow \sigma^*_{NH}$<br>$n_O \rightarrow \sigma^*_{NH}$<br>$n'_O \rightarrow \sigma^*_{NH}$	C_7	1.24<br>0.63<br>0.68	2.55
**tttAAP-3**	$\pi_{CO} \rightarrow \sigma^*_{NH}$<br>$n_O \rightarrow \sigma^*_{NH}$<br>$n'_O \rightarrow \sigma^*_{NH}$	C_7	0.68<br>1.29<br>1.73	3.70
**tttAAP-4**	$\pi_{CO} \rightarrow \sigma^*_{NH}$<br>$n_O \rightarrow \sigma^*_{NH}$<br>$n'_O \rightarrow \sigma^*_{NH}$	C_7	0.96<br>0.98<br>1.14	3.08
	$n'_O \rightarrow \sigma^*_{NH}$	C_5	0.96	0.96
**tctAAP-5**	$\pi_{CO} \rightarrow \sigma^*_{NH}$<br>$n_O \rightarrow \sigma^*_{NH}$<br>$n'_O \rightarrow \sigma^*_{NH}$	C_{10}	0.68<br>4.07<br>5.37	10.12
**cttAAP-6**	$\pi_{CO} \rightarrow \sigma^*_{NH}$<br>$n_O \rightarrow \sigma^*_{NH}$<br>$n'_O \rightarrow \sigma^*_{NH}$	C_7	0.61<br>1.67<br>1.99	4.27
**cttAAP-7**	$\pi_{CO} \rightarrow \sigma^*_{NH}$<br>$n_O \rightarrow \sigma^*_{NH}$<br>$n'_O \rightarrow \sigma^*_{NH}$	C_7	0.86<br>3.45<br>4.94	9.25
**tctAAP-8**	$n_N \rightarrow \sigma^*_{NH}$	C_5	1.44	1.44
**tttAAP-9**	$n_N \rightarrow \sigma^*_{NH}$	C_5	1.80	1.80
**tctAAP-10**	$\pi_{CO} \rightarrow \sigma^*_{NH}$	C_8	0.88	0.88
**cttAAP-11**	$n_N \rightarrow \sigma^*_{NH}$	C_5	1.15	1.15
**tctAAP-12**	$n'_O \rightarrow \sigma^*_{NH}$	C_5	1.43	1.43
	$n_N \rightarrow \sigma^*_{NH}$	C_5	1.48	1.48
**cctAAP-13**	$n_N \rightarrow \sigma^*_{NH}$	C_5	1.11	1.11
**tttAAP-14**	$n_O \rightarrow \sigma^*_{NH}$<br>$n'_O \rightarrow \sigma^*_{NH}$	C_8	2.77<br>2.79	5.56
**cctAAP-15**	No interaction			
**cttAAP-16**	$n_N \rightarrow \sigma^*_{NH}$	C_5	1.01	1.01
**cctAAP-17**	No interaction			
**ttcAAP-18**	No interaction			
**cctAAP-19**	$n_N \rightarrow \sigma^*_{NH}$	C_7	4.86	4.86
**cccAAP-20**	No interaction			
**tccAAP-21**	No interaction			
**cccAAP-22**	No interaction			

Table S19. Dihedral angle values (in degrees) of all the optimized structures of Ac-Asn-Pro-NHMe at the B3LYP/6-311++G(d,p) level of theory in gas phase.

No.	ΔE (kcal/mol)	ω_0	φ_1	ψ_1	ω_1	φ_2	ψ_2	ω_2	χ_1'	χ_2'	χ_1	χ_2	χ_3	χ_4	Conformer
**tttNP-01**	**0.00**	177.4	-163.2	161.8	-174.1	-84.5	69.3	-178.3	-121.8	59.1	32.3	-37.7	27.8	-7.8	$\varepsilon''\gamma'$(s-)
**tttNP-02**	**1.54**	173.9	-160.8	166.1	-176.0	-94.7	13.9	175.3	67.8	-106.0	35.2	-37.7	25.4	-3.4	$\varepsilon''\delta_R$(g+)
**tttNP-03**	**1.59**	174.7	-74.2	151.2	166.3	-55.9	141.5	-178.1	56.0	109.6	-27.0	37.2	-32.7	16.6	$\beta_P\beta_P$(g+)
**tttNP-04**	**2.29**	176.5	-58.7	119.6	179.8	81.9	5.6	-178.4	47.8	115.9	-35.2	37.1	-24.4	2.1	$\beta_P\delta_L$(g+)
**tttNP-05**	**2.43**	-169.0	-125.4	77.5	-170.1	-84.0	76.9	-176.4	55.9	139.9	-9.7	29.4	-37.7	33.3	?γ'(g+)
**tttNP-06**	**3.19**	178.2	-176.5	161.5	-179.8	-85.7	69.9	-171.9	48.5	148.2	31.8	-37.3	27.8	-8.1	$\beta_S\gamma'$(g+)
**tttNP-07**	**3.29**	174.2	-60.1	121.5	179.5	85.3	3.7	-178.4	-172.6	-135.8	-35.2	36.8	-23.7	1.3	$\beta_P\delta_L$(t)
**tctNP-08**	**3.42**	-179.7	-167.4	154.1	-0.2	-86.1	0.1	-179.6	-126.5	65.1	33.5	-37.4	26.4	-5.3	$\beta_S\delta_R$(s-)
**tctNP-09**	**3.86**	-167.5	-122.9	70.4	3.5	-103.0	144.9	177.1	57.5	133.4	36.4	-32.1	15.2	8.5	$\xi\beta_S$(g+)
**tctNP-10**	**3.88**	-172.5	-124.6	42.0	6.6	-95.9	-8.3	177.8	64.8	148.2	34.1	-37.3	25.8	-18.7	?δ_R(g+)
**tttNP-11**	**4.49**	174.8	-74.4	152.0	166.1	-56.4	138.7	-18.5	56.0	110.0	-26.4	37.2	-33.2	17.5	$\beta_P\beta_P$(g+)
**tccNP-12**	**4.64**	176.4	-168.8	165.3	-6.0	-71.5	166.2	1.4	-120.3	70.8	33.0	-37.0	26.3	-5.5	$\beta_S\beta_P$(s-)
**tttNP-13**	**4.67**	-173.2	-71.1	-31.5	-172.5	63.5	-145.5	177.8	50.3	102.8	-26.0	36.0	-31.8	16.2	$\delta_R\varepsilon$(g+)
**tctNP-14**	**4.71**	-168.4	-126.6	83.7	-2.2	-97.6	139.4	178.2	38.1	53.1	36.0	-30.5	13.2	10.4	$\xi\beta_S$(g+)
**ttcNP-15**	**4.89**	174.1	-74.7	153.3	169.9	-70.2	156.9	-11.3	57.5	107.9	28.7	-35.8	28.9	-11.2	$\beta_P\beta_P$(g+)
**tttNP-16**	**5.60**	-175.7	-68.3	-12.7	171.8	-96.2	9.0	176.2	59.9	128.2	35.7	-33.8	18.9	3.9	$\delta_R\delta_R$(g+)
**ttcNP-17**	**7.53**	-173.1	-71.0	-31.5	-172.7	64.4	-146.3	16.8	49.8	102.7	-26.2	36.0	-31.6	15.8	$\delta_R\varepsilon$(g+)
**tttNP-18**	**7.72**	-170.7	-58.9	-31.9	177.5	-71.6	-12.4	178.8	-154.4	-19.2	-22.9	36.6	-36.0	22.7	$\alpha_R\delta_R$(t)

Table S20. The relative energies (DE, in kcal/mol) and dihedral angle values (in degrees) of all the optimized structures of Ac-Asn-Pro-NHMe at the B3LYP-D3/6-311++G(d,p) level of theory in gas phase.

No.	ΔE	ω_0	φ_1	ψ_1	ω_1	φ_2	ψ_2	ω_2	χ_1'	χ_2'	χ_1	χ_2	χ_3	χ_4	Conformer
**tttNP-01**	**0.00**	178.4	-164.6	162.3	-175.7	-84.8	69.2	-177.0	-122.6	58.0	33.7	-38.5	27.8	-6.8	$\beta_S\gamma'$(s-)
**tttNP-02**	**0.58**	-179.6	173.2	169.1	178.4	-92.1	17.4	174.9	54.5	-100.1	34.9	-39.3	28.3	-6.4	$\varepsilon\delta_R$(g+)
**tttNP-03**	**1.00**	171.9	-68.7	150.5	164.5	-53.5	134.6	-175.1	56.2	109.2	-27.5	37.9	-33.2	16.7	$\beta_P\beta_P$(g+)
**tttNP-04**	**0.90**	175.8	-57.84	117.5	-179.6	78.1	9.5	-178.8	46.3	108.0	-35.6	38.3	-25.8	3.3	$\beta\delta_L$(g+)
**tttNP-05**	**3.03**	-169.3	-127.4	72.4	-168.5	-85.4	76.2	-174.9	57.4	137.3	-8.7	29.5	-38.9	35.2	$\xi\gamma'$(g+)
**tttNP-06**	**1.90**	-178.9	177.5	162.9	175.5	-85.8	68.2	-170.2	-48.4	-99.7	33.4	-38.4	28.0	-7.2	$\varepsilon\gamma'$(g-)
**tttNP-07**	**1.58**	172.6	-59.9	123.3	-180.0	82.7	5.4	-178.6	177.5	-99.8	-35.8	37.9	-25.0	2.3	$\beta_P\delta_L$(t)
**tctNP-08**	**2.60**	-179.1	-167.4	154.5	-2.4	-79.8	-2.1	-179.0	-128.6	64.0	33.1	-38.6	28.6	-7.9	$\beta_S\delta_R$(s-)
**tctNP-09**	**1.27**	-163.7	-125.6	56.4	4.8	-95.7	155.0	177.7	54.4	106.6	36.5	-36.6	22.4	1.0	$\xi\varepsilon''$(g+)
**tctNP-10**	**0.45**	-176.5	-113.9	25.3	9.0	-88.8	-3.9	174.5	69.8	159.2	34.3	-39.2	28.7	7.3	$\delta_R\delta_R$(g+)
**tttNP-11**	**3.29**	171.8	-68.5	152.0	164.5	-54.5	131.8	-20.5	56.4	109.4	-26.9	37.4	-33.8	17.7	$\beta_P\beta_P$(g+)
**tccNP-12**	**2.69**	176.2	-166.1	166.6	-6.6	-67.2	165.9	1.7	-121.3	63.6	32.8	-38.3	28.6	-8.1	$\varepsilon''\beta_P$(s-)
**tttNP-13**	**2.00**	-177.2	-60.9	-33.5	-172.6	62.2	-142.3	176.7	50.9	100.1	-25.9	37.1	-33.8	18.3	$\alpha_R\varepsilon$(g+)
**tctNP-14**	**1.27**	-163.7	-125.6	56.4	4.8	-95.7	154.9	177.7	54.4	106.6	36.5	-36.6	22.4	1.0	?ε''(g+)
**ttcNP-15**	**3.72**	170.9	-68.8	154.3	167.9	-63.2	136.2	-19.5	58.3	107.6	26.8	-36.3	31.3	-15.2	$\beta_P\beta_P$(g+)
**tttNP-16**	**3.33**	-178.1	-63.5	-17.6	173.7	-95.7	9.2	176.6	63.4	110.1	37.3	-34.1	17.9	6.1	$\delta_R\delta_R$ (g+)
**ttcNP-17**	**4.23**	-177.4	-60.5	-34.0	-172.2	62.2	-137.5	21.1	50.5	100.9	-25.2	36.4	18.7	4.1	$\alpha_R\varepsilon$(g+)
**tttNP-18**	**6.46**	-174.2	-55.9	-33.5	177.8	-68.4	-15.0	178.6	-157.8	-15.3	-24.0	37.6	-36.3	22.3	$\alpha_R\delta_R$(t)
**ttcNP-19**	**4.13**	-165.8	-127.2	52.9	2.0	-90.0	166.1	1.3	53.6	99.6	36.6	-37.4	23.5	-0.1	?β_P(g+)

Table S21. The relative energies (ΔE, in kcal/mol) and dihedral angle values (in degrees) of all the optimized structures of Ac-Asp-Pro-NHMe (**8**, **DP**) at the B3LYP/6-311++G(d,p) level of theory in gas phase.

No.	ΔE	ω_0	φ_1	ψ_1	ω_1	φ_2	ψ_2	ω_2	χ_1'	χ_2'	χ_1	χ_2	χ_3	χ_4	Conformer
**tctDP-01**	**0.00**	-177.1	172.9	-168.2	0.5	70.0	-144.9	-177.4	116.5	-64.7	-30.4	37.1	-29.0	10.2	εε(s+)
**tttDP-02**	**0.92**	171.6	132.1	-67.8	-173.5	-84.3	71.5	-177.3	-58.8	-152.6	31.7	-37.2	27.7	-8.0	?γ'(g-)
**tttDP-03**	**0.92**	-171.6	-132.1	67.9	173.6	84.3	-71.5	177.3	58.8	152.6	-31.7	37.3	-27.7	8.0	ξγ(g+)
**tttDP-04**	**1.51**	-179.8	-141.1	113.6	-175.5	-87.6	-0.7	177.1	-176.5	166.7	34.6	-37.5	25.5	-3.6	$\beta_S\delta_R$(t)
**tttDP-05**	**1.63**	-172.0	-121.8	85.2	-171.8	-83.0	77.2	-176.4	56.0	142.8	-10.9	30.0	-37.4	32.2	ξγ'(g+)
**tttDP-06**	**2.13**	177.5	103.1	-104.2	-176.2	-82.5	72.3	-178.6	-40.2	-46.9	32.7	-38.7	29.0	-8.7	?γ'(g-)
**tttDP-07**	**2.38**	-176.5	170.7	-172.9	-8.0	-102.5	136.5	178.1	113.0	-62.4	36.1	-27.2	7.7	16.2	$\varepsilon\beta_S$(s+)
**tctDP-08**	**2.38**	-176.6	89.9	-119.4	173.2	85.4	-69.2	177.5	-65.6	60.5	-32.4	37.2	-26.9	6.8	?γ(g-)
**tttDP-09**	**2.51**	179.0	55.7	-124.5	-177.2	-82.8	-1.0	177.3	54.8	120.8	35.5	-37.3	24.3	-1.8	$\varepsilon\delta_R$(g+)
**tttDP-10**	**2.70**	-178.2	57.2	-120.0	-178.1	-83.0	-4.0	178.4	-42.8	-44.7	35.5	-37.4	24.4	-1.8	$\varepsilon\delta_R$(g-)
**tttDP-11**	**2.95**	179.6	-100.9	107.8	175.0	81.9	-70.6	178.3	64.4	-54.5	-32.2	38.7	-29.4	9.5	$\beta_S\gamma$(g+)
**tttDP-12**	**3.39**	174.9	-58.1	129.5	179.4	84.3	-0.4	-177.2	-60.7	92.3	-35.4	37.0	-23.9	1.5	$\varepsilon''\delta_L$(g-)
**tttDP-13**	**3.58**	-169.5	-43.0	-54.9	-173.6	-91.1	12.2	175.0	61.6	179.8	34.1	-37.4	26.0	-4.5	$\alpha_R\delta_R$(g+)
**tttDP-14**	**7.84**	-173.8	-98.4	-25.9	-165.0	70.7	-102.6	174.6	67.3	115.1	21.6	-35.6	35.6	-23.1	δ_R?(g+)

Table S22. The relative energies (ΔE, in kcal/mol) and dihedral angle values (in degrees) of all the optimized structures of Ac-Asp-Pro-NHMe (**8**, **DP**) at the B3LYP-D3/6-311++G(d,p) level of theory in gas phase.

No.	ΔE	ω_0	φ_1	ψ_1	ω_1	φ_2	ψ_2	ω_2	χ_1'	χ_2'	χ_1	χ_2	χ_3	χ_4	Conformer
**tctDP-01**	**0.00**	-176.8	170.5	-168.5	2.0	63.9	-147.3	178.8	117.9	-60.6	-30.1	38.5	-31.5	13.1	$\varepsilon\varepsilon$(s+)
**tttDP-02**	**5.40**	179.7	70.2	30.4	166.8	-76.6	97.8	-171.7	-69.4	-107.0	29.4	-36.2	28.7	-10.6	$\delta_L\gamma'$(g-)
**tttDP-03**	**5.52**	178.4	-69.4	-30.4	-166.8	76.6	-97.8	171.7	69.4	107.1	-29.3	36.3	-28.8	10.8	δ_R?(g+)
**tttDP-04**	**3.07**	171.8	-98.9	111.4	-175.8	-86.0	-2.2	177.2	-176.6	172.0	35.9	-37.9	24.8	-2.0	$\beta_S\delta_R$(t)
**tttDP-05**	**14.43**	-179.1	-120.5	28.7	-175.8	32.8	49.4	-172.4	65.4	170.9	-37.4	17.3	8.7	-32.7	δ_R?(g+)
**tttDP-06**	**4.20**	169.7	127.1	-81.9	-179.1	-82.1	73.7	-177.1	-40.1	-58.3	33.6	-39.2	28.9	-8.0	?γ'(g-)
**tttDP-07**	**3.07**	-176.8	170.9	-174.7	-10.8	-97.5	141.5	179.1	114.2	-62.0	37.1	-30.0	10.7	13.7	$\varepsilon\beta_S$(s+)
**tctDP-08**	**4.14**	-170.7	74.1	-133.4	-178.5	85.6	-68.3	172.5	-68.9	67.8	-32.7	37.7	-27.6	7.2	$\varepsilon\gamma$(g-)
**tttDP-09**	**3.83**	-178.9	57.9	-122.3	-178.9	-78.8	-6.3	178.1	55.5	111.8	36.2	-38.2	25.1	-2.1	$\varepsilon\delta_R$(g+)
**tttDP-10**	**3.20**	-175.0	59.5	-119.3	179.6	-79.9	-7.9	178.9	-41.7	-40.5	35.9	-38.4	25.5	-2.7	$\varepsilon\delta_R$(g-)
**tttDP-11**	**5.15**	176.5	-94.9	107.9	176.8	82.0	-70.8	176.8	64.9	-55.7	-33.4	39.5	-29.6	8.9	$\beta_S\gamma$(g+)
**tttDP-12**	**4.58**	174.0	-59.3	125.1	-179.6	80.0	5.7	-178.2	-59.6	90.4	-36.2	38.0	-25.0	1.7	$\beta_P\delta_L$(g-)
**tttDP-13**	**4.39**	-170.4	-44.8	-52.5	-174.5	-90.9	11.7	175.3	61.1	-179.0	35.5	-38.0	25.5	-3.0	?δ_R(g+)
**tttDP-14**	**6.59**	-178.6	-85.0	-29.1	-162.2	70.8	-98.4	172.5	69.9	114.9	20.7	-35.5	36.3	-24.3	δ_R?(g+)

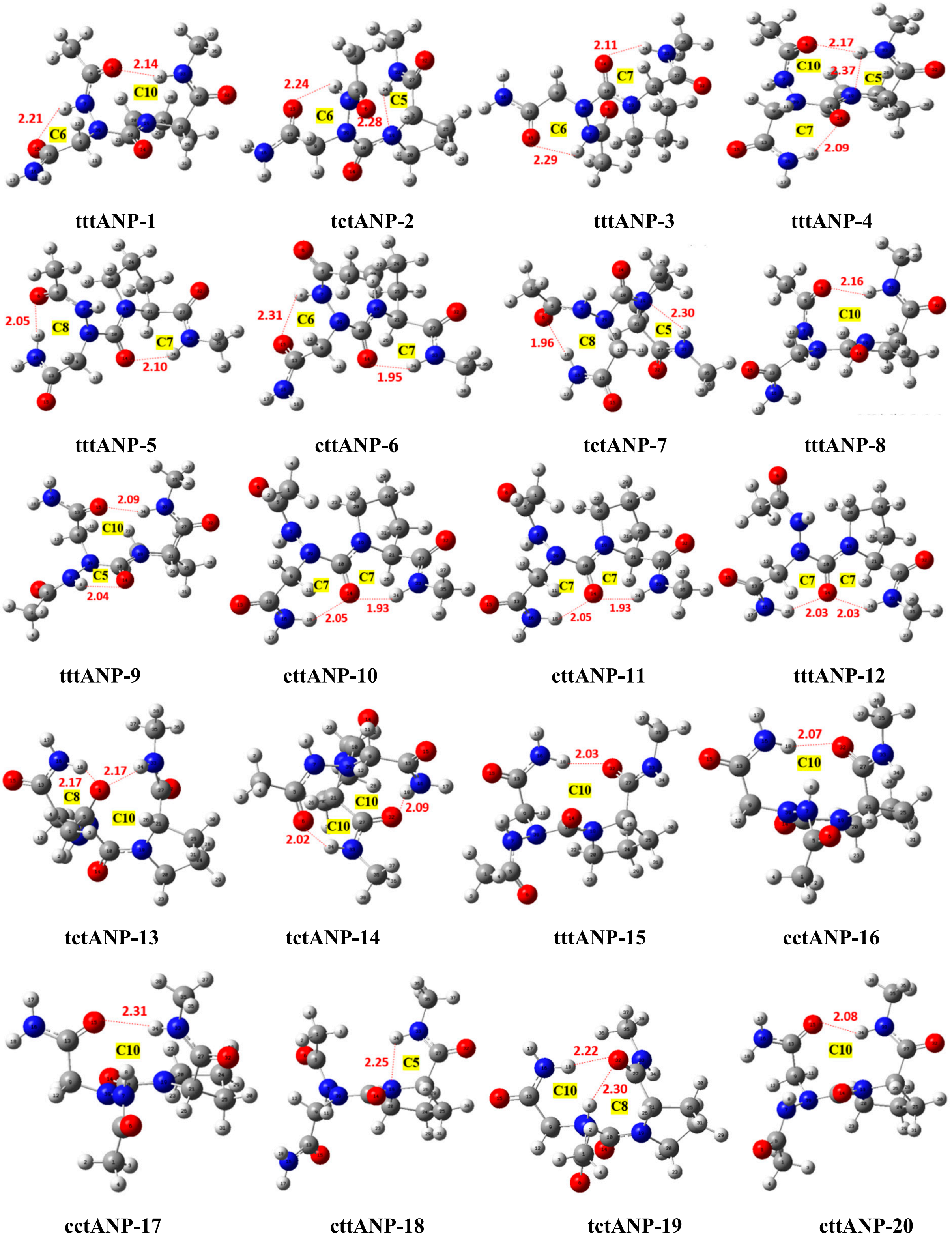
tttANP-1
tctANP-2
tttANP-3
tttANP-4
tttANP-5
cttANP-6
tctANP-7
tttANP-8
tttANP-9
cttANP-10
cttANP-11
tttANP-12
tctANP-13
tctANP-14
tttANP-15
cctANP-16
cctANP-17
cttANP-18
tctANP-19
cttANP-20

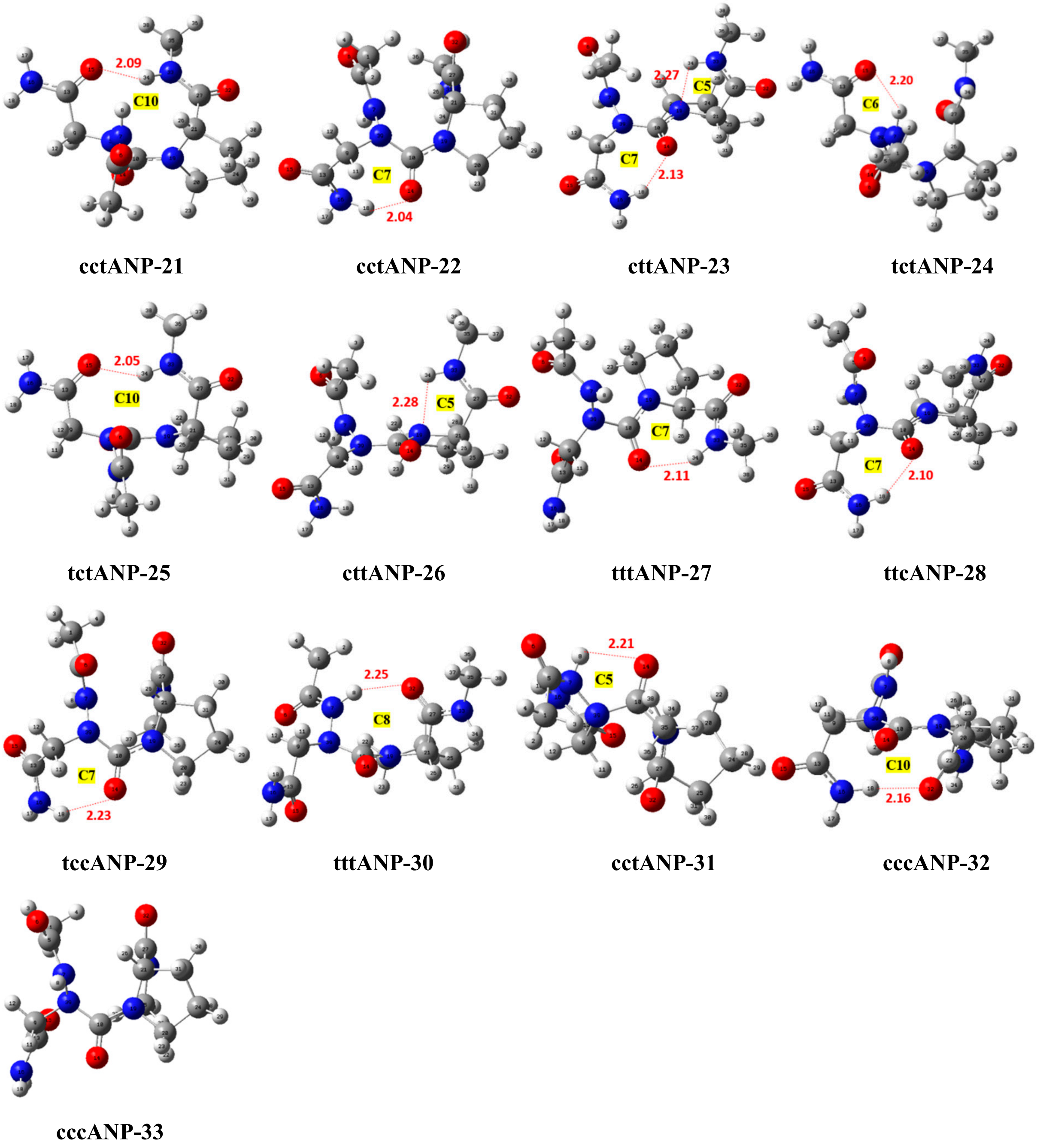

Figure S1. Hydrogen bonds (red dashed line) stabilizing the 33 optimized structures of Ac-azaAsn-Pro-NHMe calculated in the gas phase at the B3LYP/6-311++G (d,p) level of theory.

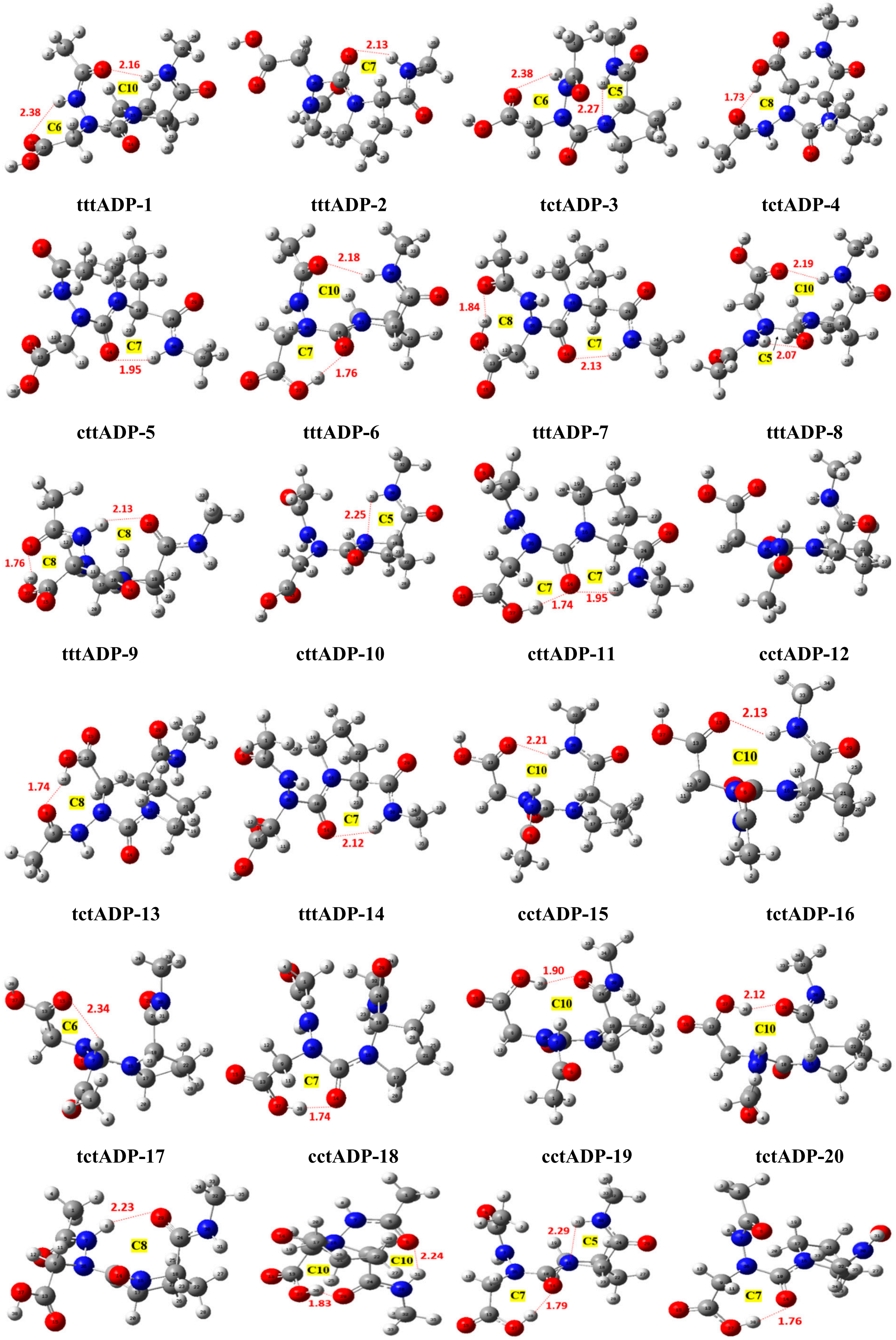
tttADP-1
tttADP-2
tctADP-3
tctADP-4
cttADP-5
tttADP-6
tttADP-7
tttADP-8
tttADP-9
cttADP-10
cttADP-11
cctADP-12
tctADP-13
tttADP-14
cctADP-15
tctADP-16
tctADP-17
cctADP-18
cctADP-19
tctADP-20

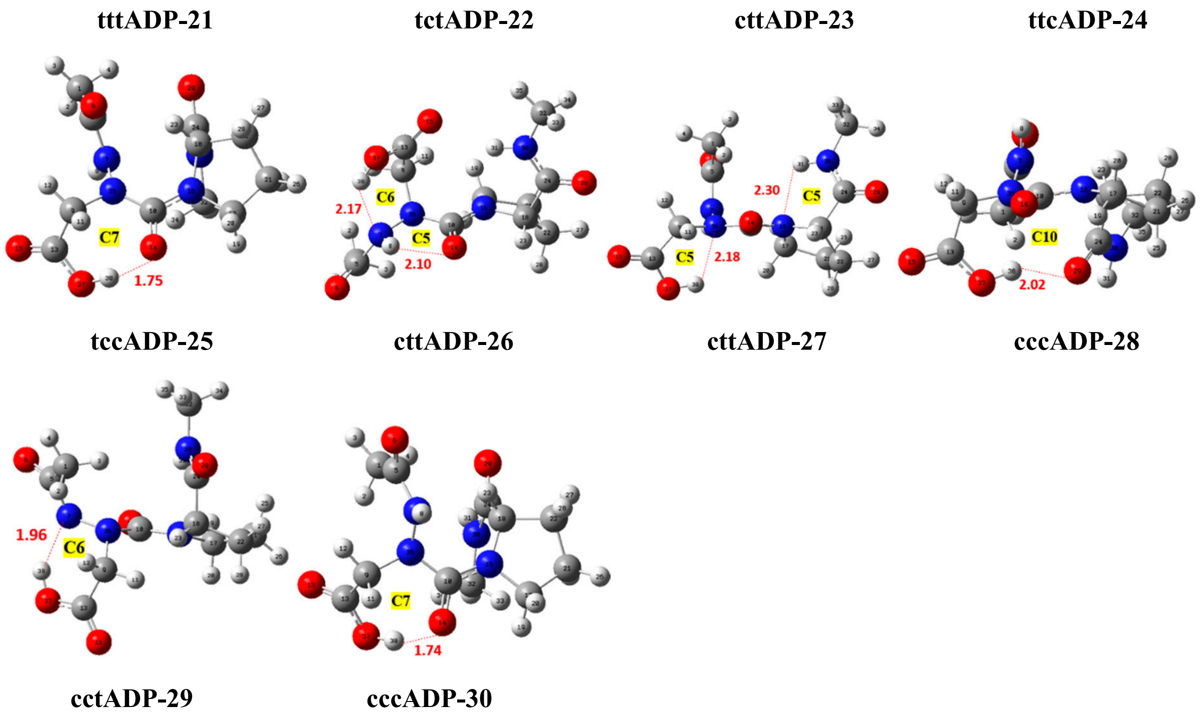

Figure S2. B3LYP/6-311++G(d,p)-optimized structures of Ac-azaAsp-Pro-NHMe. Hydrogen bonds are shown in red dashed line.

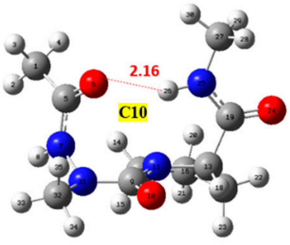

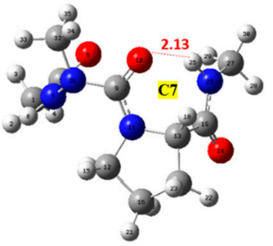

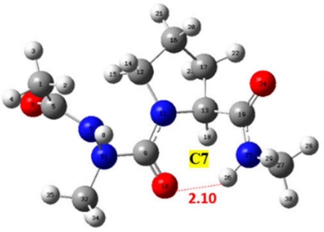

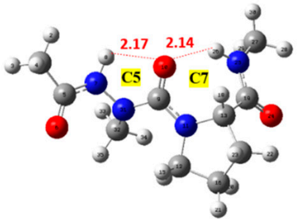

tttAAP-1 **tttAAP-2** **tttAAP-3** **tttAAP-4**

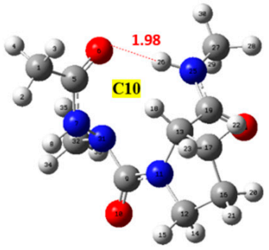

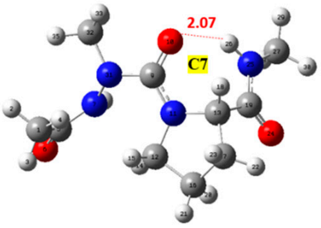

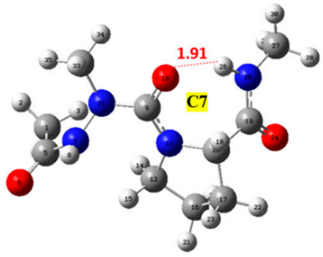

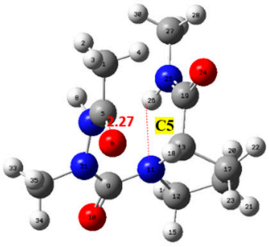

tctAAP-5 **cttAAP-6** **cttAAP-7** **tctAAP-8**

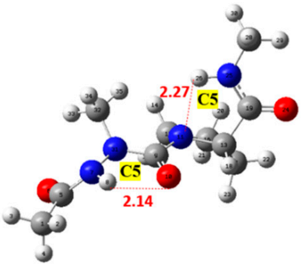

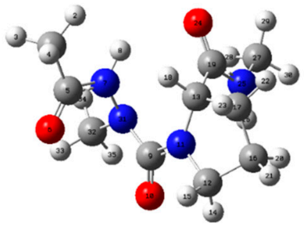

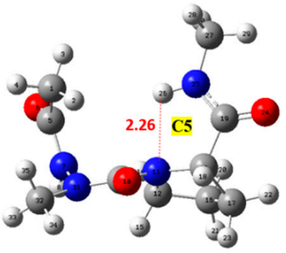

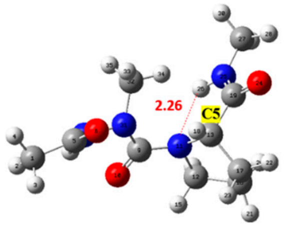

tttAAP-9 **tctAAP-10** **cttAAP-11** **tctAAP-12**

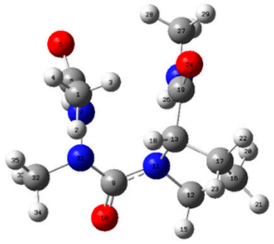

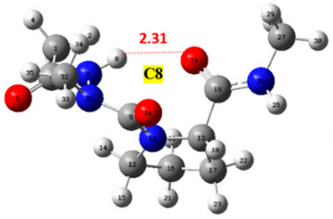

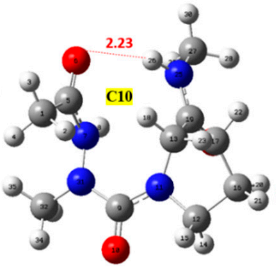

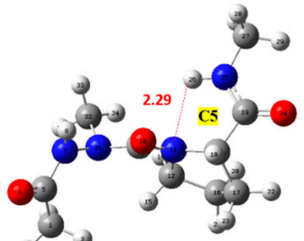

cctAAP-13 **tttAAP-14** **cctAAP-15** **cttAAP-16**

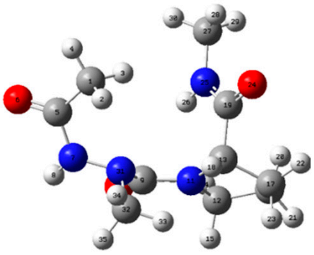

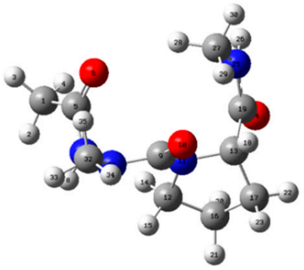

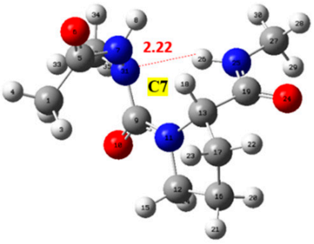

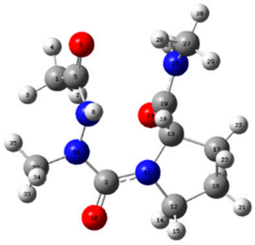

cctAAP-17 **ttcAAP-18** **cctAAP-19** **cccAAP-20**

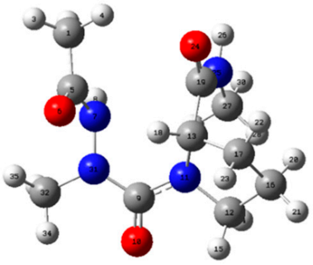

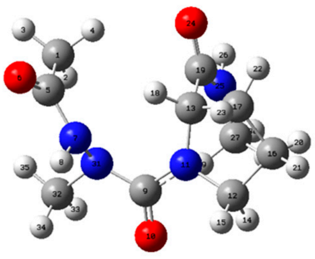

tccAAP-21 **cccAAP-22**

Figure S3. Hydrogen bonds (red dashed line) stabilizing the 22 optimized structures of Ac-azaAla-Pro-NHMe calculated in the gas phase at B3LYP/6-311++G (d,p) level of theory.

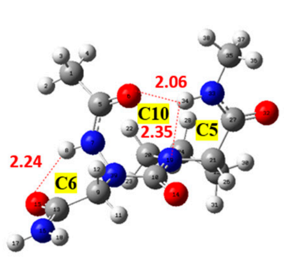

tttANP-1

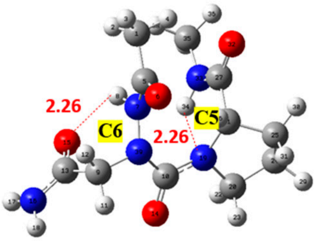

tctANP-2

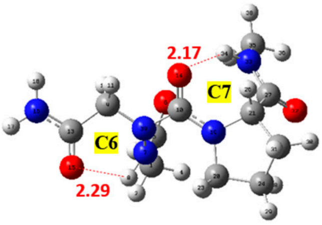

tttANP-3

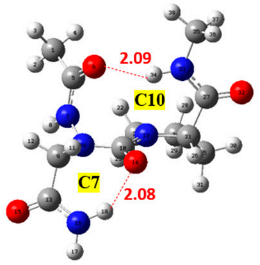

tttANP-4

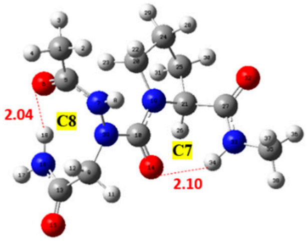

tttANP-5

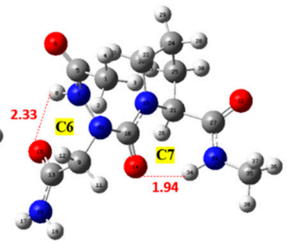

cttANP-6

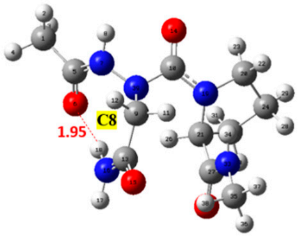

tctANP-7

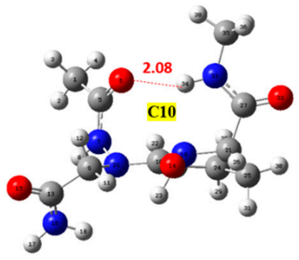

tttANP-8

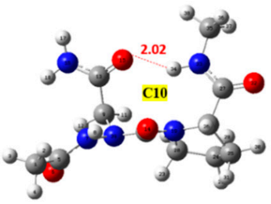

tttANP-9

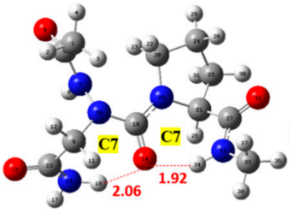

cttANP-10

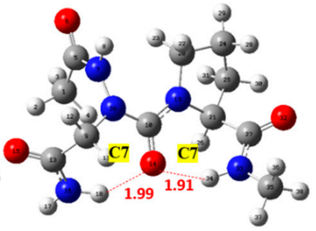

cttANP-11

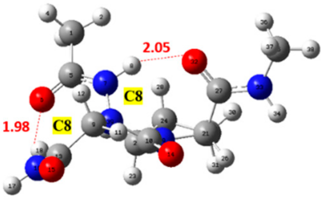

tttANP-12

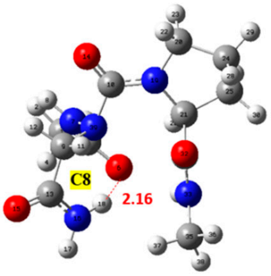

tctANP-13

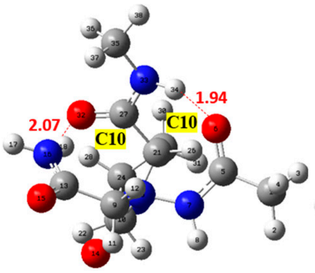

tctANP-14

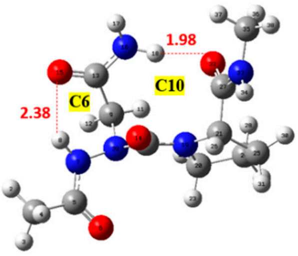

tttANP-15

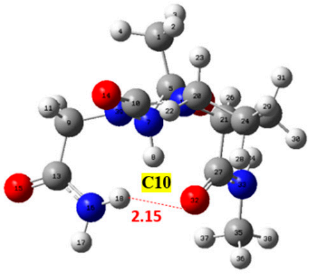

cctANP-16

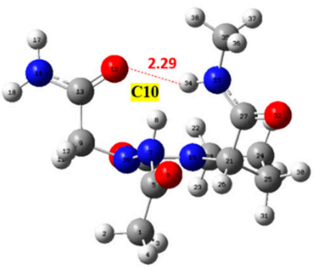

cctANP-17

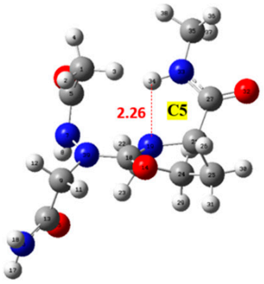

cttANP-18

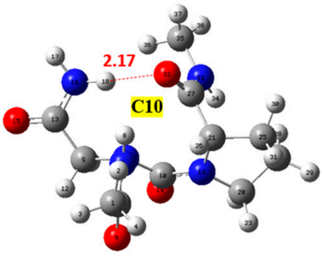

tctANP-19

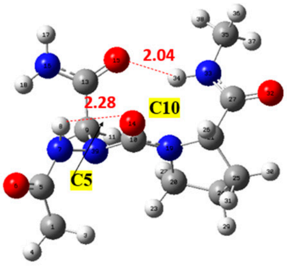

cttANP-20

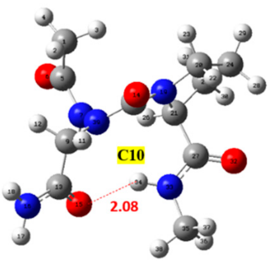

cctANP-21

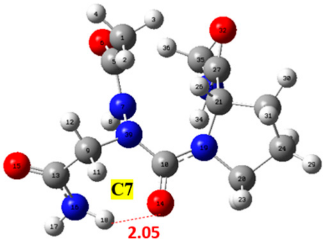

cctANP-22

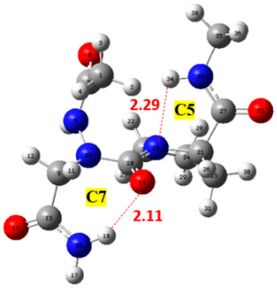

cttANP-23

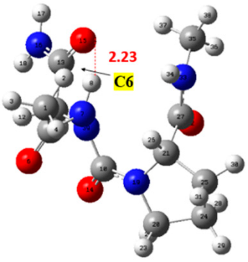

tctANP-24

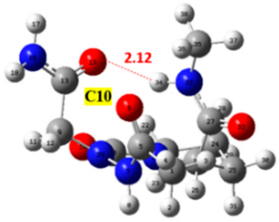

tctANP-25

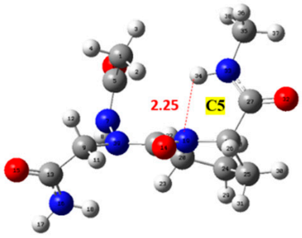

cttANP-26

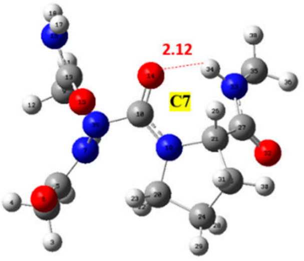

tttANP-27

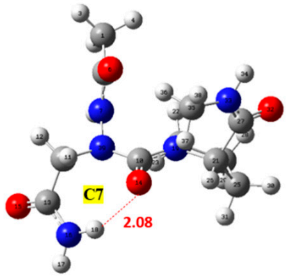

ttcANP-28

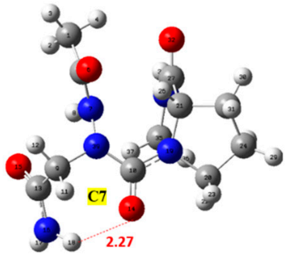

tccANP-29

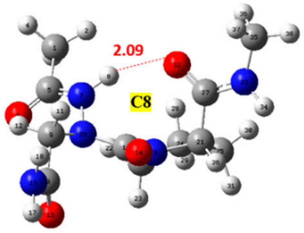

tttANP-30

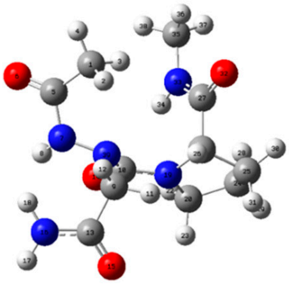

cctANP-31

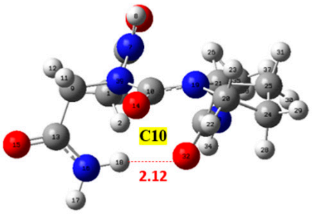

cccANP-32

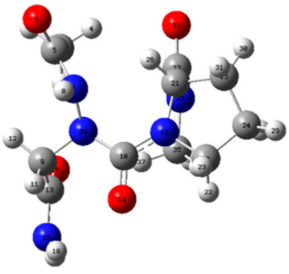

cccANP-33

Figure S4. Hydrogen bonds (red dashed line) stabilizing the 33 optimized structures of Ac-azaAsn-Pro-NHMe calculated in the gas phase at the B3LYP-D_3/6-311++G (d,p) level of theory.

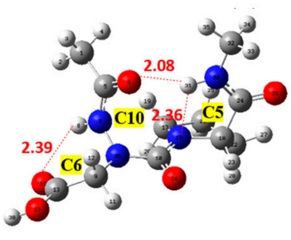

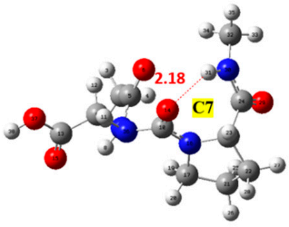

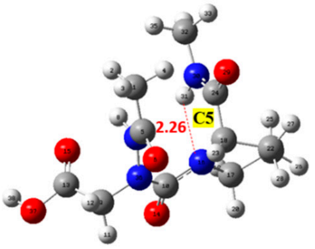

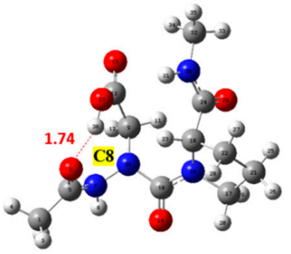

tttADP-1 **tttADP-2** **tctADP-3** **tctADP-4**

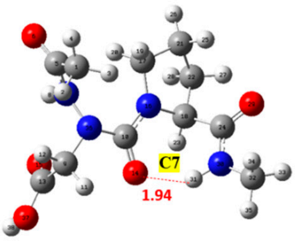

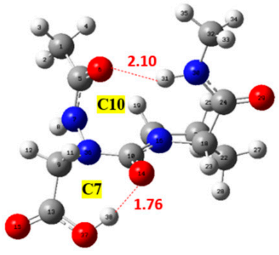

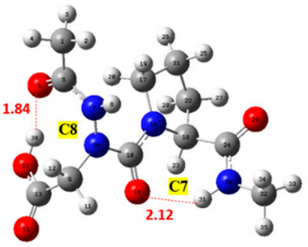

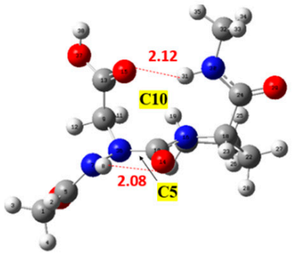

cttADP-5 **tttADP-6** **tttADP-7** **tttADP-8**

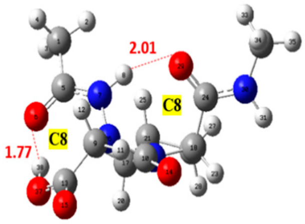

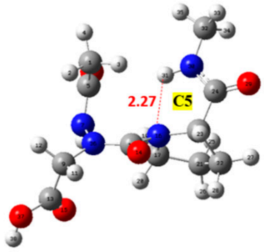

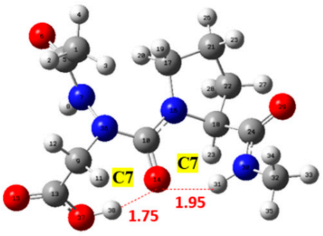

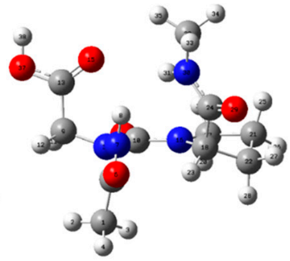

tttADP-9 **cttADP-10** **cttADP-11** **cctADP-12**

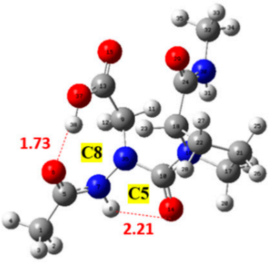

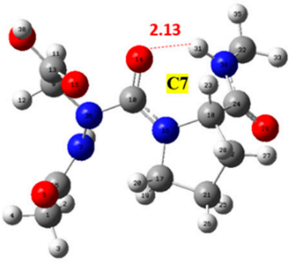

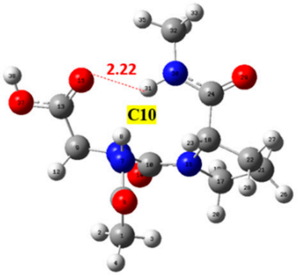

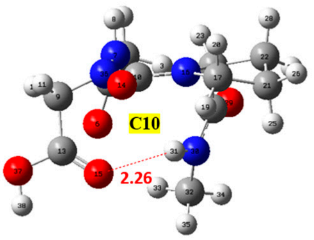

tctADP-13 **tttADP-14** **cctADP-15** **tctADP-16**

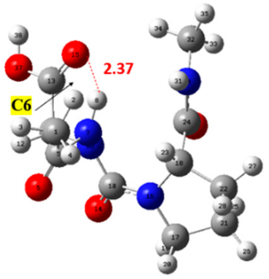

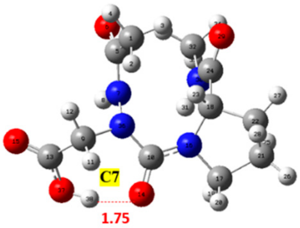

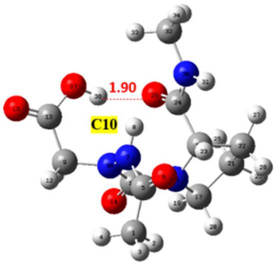

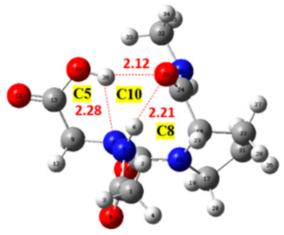

tctADP-17 **cctADP-18** **cctADP-19** **tctADP-20**

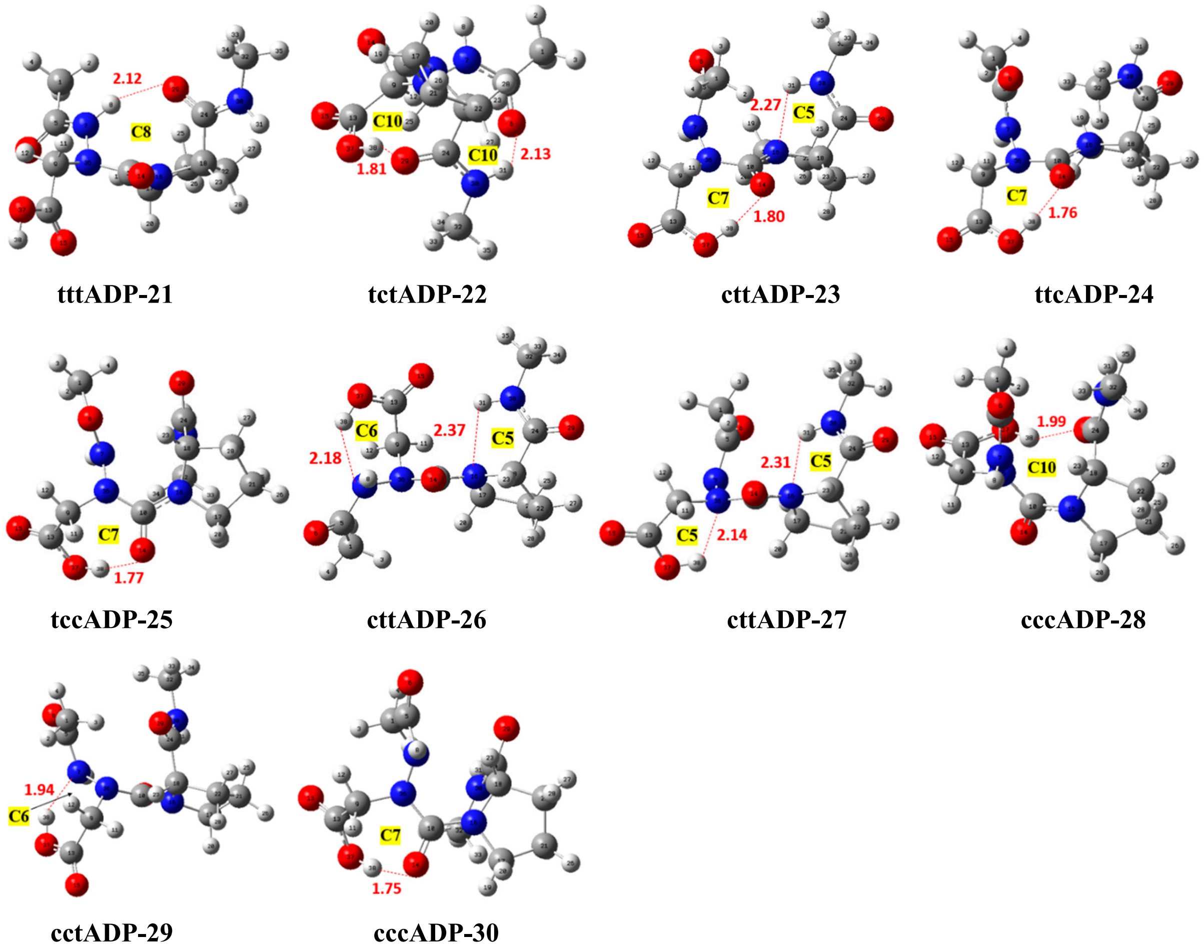

Figure S5. Hydrogen bonds (red dashed line) stabilizing the 30 optimized structures of Ac-azaAsp-Pro-NHMe calculated in the gas phase at B3LYP-D_3/6-311++G (d,p) level of theory.

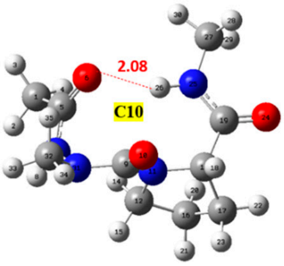

tttAAP-1

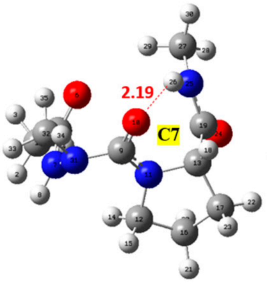

tttAAP-2

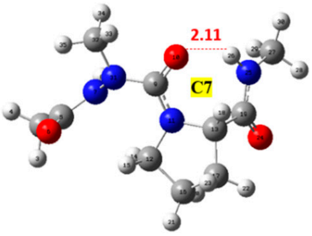

tttAAP-3

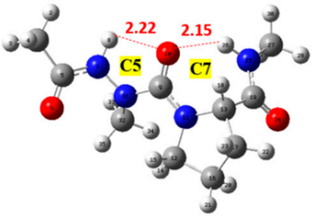

tttAAP-4

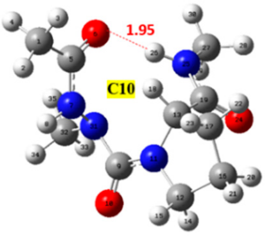

tctAAP-5

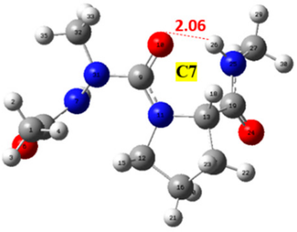

cttAAP-6

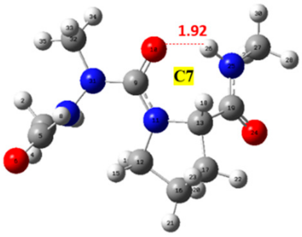

cttAAP-7

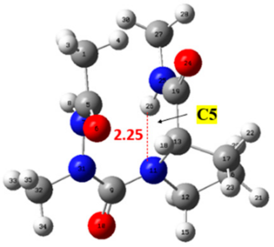

tctAAP-8

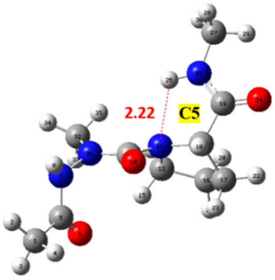

tttAAP-9

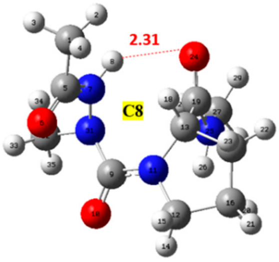

tctAAP-10

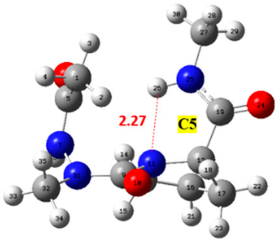

cttAAP-11

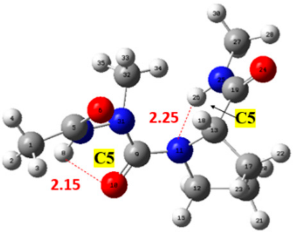

tctAAP-12

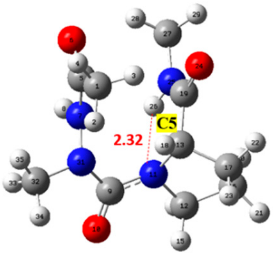

cctAAP-13

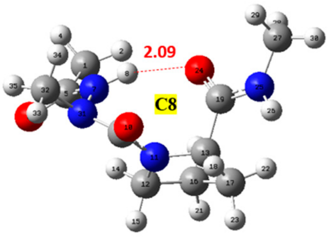

tttAAP-14

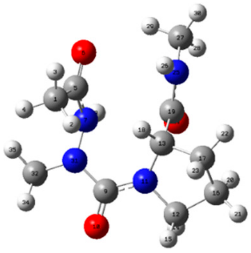

cctAAP-15

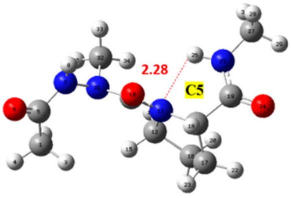

cttAAP-16

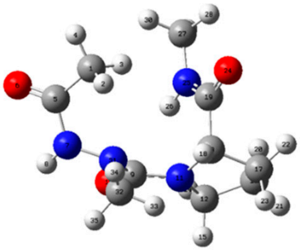

cctAAP-17

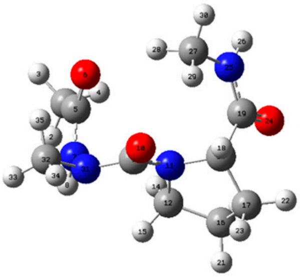

ttcAAP-18

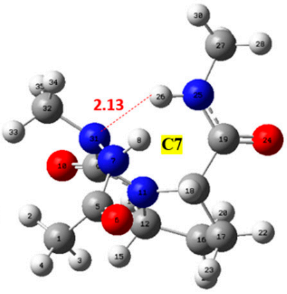

cctAAP-19

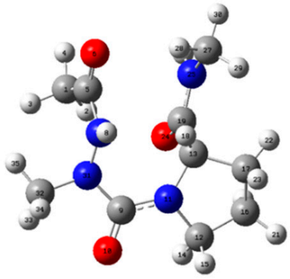

cccAAP-20

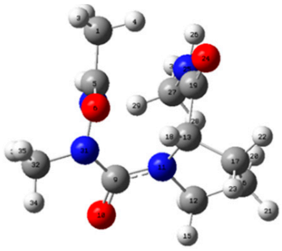

tccAAP-21

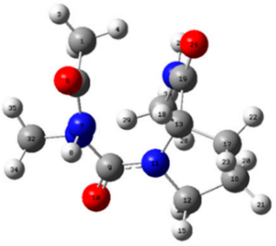

cccAAP-22

Figure S6. Hydrogen bonds (red dashed line) stabilizing the 22 optimized structures of Ac-azaAla-Pro-NHMe calculated in the gas phase at B3LYP-D_3/6-311++G (d,p) level of theory.

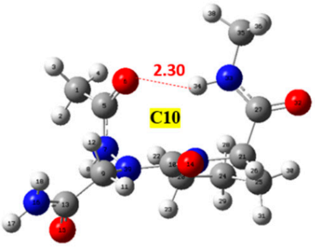

tttANP-1

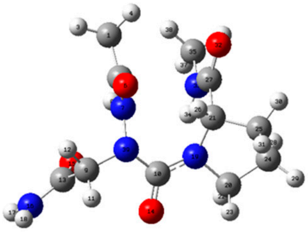

tctANP-2

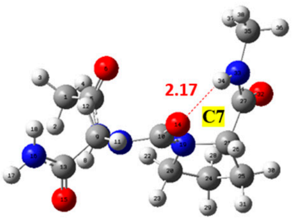

tttANP-3

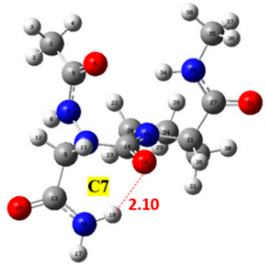

tttANP-4

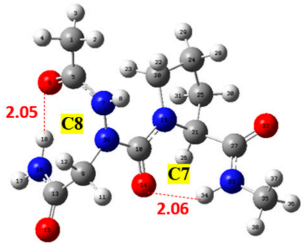

tttANP-5

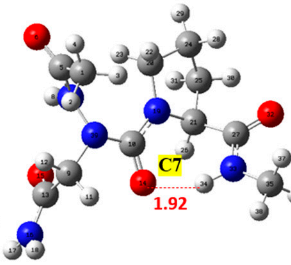

cttANP-6

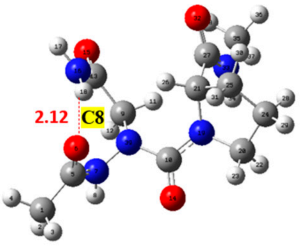

tctANP-7

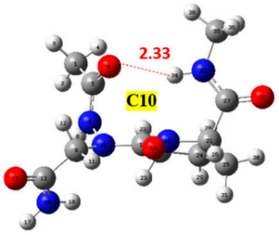

tttANP-8

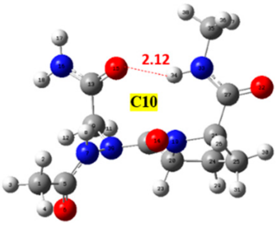

tttANP-9

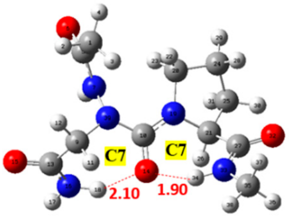

cttANP-10

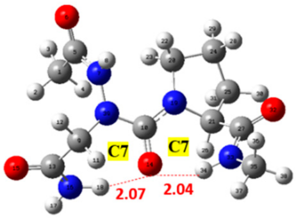

cttANP-11

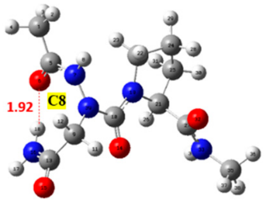

tttANP-12

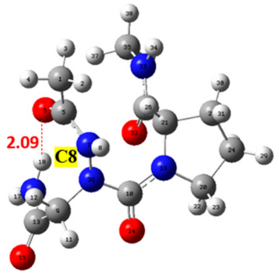

tctANP-13

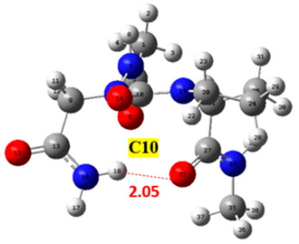

tctANP-14

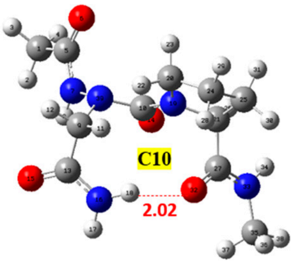

tttANP-15

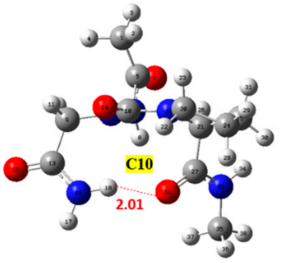

cctANP-16

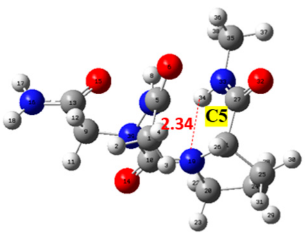

cctANP-17

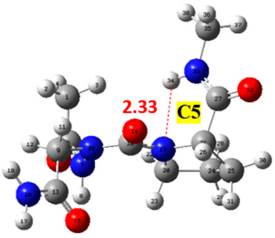

cttANP-18

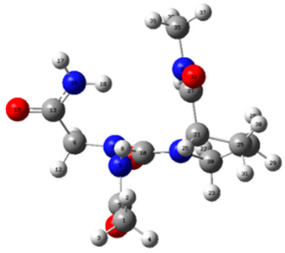

tctANP-19

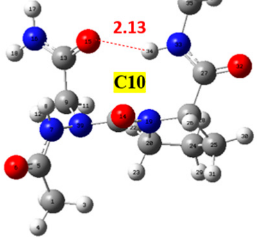

cttANP-20

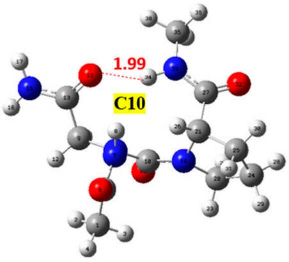

cctANP-21

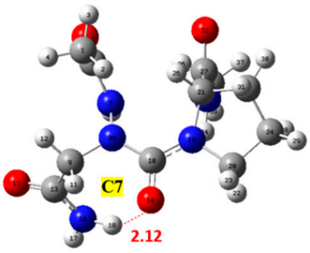

cctANP-22

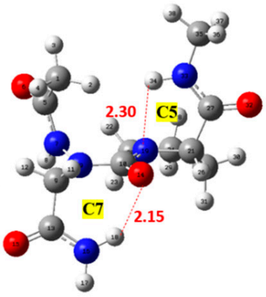

cttANP-23

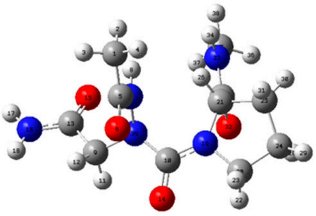

tctANP-24

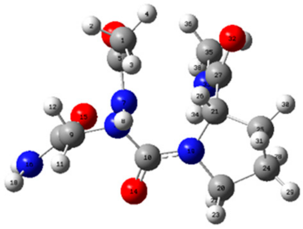

tctANP-25

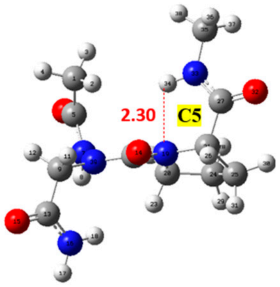

cttANP-26

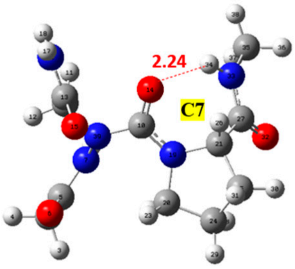

tttANP-27

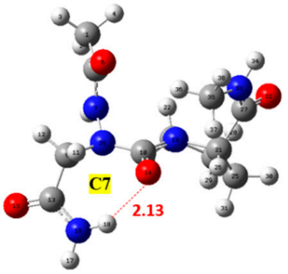

ttcANP-28

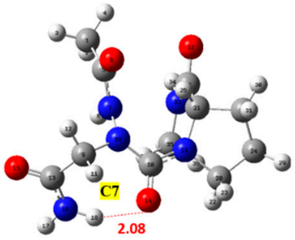

tccANP-29

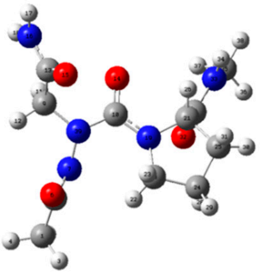

tttANP-30

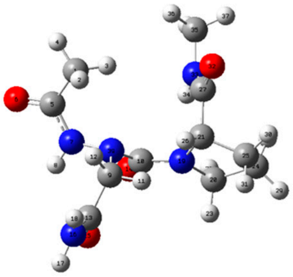

cctANP-31

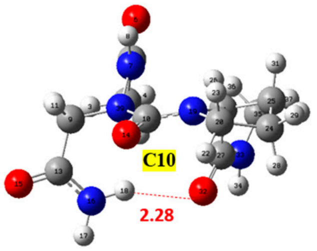

cccANP-32

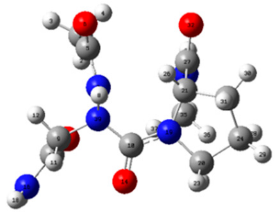

cccANP-33

Figure S7. Hydrogen bonds (red dashed line) stabilizing the 33 optimized structures of Ac-azaAsn-Pro-NHMe calculated in water at the SMD/B3LYP/6-311++G (d,p) level of theory.

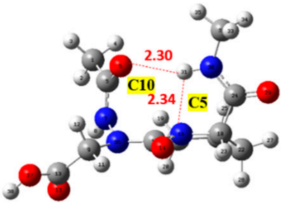

tttADP-1

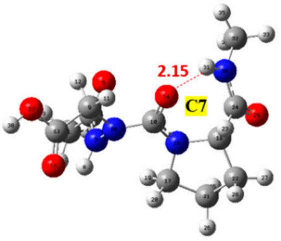

tttADP-2

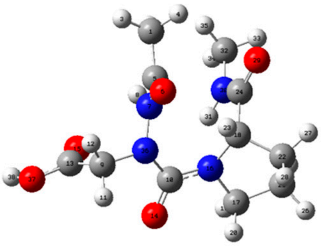

tctADP-3

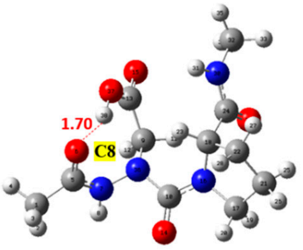

tctADP-4

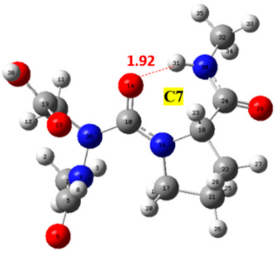

cttADP-5

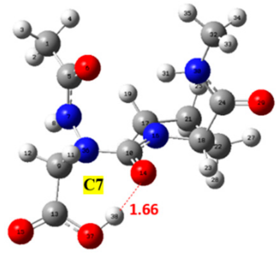

tttADP-6

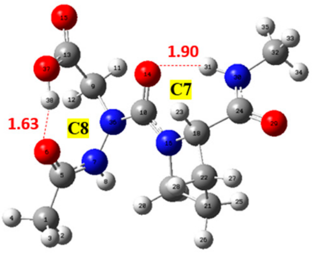

tttADP-7

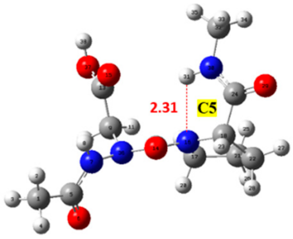

tttADP-8

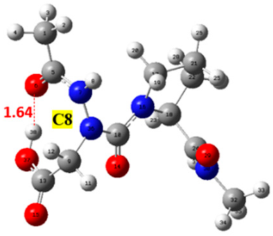

tttADP-9

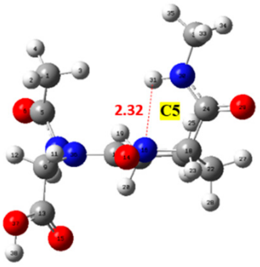

cttADP-10

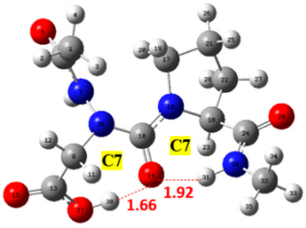

cttADP-11

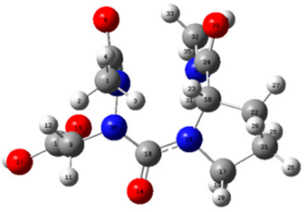

ccADP-12

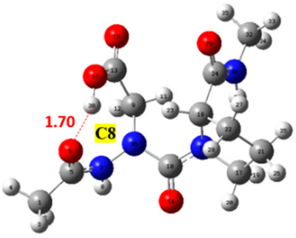

tctADP-13

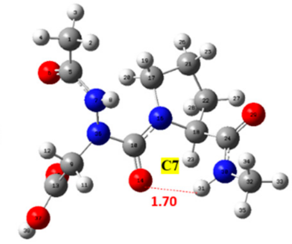

tttADP-14

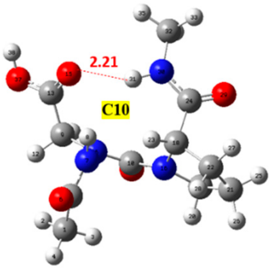

cctADP-15

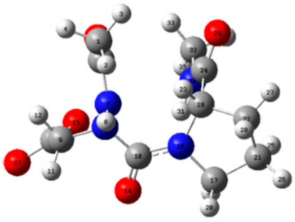

tctADP-16

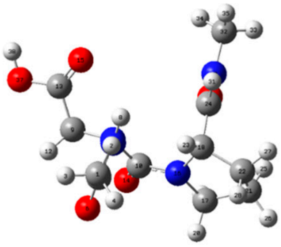

tctADP-17

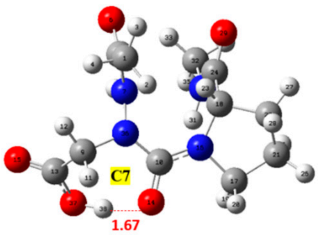

cctADP-18

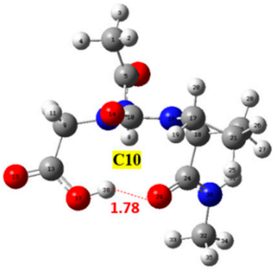

cctADP-19

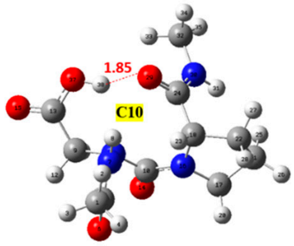

tctADP-20

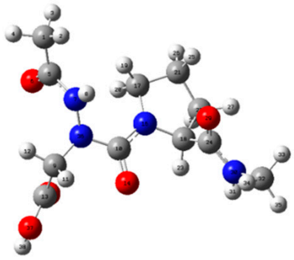

tttADP-21

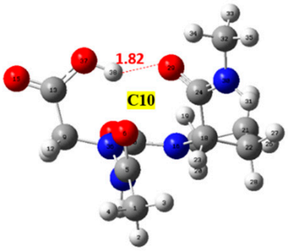

tctADP-22

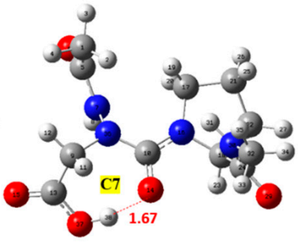

cttADP-23

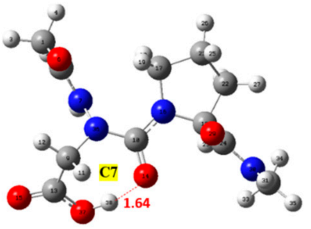

ttcADP-24

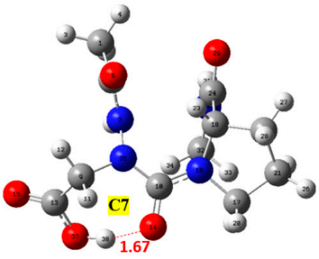

tccADP-25

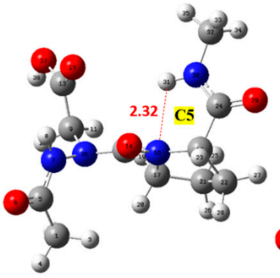

cttADP-26

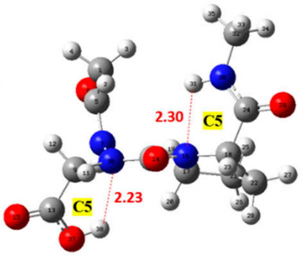

cttADP-27

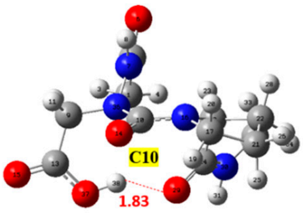

cccADP-28

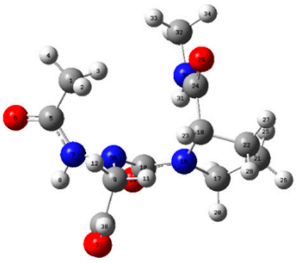

cctADP-29

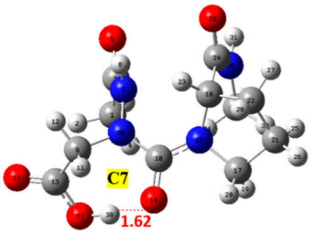

cccADP-30

Figure S8. Hydrogen bonds (red dashed line) stabilizing the 30 optimized structures of Ac-azaAsp-Pro-NHMe calculated in water at the SMD/B3LYP/6-311++G (d,p) level of theory.

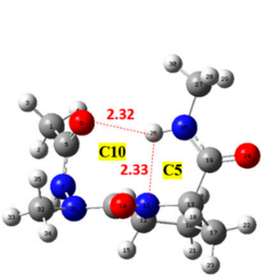

tttAAP-1

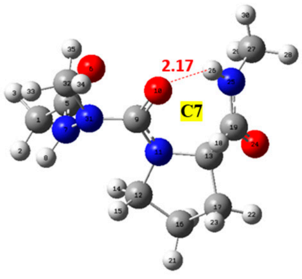

tttAAP-2

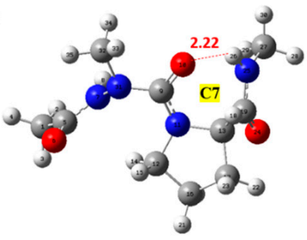

tttAAP-3

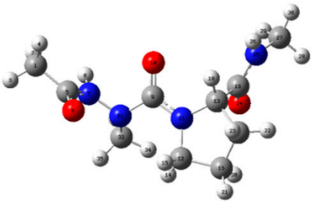

tttAAP-4

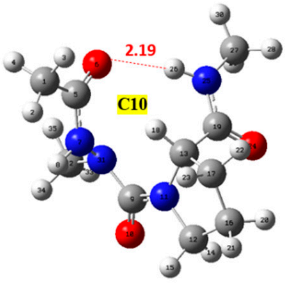

tctAAP-5

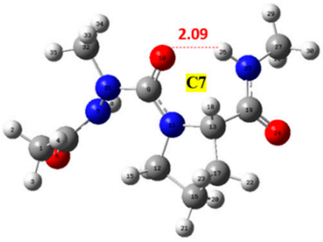

cttAAP-6

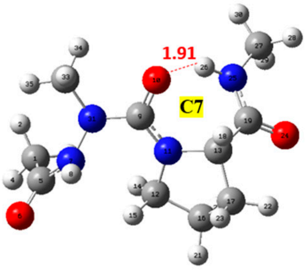

cttAAP-7

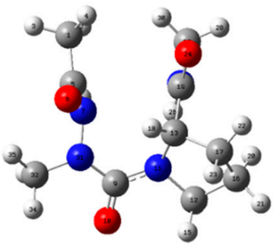

tctAAP-8

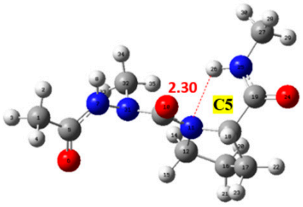

tttAAP-9

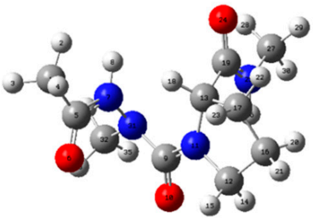

tctAAP-10

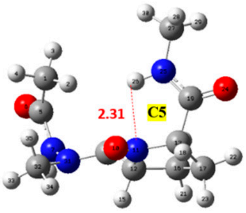

cttAAP-11

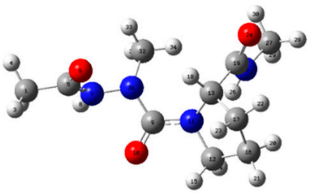

tctAAP-12

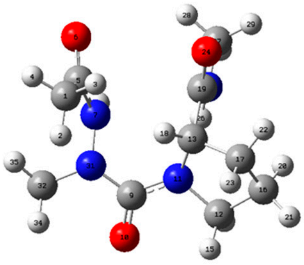

cctAAP-13

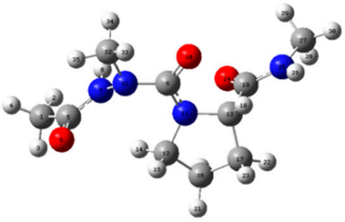

tttAAP-14

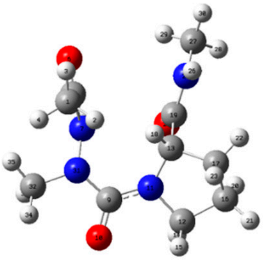

cctAAP-15

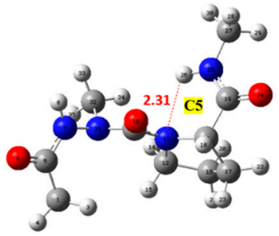

cttAAP-16

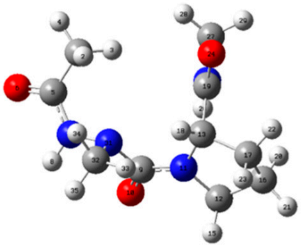

cctAAP-17

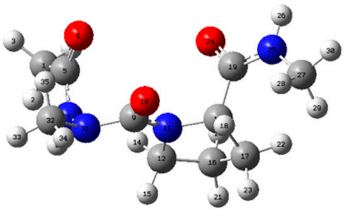

ttcAAP-18

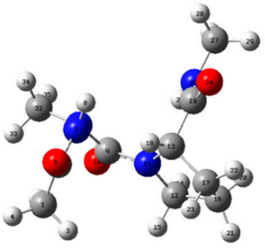

cctAAP-19

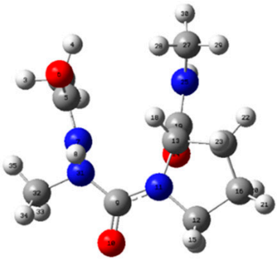

cccAAP-20

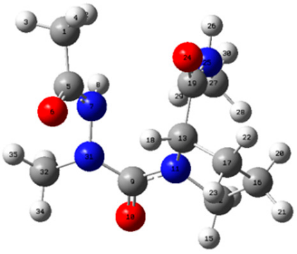

tccAAP-21

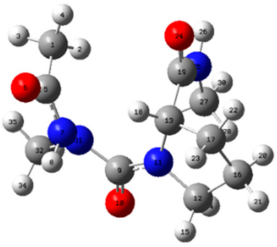

cccAAP-22

Figure S9. Hydrogen bonds (red dashed line) stabilizing the 22 optimized structures of Ac-azaAla-Pro-NHMe calculated in water at the SMD/B3LYP/6-311++G (d,p) level of theory.

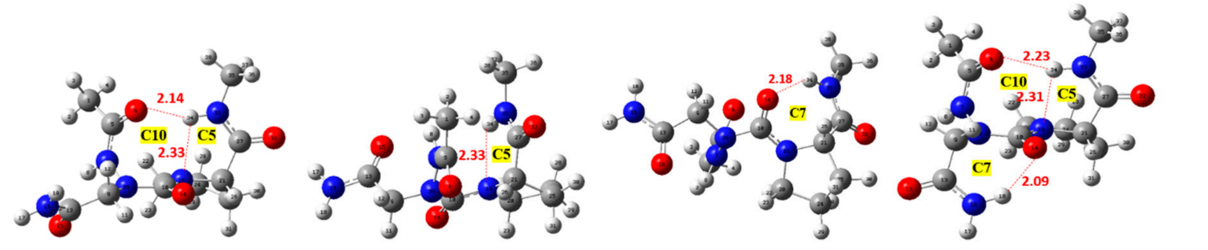

tttANP-1 **tctANP-2** **tttANP-3** **tttANP-4**

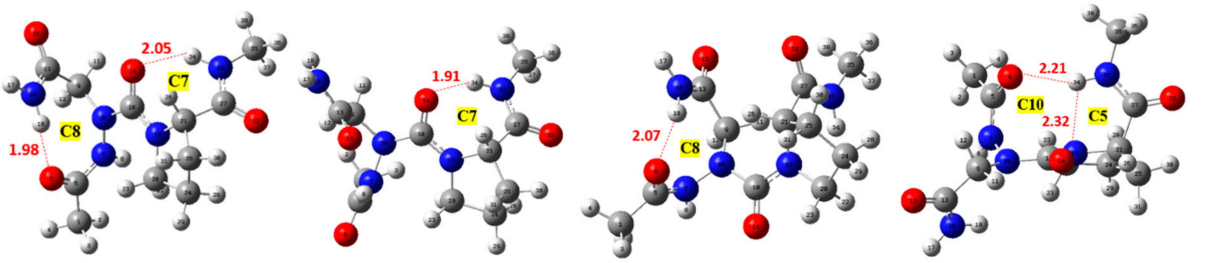

tttANP-5 **cttANP-6** **tctANP-7** **tttANP-8**

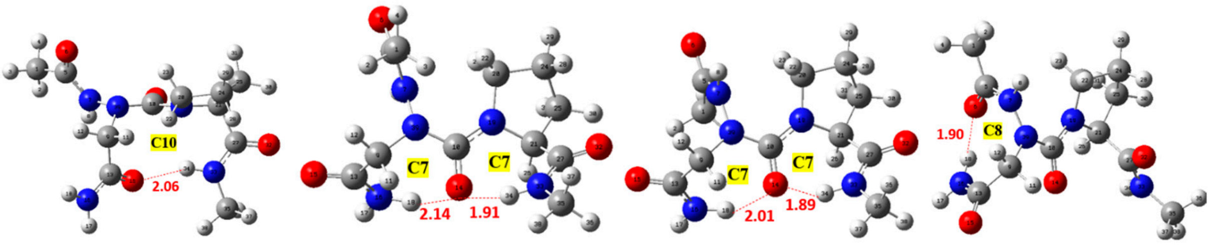

tttANP-9 **cttANP-10** **cttANP-11** **tttANP-12**

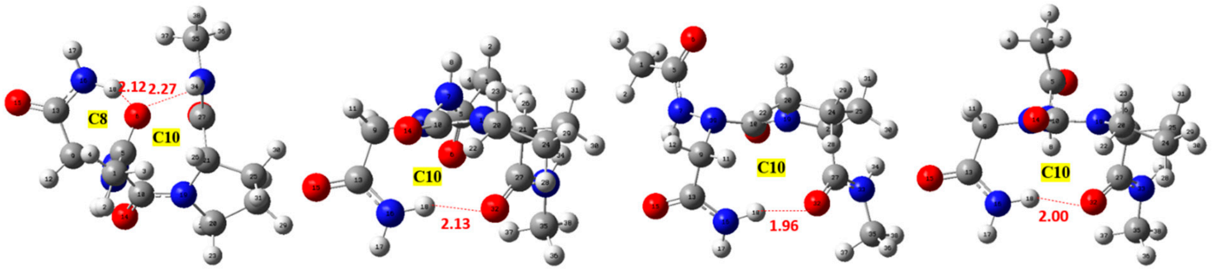

tctANP-13 **tctANP-14** **tttANP-15** **cctANP-16**

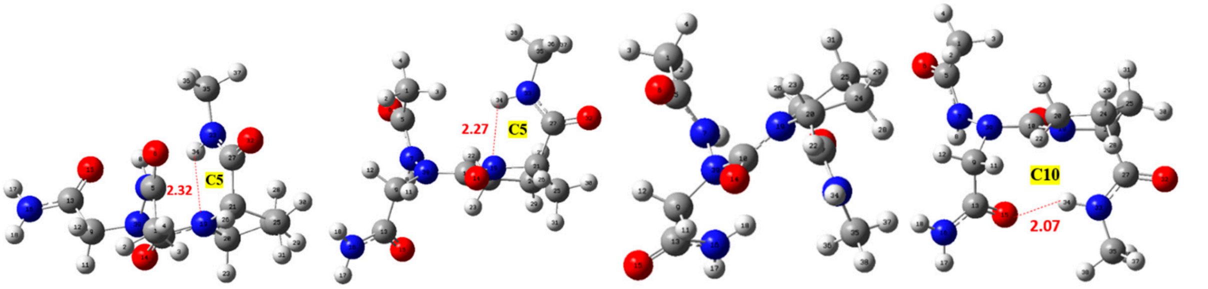

cctANP-17 **cttANP-18** **tctANP-19** **cttANP-20**

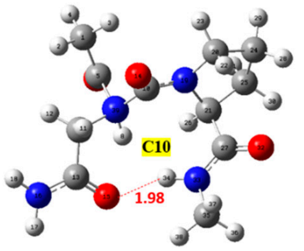

cctANP-21

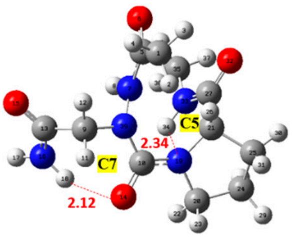

cctANP-22

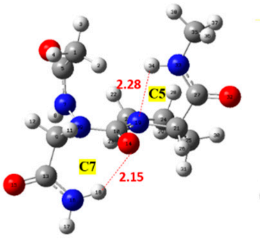

cttANP-23

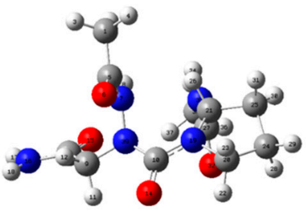

tctANP-24

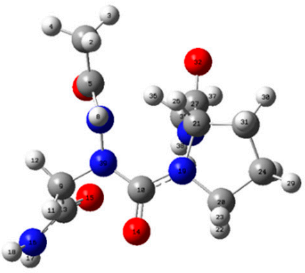

tctANP-25

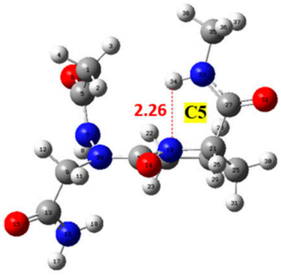

cttANP-26

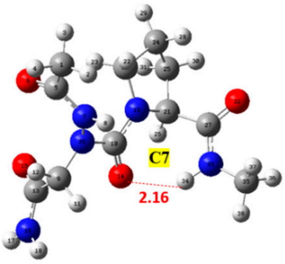

tttANP-27

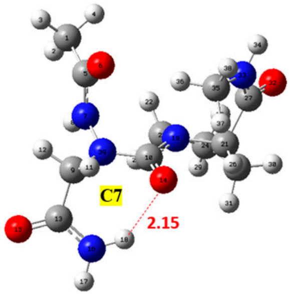

ttcANP-28

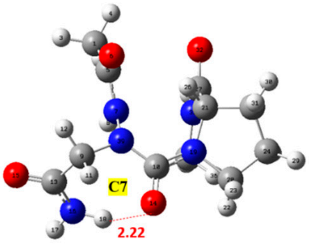

tccANP-29

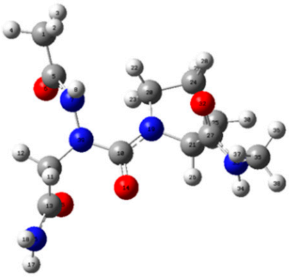

tttANP-30

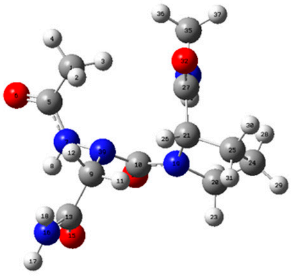

cctANP-31

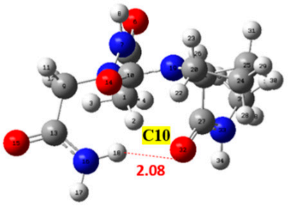

cccANP-32

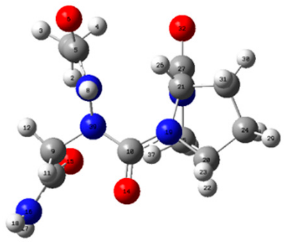

cccANP-33

Figure S10. Hydrogen bonds (red dashed line) stabilizing the 33 optimized structures of Ac-azaAsn-Pro-NHMe calculated in water at the SMD/B3LYP-D_3/6-311++G (d,p) level of theory.

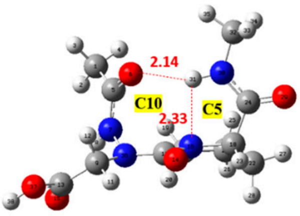

ttADP-1

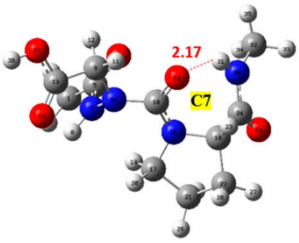

tttADP-2

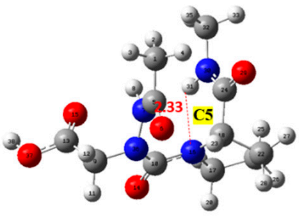

tctADP-3

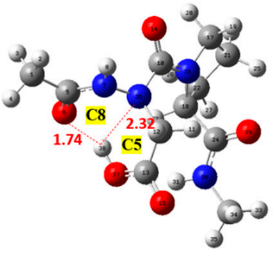

tctADP-4

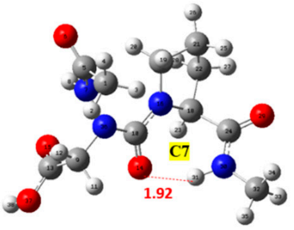

cttADP-5

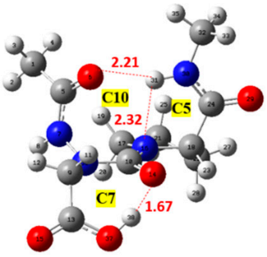

tttADP-6

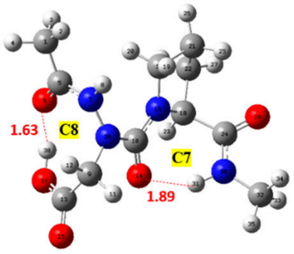

tttADP-7

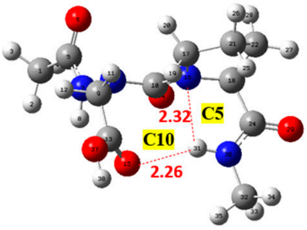

tttADP-8

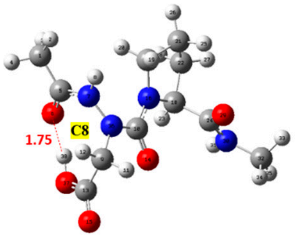

tttADP-9

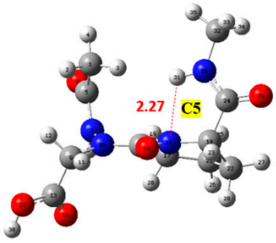

cttADP-10

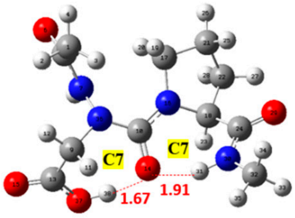

cttADP-11

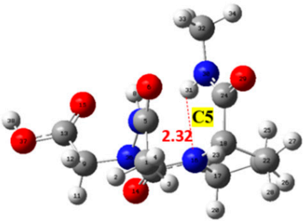

cctADP-12

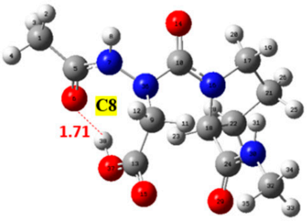

tctADP-13

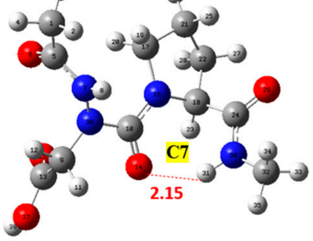

tttADP-14

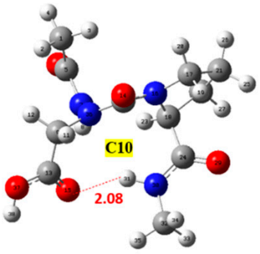

cctADP-15

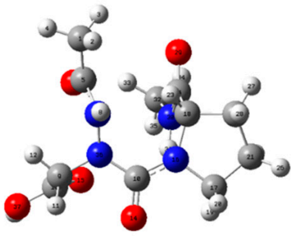
tctADP-16

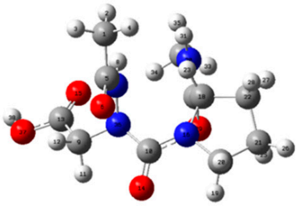
tctADP-17

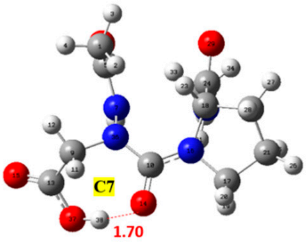

cctADP-18

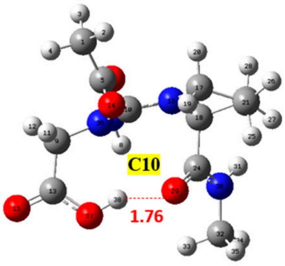

cctADP-19

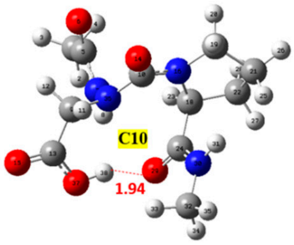

tctADP-20

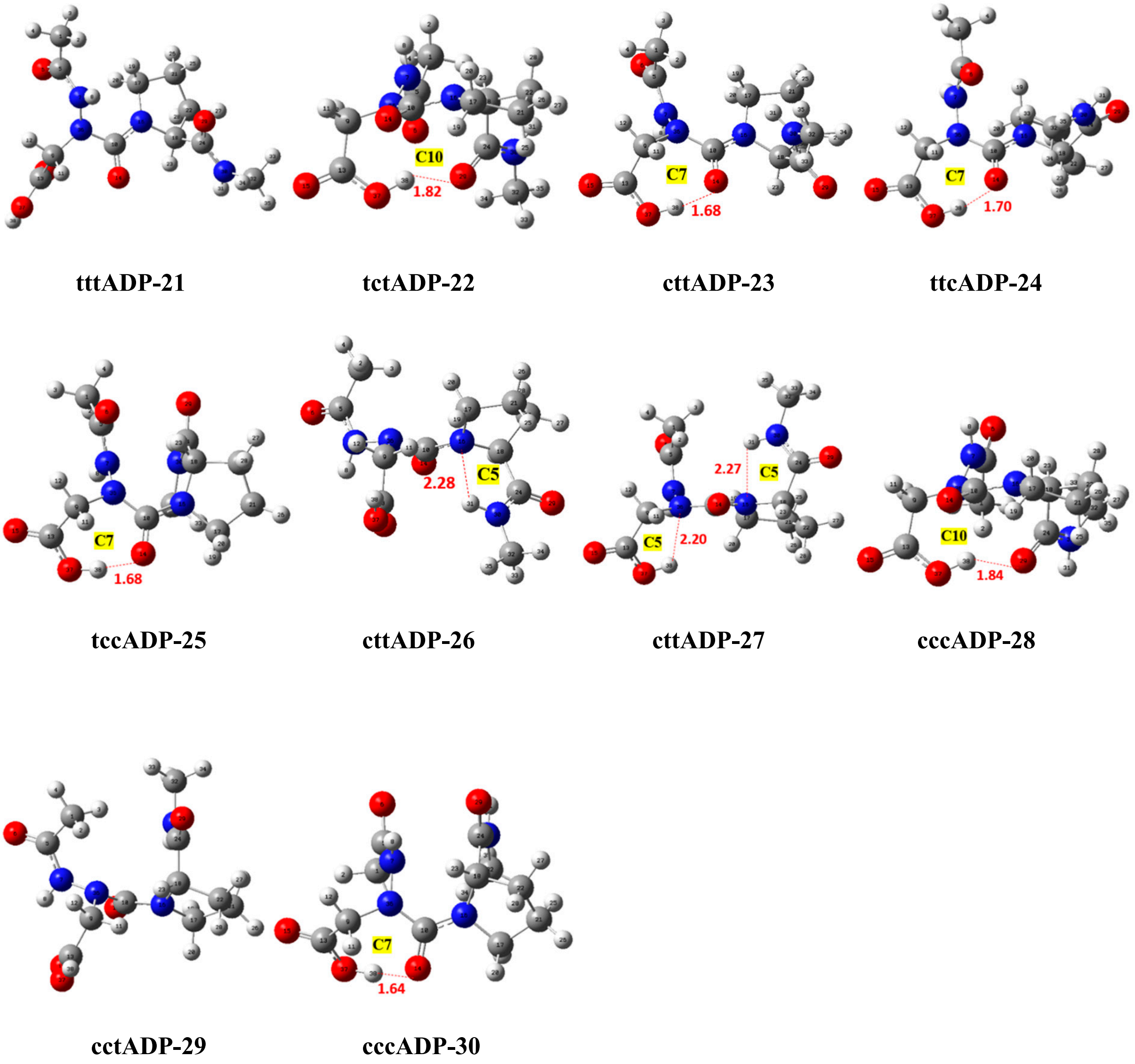

Figure S11. Hydrogen bonds (red dashed line) stabilizing the 30 optimized structures of Ac-azaAsp-Pro-NHMe calculated in water at the SMD/B3LYP-D$_3$/6-311++G (d,p) level of theory.

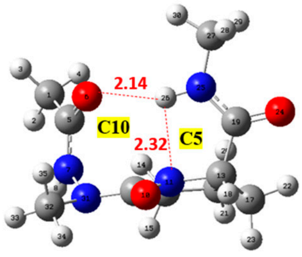

tttAAP-1

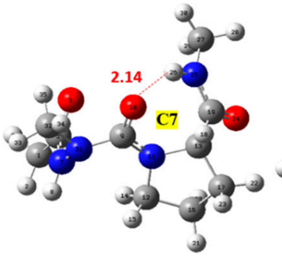

tttAAP-2

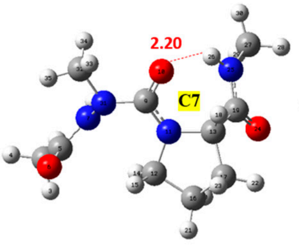

tttAAP-3

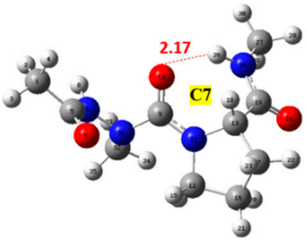

tttAAP-4

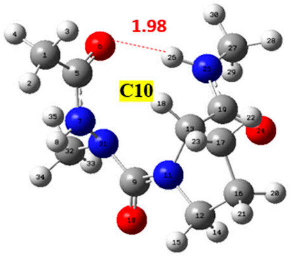

tctAAP-5

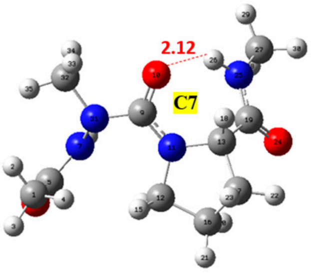

cttAAP-6

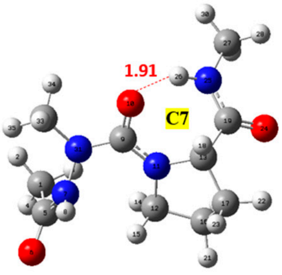

cttAAP-7

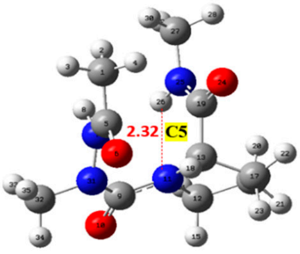

tctAAP-8

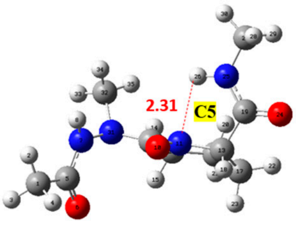

tttAAP-9

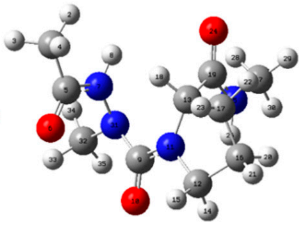

tctAAP-10

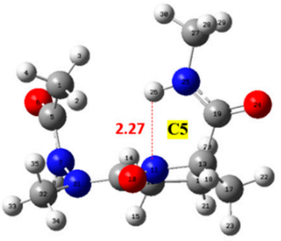

cttAAP-11

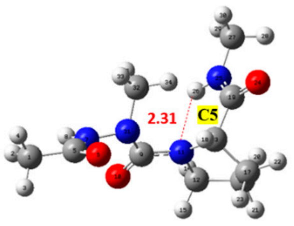

tctAAP-12

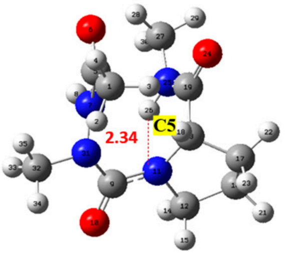

cctAAP-13

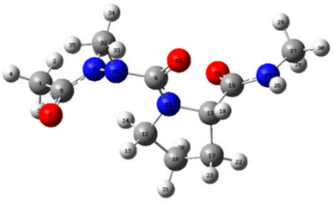

tttAAP-14

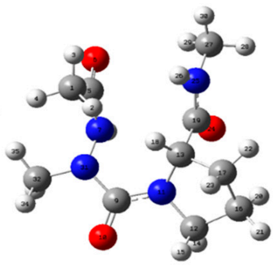

cctAAP-15

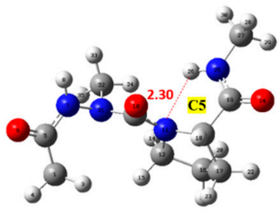

cttAAP-16

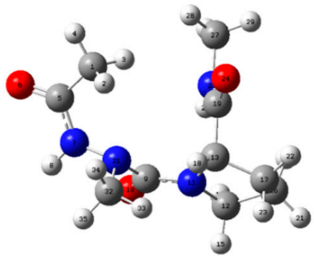

cctAAP-17

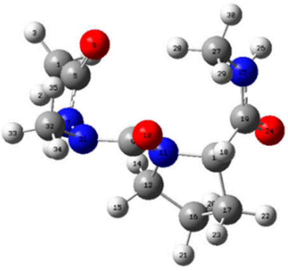

ttcAAP-18

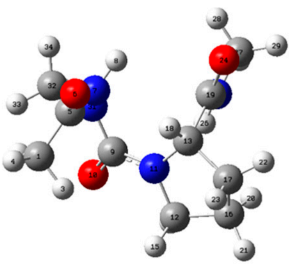

cctAAP-19

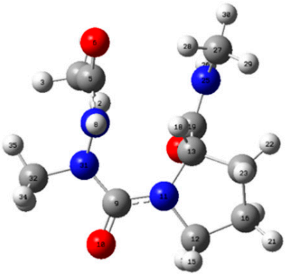

cccAAP-20

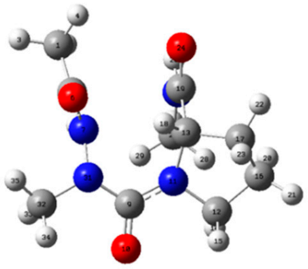

tccAAP-21

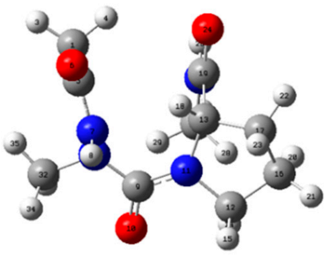

cccAAP-22

Figure S12. Hydrogen bonds (red dashed line) stabilizing the 22 optimized structures of Ac-azaAla-Pro-NHMe calculated in water at the SMD/B3LYP-D_3/6-311++G (d,p) level of theory.

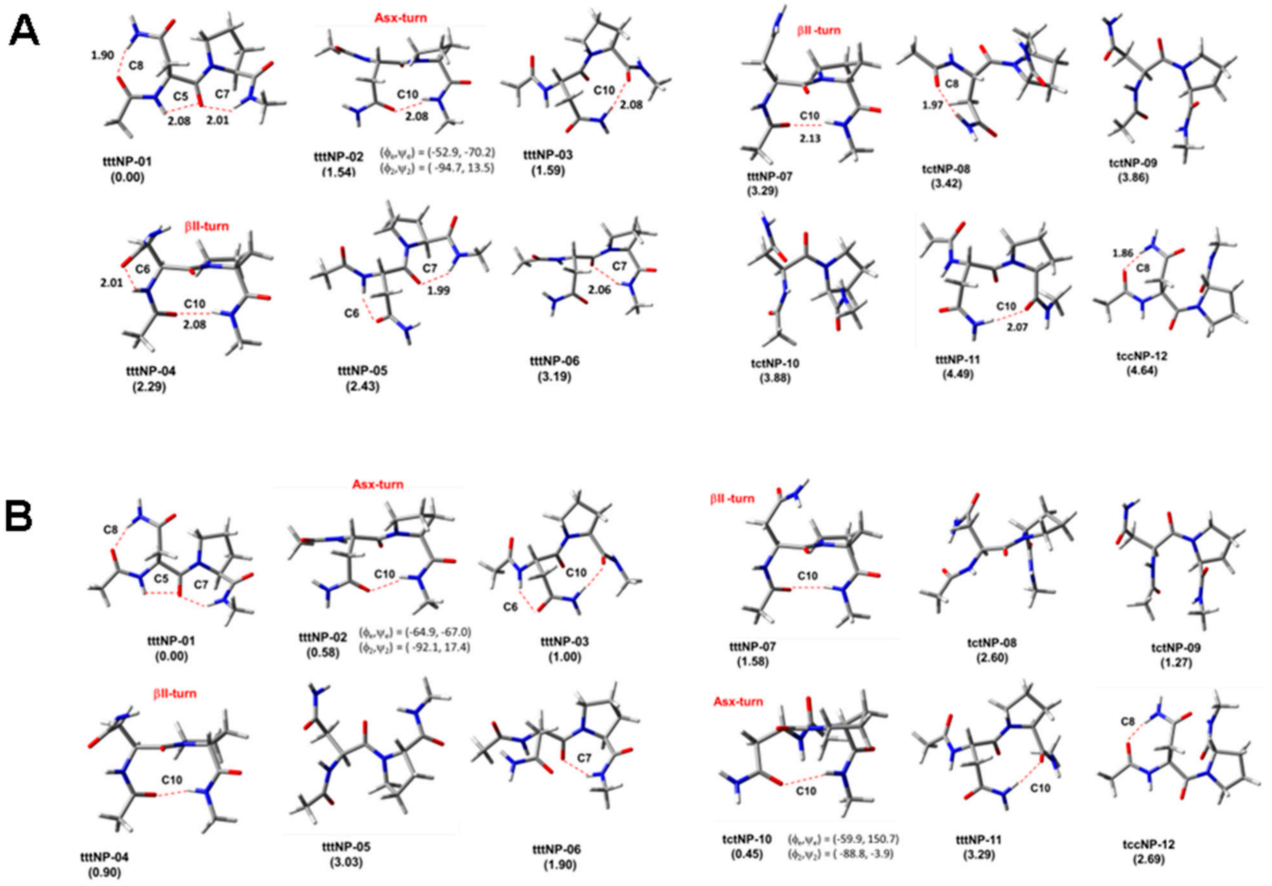

Figure S13. Hydrogen bonds (red dashed line) stabilizing the optimized structures of Ac-Asn-Pro-NHMe (**7**, **NP**) calculated at the B3LYP/6-311++G (d,p) (**A**) and B3LYP-D3/6-311++G(d,p) level of theory (**B**). The relative energies of the conformers are shown in parenthesis.

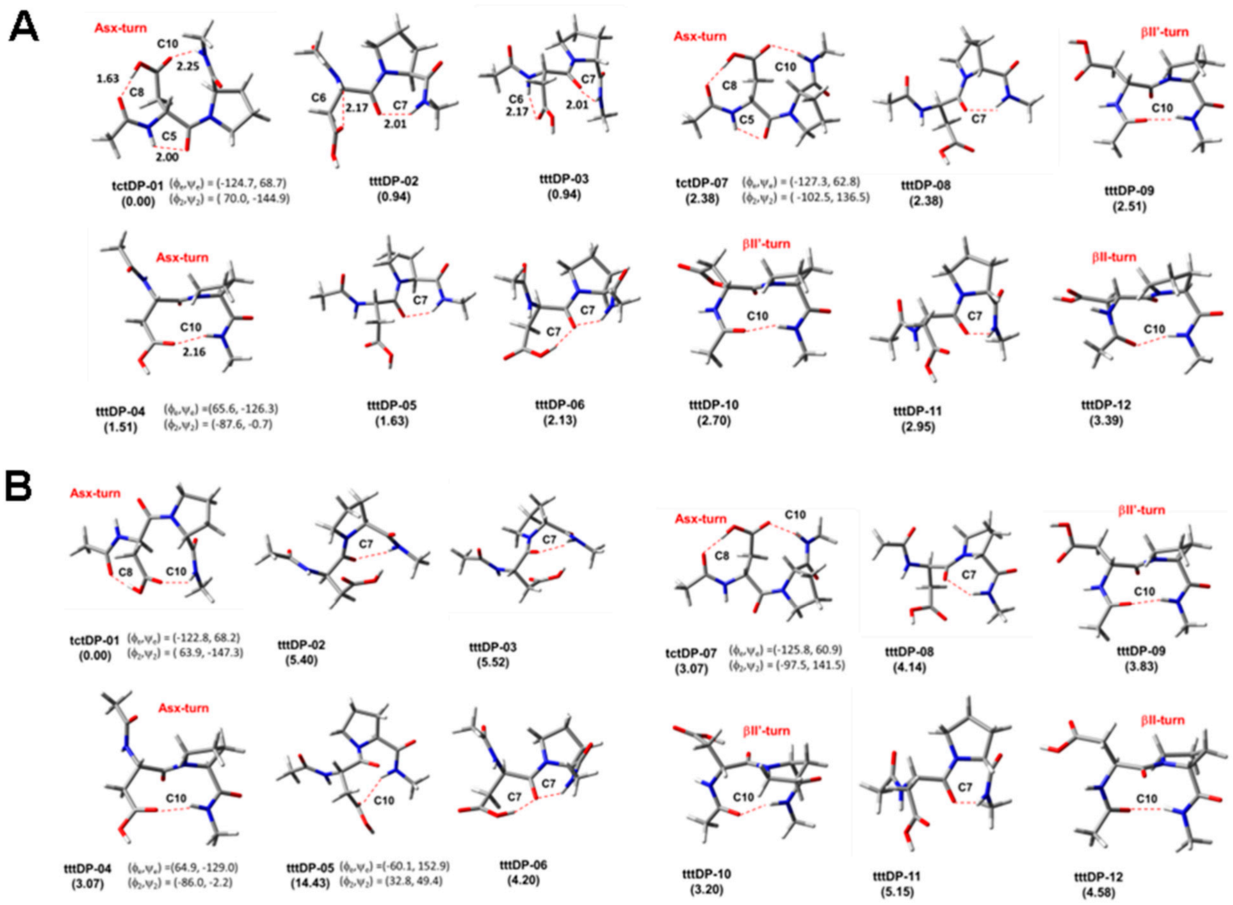

Figure S14. Hydrogen bonds (red dashed line) stabilizing the optimized structures of Ac-Asp-Pro-NHMe (**8**, **DP**) calculated at the B3LYP-D3/6-311++G (d,p) (**A**) and B3LYP-D3/6-311++G(d,p) (**B**) levels of theory. The relative energies of the conformers are shown in parenthesis.
